# The novel circular RNA CircMef2c is positively associated with muscle growth in Nile tilapia

**DOI:** 10.1016/j.ygeno.2023.110598

**Published:** 2023-03-09

**Authors:** Golam Rbbani, Artem Nedoluzhko, Prabhugouda Siriyappagouder, Fedor Sharko, Jorge Galindo-Villegas, Joost A.M. Raeymaekers, Rajesh Joshi, Jorge M.O. Fernandes

**Affiliations:** a Genomics Division, Faculty of Biosciences and Aquaculture, Nord University, 8049 Bodø, Norway; b Paleogenomics laboratory, European University at Saint Petersburg, 191187 Saint-Petersburg, Russia; c LLC ELGENE, 109029 Moscow, Russia; d GenoMar Genetics AS, 0252 Oslo, Norway

**Keywords:** Muscle growth, Circular RNA, microRNA, ceRNA network, Spliceosome, Nile tilapia

## Abstract

Muscle growth in teleosts is a complex biological process orchestrated by numerous protein-coding genes and non-coding RNAs. A few recent studies suggest that circRNAs are involved in teleost myogenesis, but the molecular networks involved remain poorly understood. In this study, an integrative omics approach was used to determine myogenic circRNAs in Nile tilapia by quantifying and comparing the expression profile of mRNAs, miRNAs, and circRNAs in fast muscle from full-sib fish with distinct growth rates. There were 1947 mRNAs, 9 miRNAs, and 4 circRNAs differentially expressed between fast- and slow-growing individuals. These miRNAs can regulate myogenic genes and have binding sites for the novel circRNA circMef2c. Our data indicate that circMef2c may interact with three miRNAs and 65 differentially expressed mRNAs to form multiple competing endogenous RNA networks that regulate growth, thus providing novel insights into the role of circRNAs in the regulation of muscle growth in teleosts.

## 1 Introduction

Aquaculture is a rapidly developing industry that plays a vital role in securing food for the increasing human population. According to recent global statistics, world aquaculture production reached another all-time high of 82 million tons in 2018 [1]. Growth is one of the most important traits in aquaculture, which directly benefit farmers and has been the key trait of selection for all the breeding programs across the species [2]. Fish growth is largely associated with muscle tissue, which constitutes around 40–60% of the edible body mass [3]. Fish muscle formation and growth include some fundamental events, namely the early muscle cell commitment, the development of different proportions of slow and fast muscle fibers, and hyperplasia (increase in fiber number) and hyper-trophy (increase in fiber size) throughout much of ontogeny [3,4]. These processes require a coordinated expression of many genes and diverse molecular pathways. In recent years, an increasing number of studies have suggested the importance of epigenetic mechanisms, including non-coding RNAs, in fish muscle development [5–10]. In-depth knowledge about regulatory mechanisms involved in fish growth is essential to explore the observed phenotypic variation in aquaculture conditions. Yet, our understanding of the complex regulatory networks involved in controlling fish muscle growth is still limited.

Living organisms transcribe thousands of non-coding RNAs, including microRNAs (miRNAs), small interfering RNAs (siRNAs), piwi-interacting RNAs (piRNAs), and long non-coding RNAs (lncRNAs) [11]. Together, they regulate gene expression at transcriptional and post-transcriptional levels. miRNAs inhibit gene expression mainly by pairing with complementary sequences in the 3’-UTR of their target mRNAs, thus inducing their degradation or translational repression. The role of miRNAs in muscle development, growth, and regeneration, as well as conditions such as atrophy, has been reported in teleosts [6,12,13]. More recently, it has been discovered that circRNAs alter both miRNA and mRNA expression, and play a crucial role in various physiological, biological, and molecular processes [14–16]. Exonic and exonic-intronic circular RNAs (circRNAs) are uncapped, non-polyadenylated, endogenous non-coding RNAs formed by back-splicing of precursor mRNAs. In contrast, intronic circRNAs are typically derived from spliced introns (lariats) that escape debranching and are circularized by 2′-5′ phosphodiester bonds. circRNAs are relatively resistant to exonucleases, making them more stable than the traditional linear mRNA counterparts.

Although the majority of circRNAs identified in different species still lack functional annotation, there is growing evidence that circRNAs participate in epigenetic, transcriptional, and post-transcriptional regulation of gene expression [14,17,18]. CircRNAs also act as protein sponges and competing endogenous RNA (ceRNA) in various organisms, thus regulating phenotypic expression. ceRNA is a post-transcriptional gene regulation mechanism in which circRNAs and mRNAs crosstalk and compete for shared target miRNAs. CircRNAs usually contain microRNA-responsive elements by which they modulate miRNA activity, thereby reducing their availability to bind to the targeted protein-coding transcripts. Moreover, some circRNAs contain binding sites for multiple miRNAs or multiple sites for a single miRNA type/family, often referred to as a miRNA sponge; therefore, circRNA-miRNA-mRNA (ceRNA) networks may influence various biological pathways and the expression of many genes [19]. The role of circRNAs in muscle growth has been described in different livestock species reviewed by [3]. For example, circSNX29 in bovine skeletal muscle serves as an endogenous miR-744 sponge and promotes myoblast differentiation [20]. In contrast, there is a single report about circRNAs in teleost muscle, suggesting that a novel_circ_0002886 in snout bream (*Megalobrama amblycephala)* may act as ceRNA and inhibit the apoptosis of skeletal muscle cells [21]. Therefore, there is a need to characterize ceRNA networks to understand the processes regulating muscle growth in teleosts.

Nile tilapia (*Oreochromis niloticus*) is among the most studied teleosts, since it is the second most important farmed fish worldwide [1]. Nowadays, selective breeding programs for various tilapia species and their hybrids are undergoing to improve physiological and development processes, such as growth and disease resistance [2,22]. Thus, understanding the molecular mechanisms underlying different biochemical and physiological processes affecting muscle growth is one of the central themes of genetic improvement; and the paper aims to investigate the potential regulatory mechanisms of circRNAome in muscle. Our results provide the first overview of circRNA, miRNA, and mRNA co-expression in a regulatory network with possible functional implications for the regulation of muscle growth in teleosts.

## 2 Results

### 2.1 Experimental groups and summary of sequencing data

**Fig. 1:**
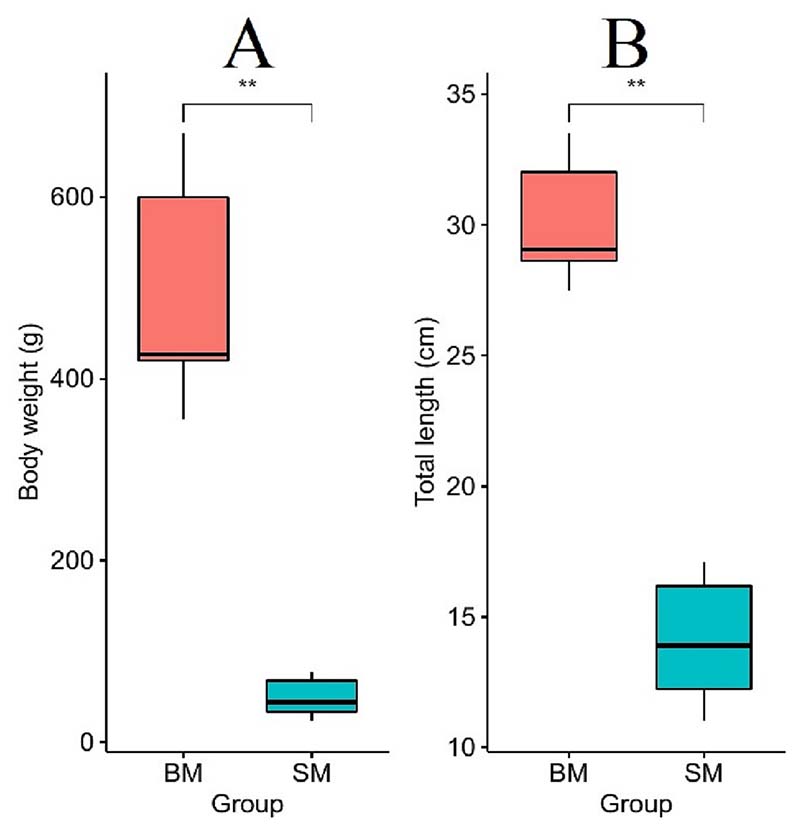
Length and weight of slow- and fast-growing fish. Difference in body weight and total length at 9 months between slow- (SM) and fast-growing (BM) groups of Nile tilapia used in this study. BM group differences are shown by red boxplot, SM group differences are marked in blue. (For interpretation of the references to colour in this figure legend, the reader is referred to the web version of this article.)

The growth performance of the Nile tilapia full-sibs is shown in Fig. 1. The mean total length and mean body weight of BM (Big male, i. e., fast-growing) were 2.1- and 10.0-fold higher than their SM (Small male, i.e., slow-growing) counterparts, respectively. Individual libraries of BM and SM groups were sequenced (Tables S1-S3 in Supplementary Material), and a total of 964,592,220 read pairs, 660,513,692 read pairs, and 319,445,174 single reads were generated from mRNA-seq, circRNA-seq and miRNA-seq data, respectively (Table 1). Trimming of adapter and low-quality reads yielded 938,496,548 mRNA read pairs, 623,923,612 circRNA read pairs, and 276,419,289 miRNA read pairs with quality > Q20. The average mapping rate of clean reads to the Nile tilapia reference genome was 93.9% mRNA, 91.3% circRNA, and 58.9% miRNA. Detailed analysis showed that around 73% and 92.5% of transcripts are mapped in the correct position and orientation (i.e., both mates of a read pair map to the same chromosome, oriented towards each other) for mRNA and circRNA, respectively. Mapped read counts were used in further differential expression analyses.

### 2.2 Identification of differentially expressed mRNAs (DE-mRNAs) between slow- and fast-growing males

**Fig. 2:**
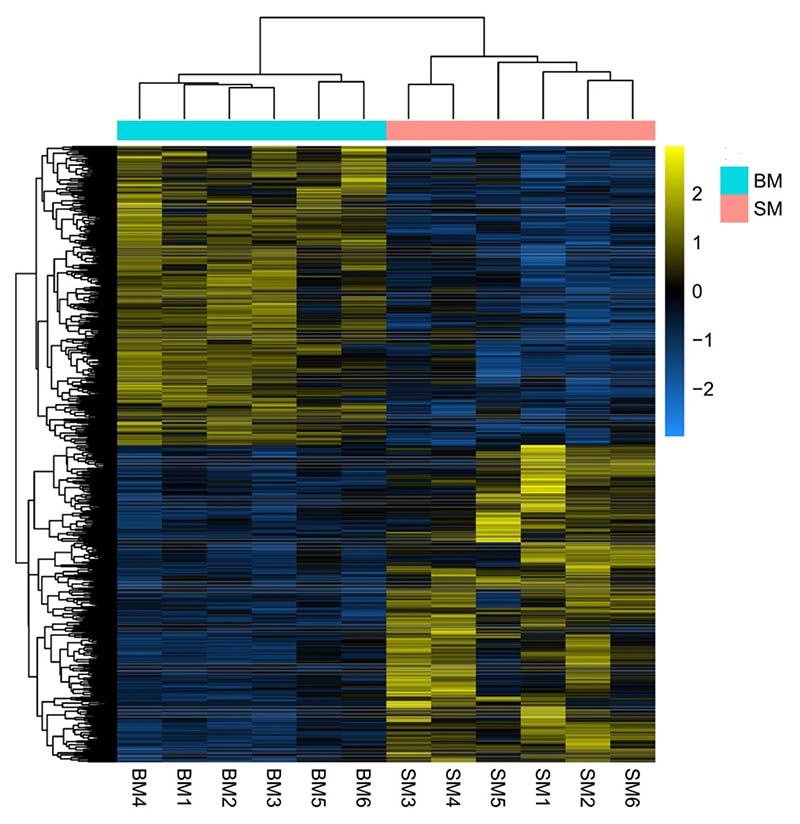
Muscle transcriptome differences between fast-growing (BM) and slow-growing fish (SM). Heatmap of differentially expressed mRNAs in Nile tilapia fast muscle (BM-fast growing, SM-Slow growing). The colour scale represents the difference in expression with an adjusted *p-value* ≤ 0.05 and |Log_2_fold change| ≥1.

We retrieved a total of 1947 differentially expressed mRNAs (DE-mRNAs) with |Log_2_fold change| ≥1 and *p-adjusted value* ≤ 0.05. The complete list of the identified genes is reported in Supplementary Table S4. Among the 1947 DE-mRNAs, 1002 were up-regulated, and 945 were down-regulated in SM group compared to BM. The volcano plot illustrates the changes in mRNA expression (Supplementary Fig. S1). To confirm if the transcriptome diversity described above reflects genuine expression differences in muscle or not, we performed a principal component analysis. The first principal component (PC1) accounts for 33% of the variance in our data and separates samples into two groups (Supplementary Fig. S2). It also reflects that fast-growing fish have more individual differences than their slow-growing counterparts. Further, hierarchical clustering of DE-mRNAs provided an overview of the expression patterns and showed a clear separation between groups (Fig. 2). Several growth-associated protein-coding genes, including *igf2bp2, tgfbi, myod1, myf5, tnfsf12, adamts16, asb2, tmod4,* and *hacd1*, were significantly less expressed in the slow-growing than in the fast-growing group.

### 2.3 Genes up-regulated in slow-growing Nile tilapia are enriched in splice-related GO terms and the spliceosome pathway

**Fig. 3:**
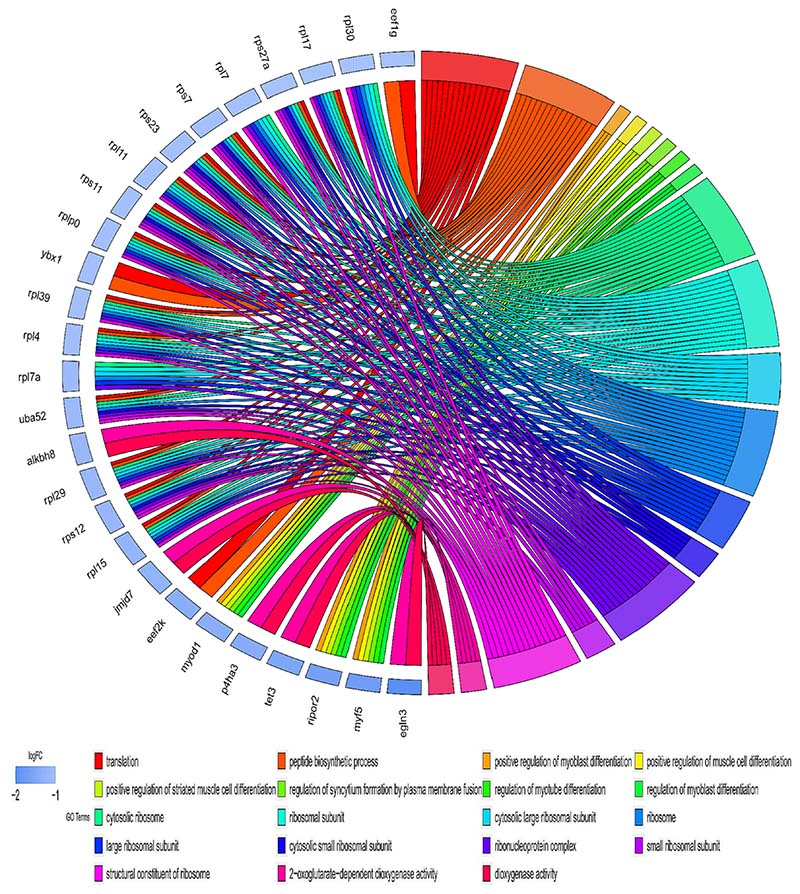
Gene ontology (GO) enrichment of down-regulated mRNA. The chord diagram shows the relationship between some enriched terms and the log_2_ fold change. The gradient colour bar represents the Log_2_ fold change (adjusted *p-value* ≤ *0.05* and |Log_2_ fold change| ≥ 1).

**Fig. 4:**
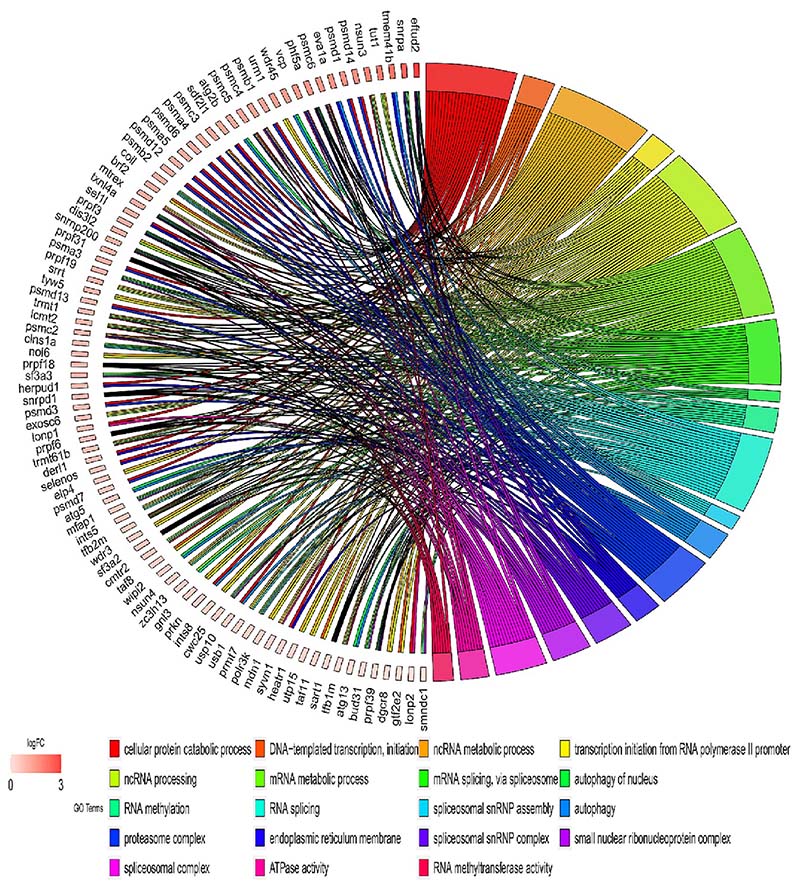
Gene ontology (GO) enrichment of up-regulated mRNA. The chord diagram shows the relationship between significantly enriched terms and the log_2_ fold change. The gradient colour bar represents the Log_2_ fold change (adjusted *p-value* ≤ *0.05* and |Log_2_ fold change| ≥ 1).

Gene ontology (GO) analysis of differentially expressed mRNA, which includes biological process, molecular function, and cellular component, was conducted to understand the potential involvement of DE mRNAs in molecular pathways. The result demonstrated that the down-regulated genes in the SM group are significantly enriched in some important functional terms, including regulation of muscle cell differentiation, myoblast and myotube differentiation, positive regulation of striated muscle cell differentiation, ribonucleoprotein complex ribosome (Fig. 3). Conversely, up-regulated genes in the SM group are enriched in mRNA splicing via spliceosome, spliceosomal small nuclear ribonucleoproteins (snRNPs) complex, spliceosomal tri-snRNP complex, transcription initiation from RNA polymerase II promoter, autophagosome, proteasome complex (Fig. 4). Multiple splice-related GO terms imply shifts in the splicing pattern of splicing-related genes that encode the spliceosome and its accessory proteins. Detailed analysis of the different terms revealed several genes associated with multiple GO terms, namely *myf5, moyd1, trmt1l, rpl9, fxr1, sf3a3, prpf6, txnl4a, optn* (Fig. S3A and S4B in Supplementary Material).

**Fig. 5:**
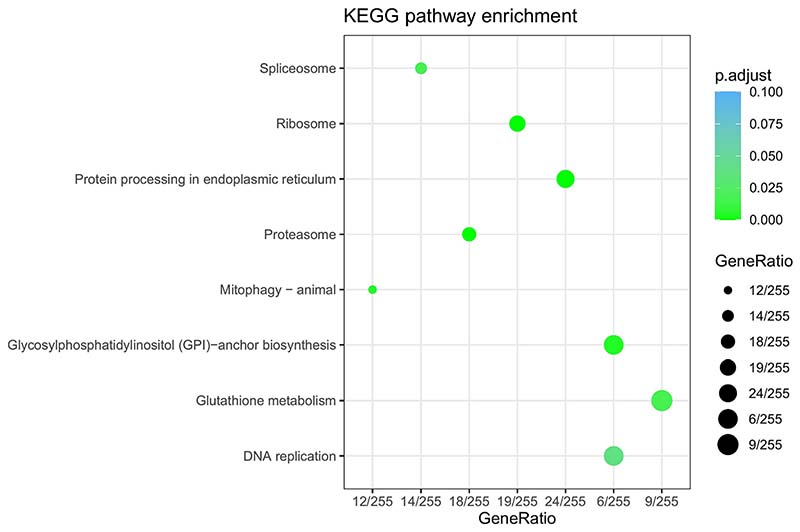
KEGG pathways altered between fast-growing (BM) and slow-growing fish (SM). The scatter diagram shows significant pathway enrichments for DE-mRNAs. Gene ratio is the number of DE-mRNAs in this pathway to all the genes in this pathway. The X-axis corresponds to the gene ratio of pathway, and the Y-axis represents a different pathway. The colour intensity of the nodes shows the degree of enrichment, dot size represents the count of genes in a pathway and the q-value is colour-coded.

**Fig. 6:**
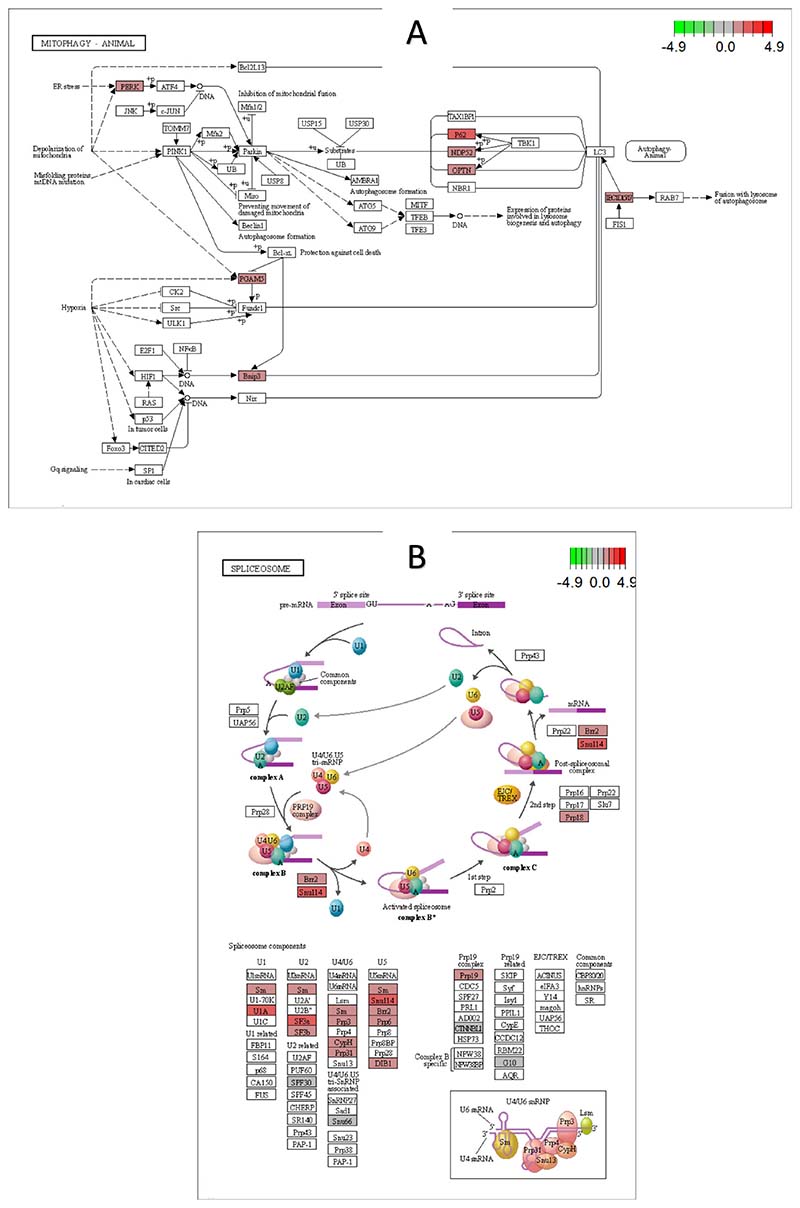
Graphical representation of (A) spliceosome and (B) mitophagy KEGG pathways. The boxes represent mRNAs in the pathway, with those in red being differentially expressed between BM and SM groups. (For interpretation of the references to colour in this figure legend, the reader is referred to the web version of this article.)

KEGG analysis revealed several significantly changed pathways between the groups with an adjusted *p-value* ≤ 0.05 (Fig. 5). Among the top enriched pathways, spliceosome, protein processing in the endoplasmic reticulum, mitophagy (Fig. 6A), and proteosome suggest that these genes may play a vital role in muscle growth through their involvement in myofiber degeneration, specific circRNA/gene or protein production and degradation. The spliceosome (Fig. 6B) contributes to splicing regulation by modulating splice site choice from pre-mRNA transcripts. The differentially expressed genes *snu114, sf3a,* and *u1a* are essential components for snRNPs suggesting a significant impact on pre-mRNA splicing process.

### 2.4 Multiple splice variants are expressed in Nile tilapia muscle

**Fig. 7:**
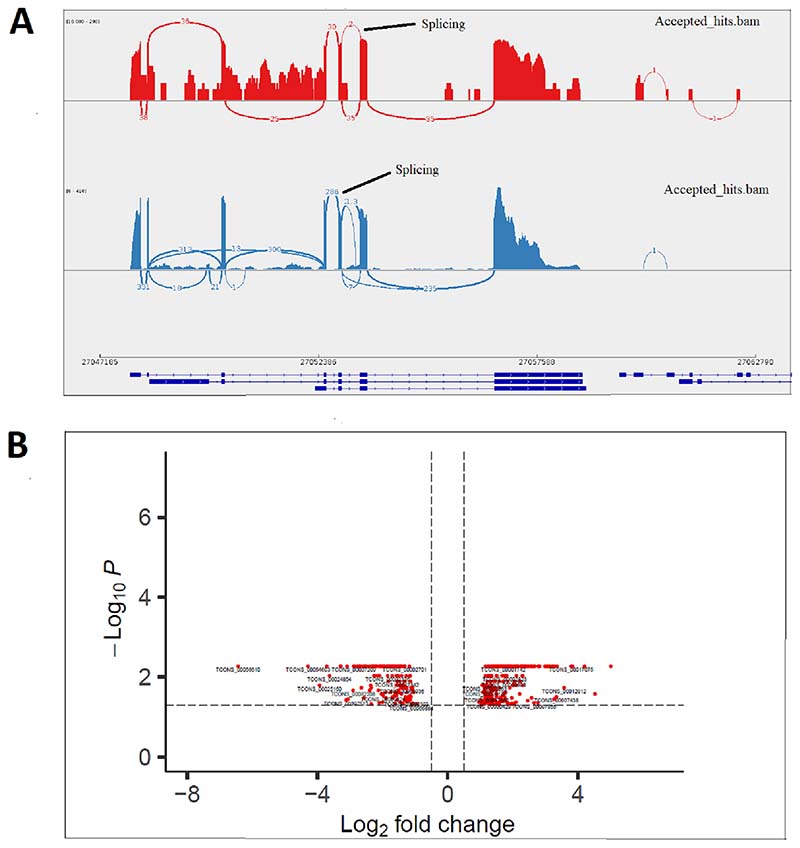
Comparison of alternative splicing between fast-growing (BM) and slow-growing groups. A) IGV-Sashimi plot for alternatively spliced exon and flanking exons of *dusp22* in two samples (blue (SM) and red (BM)). Regions in genomic coordinates are plotted on the x-axis, and read density (whose value is configurable via IGV) on the y-axis. B) Volcano plot of expressed gene isoforms in Nile tilapia fast muscle. Significant (*p-value* ≤ 0.05) and non-significant isoforms are marked with red and black dots, respectively. (For interpretation of the references to colour in this figure legend, the reader is referred to the web version of this article.)

An average of 85.5% concordant read pairs were uniquely mapped and used for subsequent analysis. Expression levels of different isoforms were estimated based on Fragments Per Kilobase of transcript per Million (FPKM) mapped reads (Supplementary Fig. S4). We were able to detect a total of 59,119 splicing events from 42,621 genes. IGV-Sashimi plot shows the genomic region containing the alternative exons of *dusp22* gene (NC_031981.2:27,054,572-27,060,684) (Fig. 7A). Besides, 845 transcription start sites were significantly differentially expressed, describing the transcription initiation and regulation variability at the transcriptional level. A total of 113,949 splice variants were expressed in at least one of the 12 samples (arbitrary cutoff with reads per kilobase of exon per million reads mapped ≥0.1), and among them, 423 were significantly differentially expressed between BM and SM groups (Fig. 7B). For example, *znf414, mapk6, moyd1, sparc,* and *tgfbi* were among the genes producing isoforms related to muscle growth. Moreover, 585 differentially expressed coding sequences were found between the groups from all isoforms of the expressed gene attributes.

### 2.5 Skeletal muscle myomiRs are expressed at different levels between slow- and fast-growing Nile tilapia

**Fig. 8:**
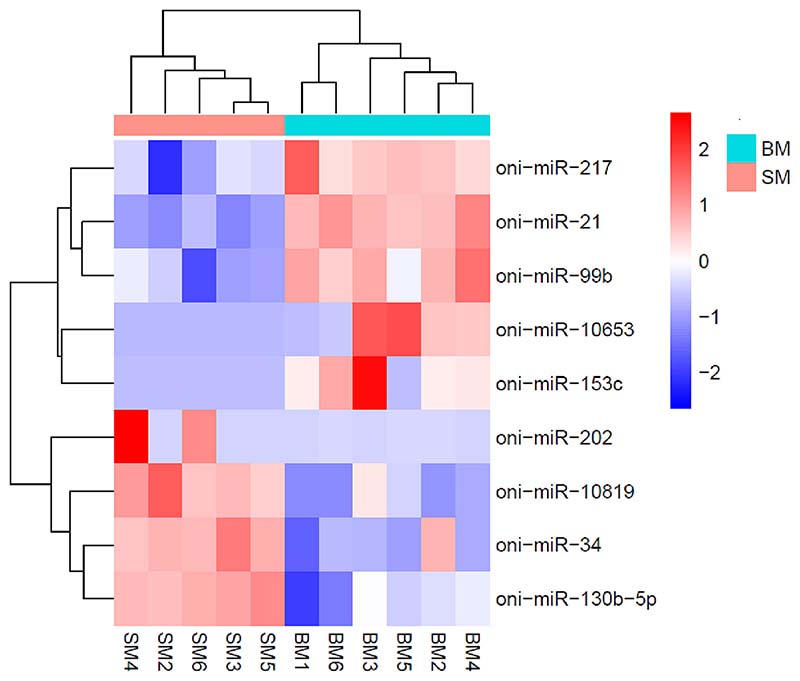
Heatmap of differentially expressed miRNAs in Nile tilapia fast muscle. The change in colour represents the difference in expression between slow- (SM) and fast-growing (BM) fish, with an adjusted *p-value* ≤ 0.05 and |Log_2_fold change| ≥1.

The majority of small RNA reads in each library were between 17 and 33 nucleotides (nt) long (Fig. S5 in Supplementary Material), with a marked peak at 22 nt. The average mapping efficiency of miRNA reads was 58.9% (Table 1). Subsequently, the expression levels of miRNAs were calculated based on the read count and were normalized to identify differentially expressed miRNA. As shown in Fig. 8, a total of 9 miRNAs significantly differentially expressed miRNAs were identified with a stringent threshold (q-value ≤0.05). The statistical significance of miRNA expression ratios against their q-values is shown in Supplementary Fig. S6. Furthermore, PCA analysis confirms the clustering of BM and SM groups, where the first principal component (PC1), explained 34% variability in the data (Supplementary Fig. S7). Interestingly, muscle-related oni-miR-202, oni-miR-21, oni-miR-217, oni-miR-34, oni-miR-731 and oni-miR-99b were significantly differentially expressed between the groups analyzed.

### 2.6 Four novel circRNAs are differentially expressed with size in Nile tilapia fast muscle

**Fig. 9:**
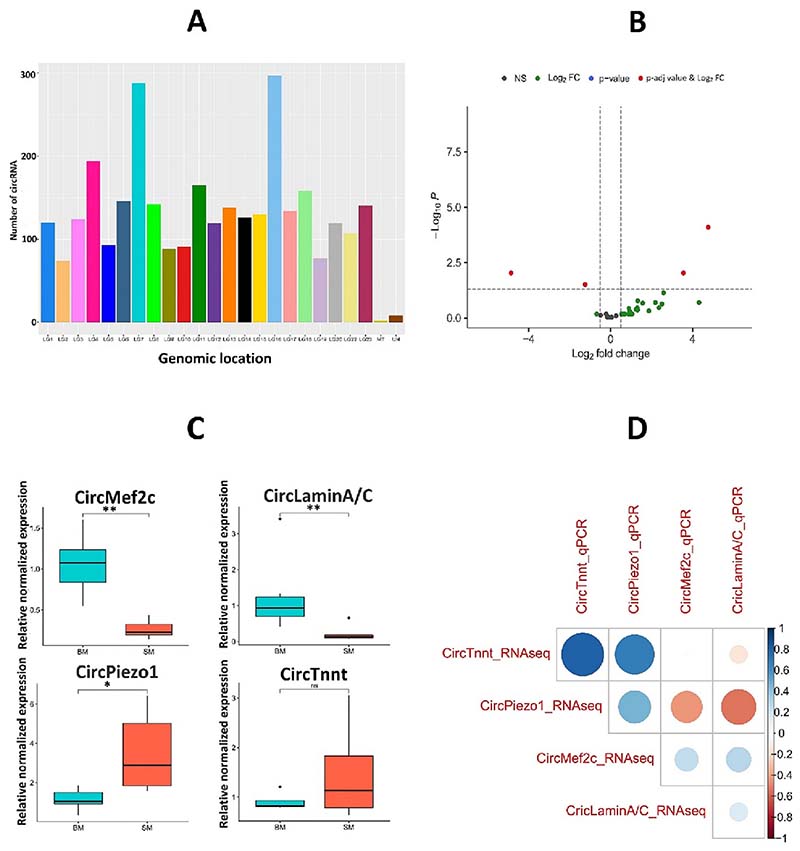
Characterization and expression profile of circRNAs. A) Distribution of circRNAs in different linkage groups (LG). MT – mitochondrial genome, UN – Unknown. Combined data for BM and SM Nile tilapia groups. B) Volcano plot showing differential expression of circular RNAs between BM and SM groups. Red points represent up- and down-expressed circRNAs with a padj-value below 0.05 and |Log_2_fold change| ≥1. The black dots represent non-significant results, green indicates genes having |Log_2_fold change| ≥1, and blue represents genes with a padj-value ≤0.05. C) qPCR analysis of differentially expressed circRNAs. circRNA expression levels were normalized using the ΔΔCT method, considering the geometric mean of two reference genes (*β-actin* and *elongation factor 1-alpha*). *p < 0.05;**p < 0.01 D) Correlation between the normalized counts from the circRNA sequencing data and relative gene expression from qPCR data. Differential expression results from next-generation sequencing data were normalized with DESeq2, which performs an internal normalization where geometric means are calculated for each gene across all samples. The colour scale indicates Pearson’s correlation coefficient. (For interpretation of the references to colour in this figure legend, the reader is referred to the web version of this article.)

The number of distinct circRNAs identified in each library varied between 62 and 470 (Fig. S8 in Supplementary Material). SM had a comparatively higher number of circRNAs than BM, indicating different circRNAomes between groups. However, these circRNAs were derived from both sense- and anti-sense strands. The number of circRNAs was not uniformly distributed across all the linkage groups (Fig. 9A). The highest number of circRNAs was produced from LG16 and LG7, whereas LG2, LG19, and the mitochondrial genome produced the least number of circRNAs. The size distribution of circRNAs ranged from 45 to over 2000 nt; 51.5% of circRNAs had a predicted spliced length of <2000 nt; 14.9% had a length 1000–2000 nt, and 36.5% had a length under 1000 nt (Fig. 9B). Host gene annotations results showed that most genes produce one circRNA rather than producing more than one circRNA. Alternative splicing events were observed in the muscle circRNAs. For example, two circRNAs derived from LG11 started at position 30,158,064 that ended at positions 30,166,226 and 30,171,209.

We conducted a principal component analysis to confirm the circRNA diversity between BM and SM groups. The first (PC1) and second (PC2) principal components account for 19% and 16% of the variance in our data, respectively, and separate the data in two groups (Supplementary Fig. S9). A total of 4 circRNAs were differentially expressed between BM and SM groups with padj-value ≤0.05 and | Log_2_fold change| ≥ 1 as the threshold. Among the DE-circRNAs, two were significantly down-regulated, and two were statistically up-regulated in SM group. The distribution of differentially expressed circRNAs is shown in a volcano plot (Fig. 9C). These DE-circRNAs were further classified into exonic and exon-intronic circRNAs. The detailed features and structural patterns of differentially expressed circRNAs between BM and SM groups are summarized in Table 2. Circ_NC_031972.2:9033630–9,035,959 produced from *myocyte-specific enhancer factor 2C-* was down-regulated by 1.2-fold, whereas circRNA NC_031972.2:48086230–48,086,927 associated with *troponin T* was up-regulated by 4.7-fold in slow-growing fish compared to their fast-growing counterparts. These circRNAs might play an important role in muscle growth in Nile tilapia.

### 2.7 Validation of circRNA, mRNA, and miRNA expression by qPCR

All the differentially expressed circRNAs were quantified using qRT-PCR. Divergent primers of length ˜ 20 nucleotides were designed to amplify exclusively the circRNA isoform (Table 3). The relative expression patterns of four circRNAs were consistent with the trends obtained from circRNA sequencing data (Fig. 9D and E). Furthermore, we used PCR and Sanger sequencing to confirm the back-splice junctions of circMef2c (Fig. S10 in Supplementary Material). The circular isoforms for all the Nile tilapia fast muscle samples tested could be amplified with these divergent primers, resulting in a ~ 300 bp amplicon, whereas no product was obtained in the negative control (RNAse-free water) (Supplementary Fig. S11).

Similarly, the expression analyses performed on the 15 selected mRNAs and 8 miRNAs yielded results that followed the trends in transcriptomic data (Supplementary Figs. S12 and S13).

### 2.8 Genes differentially expressed with size may form a circRNA–miRNA–mRNA competitive network involved in muscle growth

**Fig. 10:**
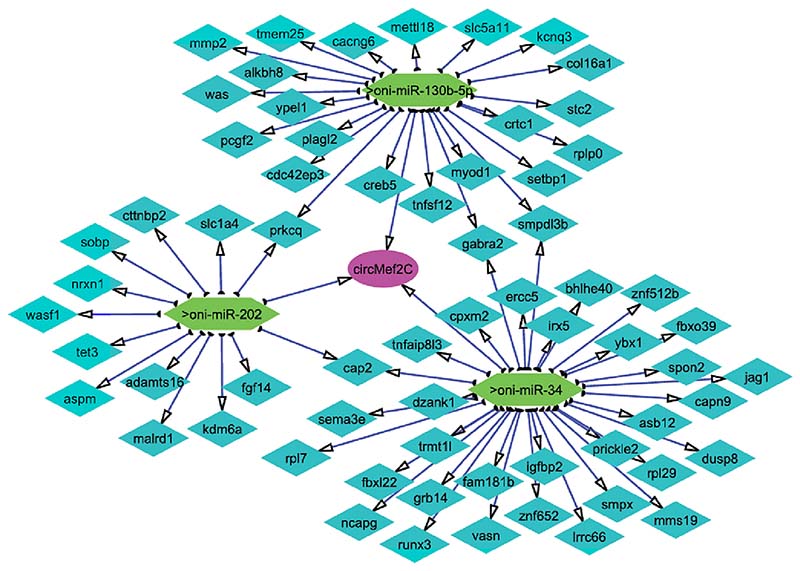
CircRNA–miRNA–mRNA regulatory network. The network consists of one circRNA(circMef2c), three miRNAs (on-miR-34, oni-miR-130b-5p, oni-miR-202) and 65 protein-coding genes.

An integrative analysis of the interplay among the three classes of RNA was performed based on differential expression analysis to elucidate their functional connections in Nile tilapia muscle growth. The putative target interactions between DE-circRNA, DE-miRNA, and DE-miRNA-DE-miRNA were predicted. Down-regulated genes correlate with up-regulated miRNA and down-regulated circRNAs, whereas up-regulated genes correlate with downstream miRNA and upstream circRNAs. The circRNA–miRNA–mRNA competitive network (ceRNAs) was constructed by combining circRNA-miRNA pairs with miRNA-mRNA interactions (Fig. 10). The network contains one circRNA, 3 miRNAs, and 65 mRNAs, providing a comprehensive perspective into the links between circRNA, miRNA, and mRNAs in muscle growth.

## 3 Discussion

With the recent development of transcriptome sequencing technology, circRNAs have been found with different expression patterns in multiple tissues to exert specific roles in various bioprocesses, including embryonic development and heat stress response. To the best of our knowledge, there is no comprehensive study to describe the complex regulatory role of circRNAs in teleost muscle. This study, for the first time, identifies and characterizes the expression pattern of circRNAs in Nile tilapia muscle by high-throughput sequencing and their regulatory networks with miRNA and mRNA as ceRNAs.

Systematical analysis of circRNA transcriptome in Nile tilapia enabled us to detect specific features and relative abundances of different circRNAs, and RT-qPCR validation has confirmed their expression (Fig. 9D). We found a total of 2949 novel circRNAs in slow-and fast-growing full-sib individuals. These results agree with the scope and abundance of circRNAs that have been identified in fish. Nedoluzhko et al. [23] previously reported the presence of 446–928 circRNAs in white muscle tissue of wild and first-generation domesticated Nile tilapia. In a different study, Liu. et al. [21] have identified 445 circRNA in blunt snout bream muscle. When comparing the expression profile of RNAs between slow and fast-growing Nile tilapia groups, we found significant changes in circRNA expression levels accompanied by the size of the fish. A total of 4 differentially expressed circRNAs were identified between fast-growing and slow-growing groups. Of these, two circRNAs were up-regulated, and two were down-regulated. Circ_NC_031972.2:9033630–9,035,959 (CircMef2C), located in LG7, is one of the down-regulated circRNAs in SM group. It is derived from the combining exon and intron region of the *myocyte-specific enhancer factor 2C-like (mef2c)* gene. The host genes of circRNAs are an important source for understanding the biological function of circRNAs. Ashwal-Fluss et al. [24] described that the relationship between circRNAs and their host genes helps to predict the role of circRNAs in organisms. *mef2c* is known to play a crucial role in sarcomeric gene expression, fiber type control, and regulation of metabolism, thus controlling overall body size [25]. Down-regulation of circMef2c in the slow-growing male group suggests its involvement in Nile tilapia muscle growth. Several studies in livestock animals showed that circRNA produced from muscle-associated genes significantly influences muscle development. For example, circRNA produced from the *huwe1* gene, responsible for ubiquitination and proteasomal degradation of *myoD*, promotes proliferation and differentiation of myoblasts, inhibiting apoptosis [26]. Likewise, circMYBPC1 produced from the *mybpc1* gene promotes the differentiation of myoblasts and skeletal muscle regeneration [27]. In addition, a significant number of mRNAs had a similar trend of expression compared to circRNA expression in the slow-growing group but an opposite pattern from miRNA, which suggests the possibility of ceRNA network in muscle growth of Nile tilapia.

Using high-throughput RNA-seq analysis, we identified 1947 DE mRNAs, including 1002 up-regulated mRNAs and 945 down-regulated mRNAs in the slow-growing group. Among these down-regulated mRNAs, a total of 52 mRNAs, including *mapk15, igfbp2, creb5, adamts16, tnfsf12, fgf16, tgfbi, capn9, myf5, fgf13, fgf14, myod1, map2k4, map3k2, cdkn3, asb15* are associated with muscle growth which suggests a massive change in muscle development pattern at molecular levels. A recent approach to finding molecular differences in muscle growth of Chinese perch (*Siniperca chuatsi*) and large-scale loach (*Paramisgurnus dabryanus*) reported 8942 and 320 DE mRNAs between large and small individuals, respectively [28,29]. Our results are in line with these findings that describe the significant impact of *myf5, igf, and igfbp* in the accumulation of muscle mass [30,31].

GO term annotation of differentially expressed mRNAs were assembled into glycerophospholipid metabolic process, muscle fiber development, striated muscle cell development, spliceosomal complex, mRNA splicing via spliceosome, spliceosomal tri-snRNP complex, spliceosomal snRNP complex, autophagy, process utilizing autophagic mechanism. Furthermore, in KEGG pathway enrichment analysis, we found that DE-mRNAs were associated with spliceosome, mitophagy, and ribosome proteasome. Mitochondria are essential energy-providing organelles that are required in the maintenance of muscle tissue, and mitophagy is a necessary pathway to maintain mitochondrial quality in muscle homeostasis [32,33]. The genes *bnip3, pgam5, park7, and optn* are significantly enriched in this pathway; *park7* can activate the transcription of autophagy genes induced by endoplasmic reticulum stress [34]. Besides, *bnip3* - a member of the *bcl-2* family of cell death-regulating factors - mediates mPTP opening, mitochondrial potential, mitochondrial respiratory collapse, and ATP shortage of mitochondria from multiple cells [35]. Thus, impaired mitophagy suggests selective autophagy of damaged or unnecessary mitochondria could lead to loss of muscle mass and function in adult fish.

The spliceosome is a large RNP complex comprising U1, U2, U4/6, and U5 small nuclear RNPs (snRNPs) and several hundred protein factors. Core protein components of the spliceosome contribute to splicing regulation by modulating splice site choice. The spliceosome recognizes splicing signals located both at exon-intron boundaries and numerous *cis-*regulatory sequences that act as splicing enhancers or silencers [36]. It has been shown that spliceosomal complex-dependent alternative splicing produces multiple mRNA variants in muscle [28]. The spliceo-some is also involved in circRNA production in addition to its role in constitutive and alternative linear RNA splicing [37]. The requirement of the spliceosome in circRNA formation has been investigated by inhibiting the canonical spliceosome using a pre-mRNA splicing inhibitor [38]. The authors showed that isoginkgetin, which blocks spliceo-some assembly at the stage of U4/U5/U6-tri-snRNP, drastically reduces the turnover of natural circRNAs in HeLa cells. In addition, Liang et al. [39] have found that circular RNAs become the preferred output when core spliceosome or transcription termination factors are depleted from cells. Therefore, the change in the expression of *snu114, sf3a, and u1a* for spliceosome assembly may likely result in changing the levels of many-core spliceosomal components, which in turn will profoundly impact the expression of circRNA splice variants in Nile tilapia muscle.

Recent studies have demonstrated that both mRNA and circRNAs act in the ceRNA network by competing for shared miRNAs. It is well known that circRNAs regulate miRNA activity through binding with miRNA-responsive elements [16]. miRNAs have similar regulatory functions and serve a decisive role in growth by inhibiting targeted mRNA. For this reason, several studies have evaluated mRNA, miRNA, and circRNA co-regulatory relationships during muscle development. For instance, circLMO7 in bovine muscle can competitively absorb miR-378a-3p, which targets the *hdac4* gene, thus promoting muscle cell proliferation and inhibiting muscle cell differentiation [40]. Wang et al. [41] reported the overexpression of circTTN in bovine skeletal muscle induces an inhibitory effect on miR-432 by complementary binding. Inhibition of miR-432 activates the IGF-II/phosphatidylinositol 3-kinase (PI3K)/AKT signal pathway, which promotes the proliferation and differentiation of bovine myoblasts.

We have identified in silico miRNAs that show complementary binding to circRNAs and mRNAs that are possibly involved in the ceRNA network. circMef2c exhibited target correlation with oni-miR-34, oni-miR-130b-5p, and oni-miR-202 expression that, in turn, correlate with their targeted genes under the same growth-associated trajectory. Oni-miR-202, oni-miR-130b-5p, and oni-miR-34 were significantly expressed in slow-growing group comparing with fast-growing. Interestingly, many miRNAs, such as miR-202 and miR-34, have been previously identified during skeletal muscle development of Nile tilapia [42,43]. Moreover, muscle-specific miRNA can target muscle-specific PCMs to mediate skeletal muscle cell proliferation and differentiation. Oni-miR-34 and miR-202 are highly expressed in slow-growing fish muscle and predicted to target down-regulated *igfbp2* and *myod1,* respectively. It is assumed that *igfbp-2* in many fish species positively regulates the *igf-i* activity through binding to their receptors [44]. *igf-i* can activate PI3-kinase and MAPK signalling pathways, which regulate myoblast proliferation and differentiation [45]. In salmonid, *igfbp-2* follows a directional regulation of plasma *igf-i* abundance, which suggests their co-regulation to achieve a specific free-to-bound *igf* ratio that promotes an appropriate physiological response [46,47]. Similarly, *fgf14* has been reported in Nile tilapia muscle which plays an essential gene for somatic growth. A recent study on *fgf14* showed that the overexpression of this gene influences cell proliferation and induces cell apoptosis via mediating PI3K/AKT/mTOR pathway [48]. Currently, it is unknown whether oni-miR-130b-5p is involved in proliferation, differentiation or autophagy in skeletal muscle. However, oni-miR-130b-5p was expressed at a higher level in slow-growing fish compared to the fast-growing group. It has been shown that overexpression of miR-130b dramatically suppressed both in vitro and in vivo proliferation of cells, which could be attributed to influence apoptosis and cell cycle arrest [49]. In addition, the bioinformatic analysis showed that oni-miR-130b-5p (homologous to mmu-miR-130b-5p) target downregulated *myod1,* and the inhibition of *myod1* (*myod1*, usually referred to as *myod*) in cells negatively regulate the proliferation of self-renewing myogenic stem cell population thus, reducing post-natal growth [50].

In summary, this is the first study to provide an overview of circRNA, miRNA, and mRNA co-expression in Nile tilapia muscle tissues. Hundreds of circRNAs, miRNAs, and mRNA were identified; several showed significant differences in expression when comparing fast and slow-growing fish. We established a multiple ceRNA network, including circMef2c-oni-miR-34-igfbp2, circMef2c-oni-miR-130b-5p-myod1, and circMef2c-oni-miR-202-fgf14, which could serve an important role in muscle gain throughout ontogeny. These results point to a new direction for understanding muscle development and exploring growth biomarkers in teleost species.

## 4 Materials and methods

### 4.1 Ethical approval

Research involving live fish was approved by the ethical committee of Nord University (Bodø, Norway) and performed following the regulation and instructions of the Norwegian Animal Research Authority (FOTS ID 1042). All procedures were conducted according to the EU Directive 2010/63 on the use of animals for scientific purposes.

### 4.2 Sample collection

Fertilized eggs were captured using traditional fishing nets and traps at the river Nile in Luxor (Egypt) and transferred to Nord University as the base population (F0) for our Nile tilapia breeding program. The fish were kept in a freshwater recirculating aquaculture system maintained at the temperature = 28 °C, pH = 7.6 [9,10]. The photoperiod was adjusted at 11:13 h dark:light. All fish were fed ad libitum with 0.15–0.8 mm Amber Neptun pellets (Skretting, Norway). They were maintained for 3 generations and selected for improved growth [8]. The samples for this study were collected from 9-month-old F3 fish reared in common garden to minimize environmental effects. Males were selected because they grow faster than females and are preferred for fish farming. Six slow- (SM) and six fast-growing (BM) full-sib male Nile tilapia were randomly selected. Prior to sampling, fish were euthanatized with clove oil (Sigma Aldrich, USA) before sample collection using a 1:10 mix of 15 mL clove oil with 95% ethanol diluted in 10 L of system water. Fish gender was confirmed by checking the genital papilla. Fast (white) muscle was collected by careful dissection from the left dorsal quadrant and snap-frozen in liquid nitrogen. Muscle samples were stored at 80 °C until total RNA extraction.

### 4.3 Total RNA extraction and quality control

The frozen fast muscle samples were briefly homogenized using beads (Qiagen GmbH, Germany) and TRI reagent (Zymo Research, USA) at 6500 rpm for 3 × 20s in a Precellys 24 homogenizer (Bertin Instruments, France). Total RNA was extracted from the homogenized tissue using the Direct-zol RNA kit (Zymo Research, USA) according to the manufacturer’s instructions. RNA purity was assessed on a Nano-Drop ND-1000 spectrophotometer (Thermofisher Scientific, USA) with the criteria of A260/280 ≥ 1.8 and A260/A230 ≥ 2.0. Afterwards, the concentration of RNA was determined using Qubit RNA Assay Kit in Qubit 3.0 Fluorometer (Thermofisher Scientific, USA), and the RNA integrity was evaluated using TapeStation 2200 (Agilent Technologies, USA). Only RNA samples with an RNA integrity number (RIN) ≥ 7 were used for library preparation and sequencing.

### 4.4 mRNA, miRNA and circRNA library preparation

All samples were processed for mRNA, miRNA and circRNAs library construction. An amount of 200 ng total RNA per sample was used for mRNA sequencing library construction. The libraries were constructed using the NEBNext Ultra II Directional RNA Library Prep (NEB, USA) with the NEBNext Poly(A) mRNA Magnetic Isolation Module (NEB, USA). After poly(A) enrichment, mRNA was fragmented to 100–200 nucleotide (nt) length for first and second-strand cDNA synthesis. The cDNA was purified, end-repaired and used for adaptor ligation followed by multiplexing using NEBNext Multiplex Oligos (NEB, USA). Nine PCR cycles were used to amplify the libraries; cDNA library purification was carried out with AMPure XP beads (Beckman Coulter Inc., USA).

Small RNA multiplexed libraries were prepared from 200 ng RNA and using NEXTFLEX® Small RNA-Seq Kit v2 (PerkinElmer, USA). Adaptor ligation, PCR, clean up and size selection were performed according to the manufacturer’s instructions with minor modifications, as reported [51].

For circRNA library preparation, 1 μg of total RNA for each sample was used as input. Total RNA was treated with RNase R (Lucigen, USA) for one hour at 37 °C to digest all linear RNA and then purified with Agencourt RNAClean XP beads (Beckman Coulter Inc., USA). Subsequently, the NEBNext rRNA Depletion Kit v2 (Human/Mouse/Rat) with RNA Sample Purification Beads (NEB, USA) was used to ensure the complete removal of ribosomal RNA (rRNA). At the end of this procedure, RNA samples contain mainly circular RNA fragments. CircRNA multiplexed libraries were prepared using the NEBNext Ultra II RNA Library Prep Kit (NEB, USA). In brief, the purified RNA samples were used for the first-strand and second-strand cDNA synthesis and adaptor ligation. A unique barcode was tagged in each 12 samples and amplified with 16 PCR cycles.

The quality and quantity of individual mRNA, miRNA, and circRNA libraries were assessed using Agilent High Sensitivity D1000 ScreenTape assay in Agilent 2200 TapeStation (Agilent Technologies, USA). The multiplexed circRNAs, miRNA and mRNA libraries, were then pooled separately at equimolar concentrations and loaded on the flow cells at 1.5 pM. Sequencing was performed on Illumina NextSeq 500 platform (Illumina, USA) with the NextSeq 500 High Output Kit for paired-end (75 bp for circRNA and 150 bp for mRNA) and single-end (75 bp for miRNA) reads at the Nord University genomics facility (Bodø, Norway).

### 4.5 Bioinformatic analyses

#### 4.5.1 Quality control and mapping

The quality of the raw DNA reads (both Q20 and Q30) and GC percentage was assessed with fastqc (v0.11.5). From circRNA and mRNA data, the reads containing adapter, poly-N and poor quality reads *(<* Q20) were removed using fastp v0.19.10 [52], and further downstream analyses were performed only with high-quality clean data. The reference genome (NCBI accession: GCF_001858045.2_O_niloticus_UMD_NMBU) [53] and respective annotation file were downloaded from the National Center for Biotechnology Information (https://www.ncbi.nlm.nih.gov/). To identify circRNAs, clean reads were mapped to the Nile tilapia reference genome using BWA (v0.7.17) with the parameters: -T 19 -t 8 [54]. The resulting mapped reads from each sample were used as candidates for back-spliced junction detection in CIRI2 v2.0.6 with the -T 4 parameter [55].

In the case of mRNA, cleaned data were mapped to the same reference genome with HISAT2 (v2.2.1) using the default parameters [56]. Next, mapped reads were annotated to reference transcriptome with featureCounts [57] to obtain read count for each gene; subsequently, all samples were concatenated into one count matrix.

For miRNA analysis, adapter sequences and low-quality (Phred quality score 〈20) sequences were removed using Cutadapt v1.12 [58]. Clean reads outside the range of 15–35 nt were excluded. All sequences were aggregated based on the group, and representative reads were then mapped to the same Nile tilapia reference genome through a custom perl script (https://github.com/Qirui4172/Salmon_RNA-seq_miRNA-seq_integrative_analysis). With mirDeep2 v0.1.3 [59], the mapped small RNA tags were used to identify known mature Nile tilapia miRNAs with a single mismatch using miRBase version 22 (http://www.mirbase.org). Customized perl scripts were used to obtain the miRNA counts and concatenate them into a combined count matrix (https://github.com/Qirui4172/Salmon_adherent_cells_RNAseq_miRNAseq_integrative_analysis).

#### 4.5.2 Splice variant analysis

Quantification of gene expression levels from RNA-seq reads also depends on accurately identifying isoforms (splice variants) of a given gene produced in each read. To identify splice variants, all the clean mRNA reads obtained in this study were mapped to the Nile tilapia reference genome using Tophat v1.3.2 [60], guided by its corresponding annotation (GTF) file. Mapped reads were then assembled with the Cufflinks program v1.3.0 [61] using the default parameters. Next, differentially expressed isoforms (adjusted *p-value* ≤ 0.05) were identified by comparing BM and SM groups using Cuffdiff (part of the Cufflinks package). The Cuffdiff program also reports genes showing differential levels of alternatively spliced transcripts.

#### 4.5.3 Differential expression of mRNAs, miRNAs and circRNAs

The expression analysis of circRNAs was conducted with circMeta, which uses junction reads without considering the host gene [62]. It performs the deviance goodness of fit test for each circRNA. circRNAs with |Log_2_fold change| ≥ 1 and p adjusted value ≤0.05 were considered significantly differentially expressed. Finally, host genes of differentially expressed circRNAs were predicted using CircParser [23].

Differential expression of mRNA and mature miRNAs between the BM and SM groups were assessed with the DESeq2 algorithm [63], which uses normalized counts. To be considered differentially expressed, mRNA and miRNA transcripts displayed a minimum |Log_2-_fold change| ≥ 1, as well as an adjusted *p-value* ≤ 0.05 (Benjamini–Hochberg multiple test correction method).

#### 4.5.4 Functional analysis

Gene ontology (GO) and Kyoto Encyclopedia of Genes and Genomes (KEGG) enrichment analyses were performed with significantly differentially expressed mRNA genes (adjusted *p-value* ≤ 0.05). Gene IDs were extracted and annotated in GO and KEGG databases using clusterProfiler [64]. A corrected *p-value* (q-value) ≤ 0.05 (Benjamini–Hochberg multiple test correction) was used as a threshold level of significance for the functional enrichment. Visualization of GO terms was generated using the GOplot R package [65].

#### 4.5.5 Construction of ceRNA network

To explore the co-expression of circRNA, miRNA and mRNA, circRNA–miRNA–mRNA (ceRNA) networks were constructed based on possible functional relationships between DE-circRNAs, DE-miRNAs, and DE-mRNAs. Interaction between DE-circRNA and its targeted DE-miRNA was predicted using Miranda v3.3a software [66] with a threshold of energy score ΔG = 12 kcal/mol and a paring score S = 120. The targets of the DE miRNAs were predicted with the parameters ΔG = 20 kcal/mol and S = 140. Finally, circRNA–miRNA–mRNA networks were constructed and visualized using Cytoscape 3.8.2 [67].

#### 4.5.6 RT-PCR, Sanger sequencing, and quantitative PCR

Primers and PCR conditions for circRNA validation (Table 3) were developed using a custom docker-based workflow, which includes the Primer3 tool [68]. The same total RNA samples used for the RNA library preparation were used to validate the presence of differentially expressed circRNAs by PCR. We selected circMef2c produced from the growth-associated gene *mef2c* for back-splice junction validation. Complementary DNA (cDNA) was synthesized from 200 ng total RNA using the QuantiTect Reverse Transcription Kit (Qiagen, Germany). The obtained cDNA was further diluted five times with nuclease-free water and used as the PCR template. Divergent primers were expected to amplify the circRNAs fragment near the junction point. PCR amplification was performed with 40 cycles using AmpliTaq Gold DNA polymerase (Thermo Fisher Scientific, USA), and the PCR products were visualized by 2% (*w*/*v*) agarose gel electrophoresis. Subsequently, the amplified PCR product was used to confirm the back-splice junction sequences by Sanger sequencing. Quantitative qPCR analysis of differentially expressed circRNAs was performed using divergent primers and SYBR green in LightCycler® 96 Real-Time PCR System (Roche Holding AG, Switzerland). The relative expression levels of the target circRNAs were calculated using the ΔΔCT method considering *actin beta* and *elongation factor 1-alpha* as reference genes.

Similarly, the reliability of mRNA and miRNA sequencing data was confirmed by qPCR. Using the NCBI Primer-BLAST tool, we designed the primers for the chosen mRNAs. The primers were then examined by NetPrimer for secondary structures such as hairpins, repetitions, self-dimers, and cross-dimers (Premier Biosoft, Palo Alto, USA). Supplementary table S5 contains a list of selected mRNA primers and their annealing temperature. A Bio-Rad CFX96 real-time PCR machine (Bio-Rad Laboratories, Hercules, CA, USA) was used for qPCR amplification under the following conditions: an initial enzyme activation/cDNA denaturation step at 95 °C for 1 min, followed by 45 cycles at 95 °C for 15 s, 58–61 °C (specific to each primer; Supplementary Table S5) for 15 s and 72 °C for 15 s, with a final standard dissociation protocol to obtain the melting profiles. Data were acquired and analyzed using the CFX Manager software (Bio-Rad). The relative expression levels of the target circRNA were calculated using the delta-CT method considering *actin beta* and *elongation factor 1-alpha* as reference genes.

For validation of the small RNA-seq data, cDNA was synthesized from 200 ng total RNA using the miRCURY LNA RT Kit (Qiagen, Germany), according to the manufacturer’s instructions. The obtained cDNA was further diluted 20 times with nuclease-free water and used as qPCR template. Relative expression of 8 DEmiRNAs (Table S6 in Supplementary material) was analyzed using the miRCURY LNA SYBR Green PCR kit and appropriate miRCURY LNA miRNA PCR assay primers (both QIAGEN, Hilden, Germany), following the manufacturer’s instructions. Thermocycling was conducted on a CFX96 Touch Real-Time PCR detection system (BioRad, Munich, Germany). The qPCR reactions were set under the following conditions: initial heat activation at 95 °C for 2 min, 40 cycles at 95 °C for 10 s, and 56 °C for 60 s followed by a melt curve analysis. qRT-PCR reactions were performed in triplicates of 6 biological replicates in each group. Data were acquired and analyzed using the CFX Manager software. The relative expression levels of the target miRNAs were calculated using the ΔΔCT method considering oni-miR-10c and oni-miR-26b as reference genes.

## Supplementary Material

Supplementary Material

## Figures and Tables

**Table 1:** Summary of RNA sequencing and mapping statistics for combined Nile tilapia muscle datasets.

Library type	Total reads	Clean reads	Clean reads >Q20 (%)	Mapping rate (%)	11	Singletons (%)
circRNA	660,513,692	623,923,612	94.4	91.3	92.7	1.2
mRNA	964,592,220	938,496,548	97.2	93.9	70.5	2.5
miRNA	319,445,174	276,419,289	86.5	58.9	–	–

**Table 2:** Differentially expressed circRNAs between slow- (SM) and fast-growing (BM) Nile tilapia fast muscle, their location, and host genes.

CircRNA ID	CircRNA name	Genomic location	Expression Log2FC	Structure	Host gene
NC_031978.2:30702661–30,704,116	CircPiezo1	LG13	3.55	Exonic	*piezo type mechanosensitive ion channel component 1*
NC_031976.2:36016595–36,018,688	CircLamin A/C	LG11	− 4.86	Exonic	*lamin A/C*
NC_031972.2:9033630–9,035,959	CircMef2c	LG7	−1.24	Exonic	*myocyte-specific enhancer factor 2C-like (mef2c)*
NC_031972.2:48086230–48,086,927	CircTnnt	LG7	4.76	Antisense	*troponin T, cardiac muscle isoforms*

**Table 3:** CircRNAs and primer sets used for PCR validation.

CircRNA	Primer sequence (5′–3')	Annealing temperature (°C)	Amplicon size (bp)	Efficiency (%)
circMef2c	Forward: GCGACTGTGAGATTGCCCT	58	364	89
	Reverse: ACTGGAAGCACACTGTGAAA			
CircLaminA/C	Forward: CTATGAGACGGAGCTGGCG	58	270	86
	Reverse: CTTCTGACTGCAGCCTGCT			
CircPiezo1	Forward: AACAGTCGTCCTCTGCAGC	58	302	105
	Reverse: ATCAGCCCATCAGCGACAG			
CircTnnt	Forward: GATCCAGTGGGGGAGCTGT	58	351	94
	Reverse: GCTTGACAGCATACCCCCA			

## Data Availability

The Nile tilapia mRNA, microRNA, and circRNA sequencing data are available at NCBI Bioproject with the accession number PRJNA825740.
