# Supplementary material for: The novel circular RNA CircMef2c is positively associated with muscle growth in Nile tilapia
Source: Genomics. Author manuscript; Available in PMC 2023 May 1. (PMC7614353; doi:10.1016/j.ygeno.2023.110598)
Supplement: Supplementary Material [file EMS172244-supplement-Supplementary_Material.pdf]

Supplementary Material to “The novel circular RNA CircMef2c is positively associated with muscle growth in Nile tilapia”

Golam Rbbani[1], Artem Nedoluzhko[1,2], Prabhugouda Siriyappagouder[1], Fedor Sharko[3], Jorge Galindo-Villegas[1], Joost A. M. Raeymaekers[1], Rajesh Joshi[4], Jorge M.O. Fernandes[1,*]

[1] Genomics Division, Faculty of Biosciences and Aquaculture, Nord University, 8049 Bodø, Norway

[2] Paleogenomics laboratory, European University at Saint Petersburg, 191187 Saint-Petersburg, Russia

[3] LLC ELGENE 109029 Moscow, Russia

[4] GenoMar Genetics AS, 0252 Oslo, Norway

* Corresponding author

Email: jorge.m.fernandes@nord.no

Supplementary Tables

Table S1. Summary of RNA sequencing and mapping statistics for mRNAs.

Sample*	Total reads	Clean reads	Mapping rate (%)
BM1	40,562,308	39,177,794	96.5
BM2	41,444,990	40,100,728	97.1
BM3	38,188,772	37,375,984	95.6
BM4	43,772,452	42,706,866	96.2
BM5	38,741,342	37,698,024	93.5
BM6	40,895,484	39,715,586	94.0
SM1	38,048,096	37,169,182	89.8
SM2	45,507,044	44,125,214	94.4
SM3	39,083,626	38,182,532	95.7
SM4	36,732,430	35,831,084	87.6
SM5	38,424,082	37,449,694	92.3
SM6	40,895,484	39,715,586	95.2

*BM = Big (i.e. fast-growing) male; SM = Small (i.e. slow-growing) male

Table S2. Summary of RNA sequencing and mapping statistics for miRNAs.

Sample*	Total reads	Clean reads	Mapping rate %
BM1	28,552,142	25,306,282	67.0
BM2	23,195,202	18,987,254	53.3
BM3	25,754,609	22,156,914	60.0
BM4	27,684,174	23,697,152	57.7
BM5	22,318,164	18,291,113	41.0
BM6	23,171,430	19,163,426	44.0
SM2	30,988,212	32,282,553	71.5
SM3	40,233,691	32,282,553	62.1
SM4	41,564,146	36,597,050	69.7
SM5	27,906,526	23,170,198	62.4
SM6	28,076,878	24,484,794	59.8

*BM = Big (i.e. fast-growing) male; SM = Small (i.e. slow-growing) male

N.B.: One of the samples had a considerably low sequencing output compared to the rest. Thus, it was excluded from further analysis.

Table S3. Summary of RNA sequencing and mapping statistics for circRNAs.

Sample*	Raw reads	Clean reads	Mapping rate (%)
BM1	36,143,810	34,428,113	92.4
BM2	37,457,612	35,387,692	91.1
BM4	27,340,761	25,917,832	91.2
BM5	31,848,151	30,192,774	89.4
BM6	34,529,931	32,917,529	91.8
SM1	34,586,325	32,767,462	91.5
SM2	31,282,889	29,714,527	91.0
SM4	32,095,956	28,715,316	90.1
SM5	32,464,650	30,730,915	90.9
SM6	32,506,761	31,189,646	92.0

*BM = Big (i.e. fast-growing) male; SM = Small (i.e. slow-growing) male

N.B: The BM3 library was excluded from the downstream analysis, since it had a low sequencing output.

Table S4. List of differentially expressed genes between fast and slow-growing males

Gene symbol	Base mean	Log_2Fold Change	pvalue	padj
LOC109203007	1540.13164	12.452083	1.97E-09	2.71E-07
LOC109203009	1588.673516	12.04025522	2.85E-08	2.61E-06
LOC109203003	874.4890294	11.9779253	3.21E-07	1.90E-05
LOC100692254	1380.858897	11.83922254	8.96E-08	6.88E-06
LOC109203010	1331.963486	11.73778143	1.54E-09	2.22E-07
LOC109203022	401.4605274	10.36729364	7.58E-07	3.85E-05
LOC109194430	435.1858599	10.17024753	1.63E-06	7.16E-05
LOC109201864	1744.757583	9.000954865	1.01E-07	7.38E-06
LOC109203027	181.3703123	8.543170903	1.08E-05	0.000315568
LOC100534448	900.3024224	8.101802116	3.05E-07	1.84E-05
LOC100700083	4018.181568	7.884583327	7.17E-08	5.75E-06
LOC106098112	43.621092	6.847147184	0.003650907	0.026897761
LOC100693662	8.017611811	5.145833773	1.82E-05	0.000469627
LOC100692910	1100.77189	4.986609555	0.002249182	0.018664191
chrna1	27.63393114	4.944592234	3.28E-07	1.94E-05
LOC102077117	16.01905146	4.783298783	3.91E-06	0.0001446
lingo2	6.289533143	4.760791661	6.76E-05	0.001292336
LOC100696639	56.52686856	4.570469361	1.03E-10	2.40E-08
LOC100708285	33.85181304	4.519298586	7.58E-09	8.54E-07
LOC100691691	5.2248999	4.516994577	0.000187261	0.00284613
LOC109195973	6.420486409	4.338224499	0.000723373	0.008066022
LOC100694066	34.35950905	4.26674251	0.004566769	0.031474638
LOC100707925	14.28781272	4.247693001	1.14E-07	8.13E-06
LOC112843841	29.96568266	4.195675776	6.46E-06	0.000213307
LOC100704606	5239.765678	4.136237718	5.20E-05	0.001037017
LOC100690112	5925.480671	4.088577745	0.000149736	0.002411867
LOC100703944	8.019615196	4.074513684	0.001591336	0.014429146
LOC100705436	270.5196033	4.018580978	3.21E-12	1.15E-09
spire2	88.98585469	3.971752465	1.54E-05	0.000415927
ppp1r3b	7.218637713	3.886615213	0.00718305	0.04455073
LOC102079795	311.2352287	3.837056327	5.36E-20	1.66E-16
LOC100701127	794.9865092	3.800125495	1.81E-40	3.37E-36
map7	27.40785103	3.648273566	0.000110245	0.001886959
LOC109202485	136.3783833	3.627384864	0.000158473	0.002500587
tbtbp	30.34158969	3.595258293	3.99E-07	2.27E-05
LOC109195527	7.18384967	3.576671797	0.008041411	0.048232898
LOC100694764	24.01075177	3.566798511	0.000277594	0.003847806
LOC100705876	9.906403268	3.52265385	6.74E-05	0.0012905
LOC100699707	12.96003162	3.502056586	9.10E-06	0.000281828
LOC102080329	18.32540636	3.482132309	0.000249922	0.003569994
LOC100710332	8.649505595	3.475012068	0.001484761	0.013703446
LOC100699475	7.340549983	3.474891794	2.29E-05	0.00055713
LOC100704234	6.134034858	3.473341964	0.001962323	0.016863455
LOC100696194	139.9236339	3.466612986	4.95E-10	9.21E-08
LOC100709688	54.97108674	3.466349255	7.78E-07	3.93E-05

LOC100708428	3000.589702	3.460787951	4.94E-06	0.00017535
hspbp1	585.9285797	3.457658821	3.43E-09	4.33E-07
uchl1	1194.932364	3.446285384	6.26E-05	0.001208393
dnaaf2	24.15934401	3.38743893	1.07E-05	0.000314023
LOC100696738	64.1196082	3.330114833	6.65E-06	0.000219161
LOC106098855	9.077488603	3.310468846	0.000428915	0.005368799
LOC100700186	3680.928906	3.302737939	3.41E-09	4.33E-07
LOC100703193	16.98328111	3.247379194	0.000715215	0.007989437
ptger3	25.13351608	3.17144587	7.77E-05	0.001446103
LOC100693491	124.937387	3.16155506	0.001577404	0.01433271
LOC100695865	170.7952449	3.143600785	3.52E-07	2.05E-05
LOC109201771	6.152525815	3.126687653	0.00183898	0.016086102
LOC100708757	90.55468056	3.12422538	9.44E-08	7.07E-06
ush1c	17.34784002	3.096735765	3.66E-05	0.000775407
LOC100695866	8551.349769	3.095383422	8.26E-14	4.70E-11
LOC102081165	272.656472	3.081641457	5.81E-17	7.20E-14
LOC100699800	7743.361649	3.061193402	4.17E-10	7.98E-08
adgrg2	7.421191391	3.054264224	0.001143852	0.011249697
LOC100696332	43.64292247	3.023010067	0.000375832	0.00486488
LOC106097877	36.00022537	3.020706271	4.70E-05	0.000949845
LOC100690935	48.73610666	2.999933852	0.003088946	0.023757362
LOC100702247	14047.19456	2.99852666	8.06E-08	6.32E-06
LOC100699098	103.6384535	2.990102409	1.45E-06	6.55E-05
tmem79	16.75000665	2.938724209	0.000249594	0.003568809
LOC100698553	310.8938974	2.914482344	8.47E-11	1.99E-08
LOC100707042	9.783209874	2.914237611	0.001614665	0.014562542
LOC100704783	1141.485246	2.881319806	3.47E-07	2.03E-05
LOC100709033	5.541191239	2.876731635	0.001611521	0.014555368
LOC100707199	40.99364915	2.875426327	5.72E-08	4.75E-06
psme4	5439.435964	2.865643346	3.09E-07	1.85E-05
eftud2	545.9293397	2.849164931	0.000218421	0.003225711
LOC100698444	152.1661583	2.843275645	1.72E-09	2.40E-07
slc16a2	267.7077327	2.842769264	4.07E-07	2.30E-05
LOC112844099	16.96835674	2.821642979	0.000107618	0.001857391
LOC112847893	10.8871873	2.802587177	0.000819552	0.008836327
LOC100691388	7.235489233	2.78906528	0.003356163	0.025308056
LOC102082290	21.19963206	2.770936412	0.002541716	0.020443531
LOC102079604	11.10360164	2.769141698	5.03E-05	0.001009991
slc16a6	234.8303227	2.758355047	3.10E-14	2.03E-11
eno2	21.43602578	2.735218938	0.002257607	0.018725355
LOC100702173	6.747517025	2.724334138	0.00027843	0.003856519
LOC112847500	9.409914932	2.702904367	0.000240815	0.003473611
LOC100697636	8794.704043	2.693561358	7.27E-06	0.000235868
LOC112841786	55.41723841	2.683317022	2.57E-07	1.56E-05
LOC100703596	78.61633394	2.663960529	1.30E-07	9.03E-06
LOC112847396	21488.81013	2.65982028	0.000196823	0.002964784
LOC100697774	615.3521717	2.657421831	9.26E-09	1.01E-06
rspo1	9.716579484	2.651456715	0.000625161	0.007186449

LOC112847722	11.17861526	2.635187763	0.000193371	0.002927014
LOC100705062	367.6580237	2.608020989	2.15E-07	1.34E-05
LOC100704832	59.69229805	2.60204097	1.15E-06	5.35E-05
LOC100691603	1099.746258	2.572266625	1.10E-08	1.18E-06
LOC100707217	94.7786427	2.570723251	7.66E-07	3.88E-05
LOC100690242	2345.218305	2.567750397	5.48E-10	9.89E-08
LOC100702118	12.29782146	2.567034974	0.001177264	0.011502593
LOC102080166	17.01514875	2.561743842	0.000335426	0.004459875
LOC100710042	30.57484205	2.559462697	0.0004098	0.005164313
gldc	77.91820587	2.547103043	9.81E-05	0.001720322
LOC100698458	91.41184192	2.527948622	1.52E-07	9.99E-06
LOC100695202	16.17795985	2.527423611	0.000368661	0.004805523
LOC100702076	54.83498509	2.511707028	0.000317728	0.004273463
LOC100701802	39.03739961	2.510752222	0.000729123	0.008110671
LOC100702311	750.2683199	2.510066713	9.81E-08	7.21E-06
pcyt2	1858.65832	2.508040747	4.15E-08	3.67E-06
slc8b1	1509.443051	2.50233373	1.06E-06	5.06E-05
LOC100691238	11.34028713	2.492562037	0.001358535	0.012844578
mastl	107.6575394	2.492457635	2.09E-15	1.85E-12
LOC100709941	306.1452183	2.491262696	3.57E-15	3.01E-12
dnajb9	584.6487477	2.490007967	1.75E-10	3.79E-08
dnajb1	2783.728502	2.486348079	0.000717666	0.008012
LOC100707373	110.5352393	2.48316263	0.000229453	0.003342531
pacrg	18.39926158	2.474946581	1.24E-07	8.66E-06
LOC100710142	204.9812044	2.473740886	4.68E-09	5.72E-07
fitm2	63.03002136	2.467954357	4.15E-07	2.32E-05
LOC100701517	677.1267108	2.467269814	1.52E-05	0.000412901
kiaa1958	14.12616849	2.466899314	7.93E-06	0.000252259
dpp3	618.9923664	2.458259538	9.23E-07	4.49E-05
LOC109194455	164.1393988	2.44556266	2.80E-06	0.000111667
LOC100697678	428.56936	2.437768852	1.13E-08	1.20E-06
wisp2	41.91396219	2.434689411	0.003074589	0.023704052
snrpa	44.10316023	2.431098987	1.77E-05	0.000460032
LOC102080515	80.75932289	2.428561799	4.75E-09	5.77E-07
LOC100711993	65.42856707	2.425111276	0.0013624	0.01287458
LOC100703194	183.0552255	2.424585612	1.37E-14	1.02E-11
LOC102077341	7726.849415	2.420703527	9.24E-06	0.000284881
LOC109194203	21.55699783	2.407943911	0.005603317	0.036947304
tifa	57.68184498	2.40723035	3.23E-05	0.00070508
hspd1	2184.261471	2.39749194	7.27E-09	8.34E-07
LOC100690743	643.3718006	2.38480573	2.24E-13	1.09E-10
armc6	28.62524228	2.382639429	1.45E-05	0.000398534
LOC112847055	28.51731616	2.381080917	0.000968852	0.009980222
LOC102079006	230.6611977	2.378189466	2.07E-05	0.000511387
LOC100698415	24.97214232	2.377889935	0.001305005	0.012478106
comt	523.7288353	2.366855713	1.73E-05	0.000452864
slc2a12	2217.344271	2.357394187	1.85E-07	1.18E-05
LOC102077416	59.10624522	2.351104633	0.000346355	0.004562756

arap2	11.8810583	2.338840551	0.004308287	0.030322773
LOC102078887	33.44886259	2.337463918	0.005152652	0.03454866
bag2	718.8411296	2.337218989	4.77E-05	0.000962277
rcc1	92.40725459	2.327118811	6.27E-05	0.001208393
elfn2	20.02635353	2.326196465	0.000391938	0.005010556
LOC100692968	5230.681602	2.32024773	1.71E-17	2.44E-14
LOC109202234	16.91113012	2.30594683	0.000221742	0.003263449
LOC100695437	7.464292459	2.303970567	0.003655263	0.02691706
mmp9	53.89854004	2.303537087	0.000593463	0.006916174
LOC100699528	453.6026961	2.30282388	1.65E-08	1.66E-06
flcn	1125.084425	2.301287263	6.53E-08	5.32E-06
sqstm1	4233.660595	2.301208668	8.53E-09	9.44E-07
LOC100534396	18.6150484	2.291086049	0.002205395	0.018379997
LOC100707007	77.96967296	2.289692633	0.000374858	0.004859036
rnf181	701.9655213	2.287715231	3.94E-05	0.000822364
manf	910.4394545	2.276183561	4.62E-06	0.000166944
map3k13	84.38655409	2.269006223	3.78E-05	0.000795152
gtpbp2	4973.385889	2.259209915	8.38E-05	0.001532802
tnfrsf21	40.10607983	2.248420895	1.08E-11	3.14E-09
tmem41b	309.1567899	2.244045357	2.59E-14	1.78E-11
fgfbp1	19.58593658	2.231914051	0.003079281	0.023704775
tmem41a	150.2825946	2.230783293	2.04E-12	7.91E-10
pigo	28.18214512	2.196978338	1.33E-05	0.000371609
LOC102077874	13.97889931	2.195485293	0.002190695	0.018317879
LOC112847873	101.6487362	2.189970204	0.000119944	0.002017657
gdpd1	13.33933292	2.179884847	0.001422206	0.013297765
herc4	1406.189366	2.176485661	4.54E-09	5.62E-07
chac2	651.7855054	2.175730407	1.93E-10	4.07E-08
LOC102080145	106.6827352	2.172495012	1.04E-09	1.64E-07
LOC100690871	147.0955199	2.171027141	2.79E-13	1.33E-10
ablim1	74.46887279	2.16077144	0.000390278	0.004999641
dhx30	78.82112439	2.158289585	2.25E-06	9.33E-05
slc30a1	12.21272553	2.153880744	0.000110868	0.00189588
LOC109202457	187.4640168	2.149229081	2.75E-05	0.000635935
LOC100703974	7560.602793	2.138562778	1.18E-07	8.34E-06
relt	12.0775546	2.134699039	0.005557266	0.036734871
LOC100702295	10.9832753	2.12951287	0.003822005	0.02780565
LOC112846112	2254.119382	2.127243993	6.45E-06	0.000213307
nln	15.51084896	2.122537437	0.001135868	0.011216155
LOC100702377	721.9317988	2.122374975	1.99E-05	0.000498094
LOC100701980	85.66200791	2.121316235	3.90E-05	0.000814634
LOC100691615	1605.952844	2.119760511	2.02E-08	1.97E-06
tut1	155.1246427	2.114876141	0.000802957	0.00868677
LOC102075629	598.0250173	2.103528413	8.87E-09	9.76E-07
ccdc171	41.4884388	2.101001855	5.36E-05	0.001057828
smox	1685.074503	2.09905957	1.35E-05	0.000375675
LOC112846916	1052.971977	2.096545667	1.05E-06	5.03E-05
LOC100709258	52.05508044	2.090175085	2.51E-12	9.33E-10

sil1	110.0967711	2.089944657	1.17E-05	0.000334594
LOC100706559	2901.123443	2.088506053	5.95E-07	3.17E-05
LOC100694598	118.7230842	2.087214132	0.000531214	0.006341814
LOC109195675	2469.070976	2.086692223	4.93E-06	0.00017535
cdkn2d	28.80861928	2.072404984	0.0082017	0.048924222
LOC100700475	215.3579547	2.069125756	0.000282324	0.003887285
LOC100711012	27.02769545	2.066759308	0.004677085	0.031997665
gdpgp1	43.38558226	2.06292587	7.41E-07	3.77E-05
nagpa	219.9616867	2.055757932	5.75E-09	6.81E-07
LOC100710977	3022.768225	2.053824894	1.04E-05	0.000310366
acy1	2892.098505	2.053454165	1.43E-06	6.49E-05
LOC100690513	170.5619806	2.05279988	0.000234704	0.003397723
LOC109202137	17.13068782	2.052390179	0.005561104	0.036739815
pde7b	94.9859066	2.049231858	1.31E-05	0.000367124
layn	12.27036184	2.046616742	0.002695699	0.021357038
LOC100709643	382.205829	2.045930181	6.14E-10	1.08E-07
armc7	31.00306048	2.036367876	0.000299239	0.004077892
hdac8	29.11954375	2.026354865	0.000757028	0.008321491
LOC100691220	338.2286321	2.025570446	0.0005353	0.006370282
usp14	2132.38648	2.024986757	3.97E-05	0.000824895
prxl2b	98.38913532	2.021782855	3.75E-06	0.000140631
LOC100702499	2702.174094	2.021146462	2.52E-06	0.000102923
hsp90b1	1666.678616	2.020167089	2.48E-05	0.000592278
anos1	32.80810781	2.017293592	8.62E-08	6.68E-06
LOC102081320	17.36546413	2.017061986	0.005969599	0.038889633
nsun3	44.80073184	2.006008071	1.23E-06	5.66E-05
LOC100690876	816.1891522	1.998367851	4.16E-05	0.000857251
igf2bp2	136.1146031	1.993850139	4.01E-12	1.41E-09
gclm	377.8747982	1.993089034	1.30E-09	1.94E-07
hyou1	441.001282	1.99190727	8.92E-07	4.38E-05
LOC100698905	7282.225018	1.991275395	5.37E-06	0.000185467
gmppb	259.3655773	1.987218926	2.90E-07	1.76E-05
ydjc	68.44135208	1.983620881	6.03E-05	0.001174232
psph	91.42858135	1.981157121	0.000108371	0.001866638
LOC100690902	2493.985907	1.977248388	1.33E-05	0.000371304
LOC109202224	144.0258794	1.974423957	2.60E-06	0.000105323
LOC112847957	84.06565219	1.965235587	0.00033834	0.004485784
polr3d	217.6721711	1.959961404	0.000119866	0.002017657
LOC102076248	112.4828075	1.953503803	0.000151778	0.002426659
LOC100711101	45015.69859	1.953230922	4.11E-05	0.000847912
LOC102080227	1103.425487	1.943480004	0.001084927	0.010859784
LOC100708515	128.7074551	1.940678168	5.92E-07	3.16E-05
ptchd1	94.57973595	1.937323379	0.000838829	0.008992019
mkrn2os	21.53638767	1.935264175	0.000982583	0.010062948
LOC100695535	723.9516332	1.934744519	3.51E-05	0.000751525
alg9	42.25236385	1.933214808	2.51E-05	0.000599096
polq	20.89289521	1.931448074	1.62E-06	7.16E-05
alg2	388.0902161	1.931142121	2.18E-08	2.11E-06

LOC100708763	198.1013589	1.929481914	1.08E-05	0.000315568
LOC109194999	16.51466544	1.921986003	0.000115471	0.00195908
tbl2	146.6484864	1.92010071	8.12E-06	0.000257014
ptdss1	120.525884	1.904662666	5.24E-05	0.001042217
pik3ip1	1313.406745	1.904272227	1.38E-05	0.000379918
hpdl	84.93170842	1.901952618	0.000496155	0.006023855
LOC100699084	112.0545843	1.901523171	1.46E-06	6.55E-05
LOC100703798	154.2455264	1.90068239	0.000602124	0.006990804
LOC100711821	70.04453895	1.900587186	1.94E-06	8.29E-05
LOC100693006	18.54993052	1.899406429	0.00823662	0.049024107
hbp1	1664.404362	1.89712811	1.13E-05	0.000325425
rab12	1951.464345	1.892777273	2.60E-10	5.19E-08
mrps27	201.1039635	1.883125407	0.000200704	0.003013242
sfmbt1	100.4828962	1.882466592	6.97E-05	0.001325162
cdc37l1	3625.701149	1.880503146	1.10E-05	0.000318623
LOC100702498	79.29826235	1.877085085	0.00264697	0.021071469
LOC100701228	28.08807433	1.875863618	0.00160974	0.014555368
LOC109194843	102.5847544	1.870485383	2.23E-07	1.38E-05
LOC112844740	27.12168031	1.865008346	0.003176482	0.024248234
inip	105.8143989	1.863474894	1.89E-07	1.20E-05
LOC100704414	3263.819852	1.861858008	3.59E-07	2.07E-05
LOC106098022	683.2296748	1.858530206	3.58E-05	0.000763774
LOC100708241	37500.43915	1.857894991	9.55E-06	0.000290639
pir	76.71764665	1.857598013	4.59E-05	0.00093038
ccpg1	6238.635549	1.857551444	0.000169402	0.002637226
gpt	13082.58786	1.853431947	2.58E-05	0.000611461
ppih	48.87578153	1.851825318	3.65E-05	0.000774722
ubald2	109.2620896	1.851249544	3.88E-05	0.000811814
grhpr	29.552603	1.847879016	0.000616202	0.007114268
prmt5	112.7990873	1.83448921	3.04E-05	0.000682645
mmaa	25.68953401	1.826991912	0.001395621	0.013107942
LOC100703678	166.6977112	1.826554743	6.20E-06	0.000207882
aacs	24.1174115	1.82646228	0.000691618	0.00777348
znf385c	22.12859489	1.826364671	0.000442771	0.005508858
LOC100711193	288.3828086	1.821215511	3.20E-05	0.000702365
fig4	90.72937923	1.819201034	2.34E-05	0.000564717
hmbs	44.83492144	1.818241372	0.000294061	0.004019118
map3k5	1244.877173	1.815494087	1.67E-06	7.35E-05
psmd14	2841.788264	1.808510354	2.80E-05	0.000641819
LOC100693753	719.3436426	1.808402682	7.58E-05	0.001413229
LOC109195634	9.883863238	1.804213001	0.003539088	0.026282286
adgrv1	10.54472777	1.804032435	0.007574385	0.046252521
LOC100700443	135.450665	1.803616235	0.004537382	0.031306926
mpi	315.6635928	1.80231623	2.76E-08	2.55E-06
brat1	20.05418919	1.800798464	0.003398262	0.025552952
LOC102079432	40.47382672	1.795008956	0.000484761	0.00590375
LOC102075954	15.48938785	1.794343185	0.003925114	0.028322985
slc26a5	225.4398176	1.786530065	5.72E-09	6.81E-07

psmd1	4135.459577	1.786462088	1.35E-05	0.000375675
enkd1	508.0850167	1.78466927	0.000129119	0.002135295
eva1a	1494.31347	1.782552127	3.37E-05	0.000728634
atad3a	650.9262017	1.780969208	0.000106323	0.001840169
sh2b2	1306.017328	1.776848557	5.31E-05	0.001051416
pgp	293.2189145	1.771132275	1.65E-05	0.000437647
parp2	133.1550618	1.76551465	0.000119556	0.002014776
mrpl19	2930.924094	1.763920306	3.49E-05	0.000748804
agtrap	79.58771144	1.763377301	1.20E-06	5.53E-05
LOC102079900	22.23242334	1.761541727	0.001428054	0.013318953
stam	272.6925993	1.757983721	3.81E-08	3.40E-06
LOC112846891	182.486332	1.756912967	1.39E-10	3.15E-08
notum	54.10902915	1.75600796	0.001458182	0.013552343
armc2	333.9438023	1.754913837	5.25E-05	0.001042287
LOC102082314	12.23362438	1.748224166	0.006512079	0.041355151
LOC100705008	87.75463045	1.7468849	1.59E-08	1.62E-06
wdr89	28.58869646	1.74583245	0.00160585	0.014538298
txnrd2	178.0932564	1.742680071	1.08E-05	0.000315568
LOC100701814	164.1405694	1.738627554	5.02E-06	0.000176027
LOC100708450	12.13167029	1.736825376	0.004112849	0.029358541
gss	251.4650366	1.73673508	2.03E-05	0.000503765
ncdn	243.3484234	1.735592865	0.003328048	0.025136833
fbln1	137.2443831	1.735167167	0.000202305	0.00302774
LOC100702411	12.38460026	1.734354607	0.001137927	0.011218742
LOC100702976	1635.477502	1.733746794	8.29E-13	3.76E-10
nfs1	906.0163594	1.732441927	1.14E-13	5.74E-11
LOC100702695	344.5981832	1.731397865	0.000246832	0.003545678
LOC102080285	98.22271038	1.728605932	0.003512571	0.026144383
slc27a4	250.4068292	1.722320032	3.58E-07	2.07E-05
LOC112842916	11.95764558	1.721747607	0.001451615	0.013504814
twf2	15008.56187	1.721243221	2.09E-05	0.000514545
slc3a1	12.02745994	1.720689962	0.004510928	0.031159096
psmc6	2227.844253	1.718839897	8.17E-05	0.001503946
LOC112846758	50.79997981	1.716331205	0.000117135	0.001984784
LOC100701252	1469.108455	1.715284023	1.68E-17	2.44E-14
LOC100711554	196.2669487	1.713760461	9.54E-08	7.07E-06
LOC100709232	158.3087621	1.713604125	3.38E-10	6.66E-08
cptp	20.52673943	1.71346807	0.00121665	0.011760315
phf5a	155.0456556	1.711375803	0.007867597	0.047538444
LOC100689750	9720.910484	1.709368153	9.46E-06	0.000289189
polr3h	202.7833771	1.70345743	1.61E-05	0.000429741
LOC100703336	160.3963546	1.701747493	0.000944985	0.009829534
cth	460.7229518	1.697693078	1.12E-05	0.00032185
LOC100696817	183.1301487	1.692986995	1.04E-05	0.000310366
aptx	68.62702137	1.692819434	9.34E-05	0.00166088
LOC100709516	323.9793068	1.692034493	0.000173583	0.002688794
slc1a5	1181.831826	1.689025817	0.00067615	0.007663582
LOC100702855	2223.711324	1.68838761	3.20E-08	2.89E-06

faxc	82.30259916	1.687072516	0.000329421	0.004405233
nwd2	42.39556789	1.686348446	0.000953274	0.00986725
golga1	334.069723	1.684737064	0.000276445	0.003842703
hhip	18.02602084	1.683520773	0.007796479	0.047267107
vcp	5175.510443	1.682571355	1.29E-07	9.01E-06
LOC100710269	374.2825434	1.680293502	1.10E-06	5.21E-05
ddit3	4306.214768	1.679620599	2.21E-06	9.21E-05
slc15a4	808.0391089	1.678841821	2.82E-05	0.000641819
dtd2	27.76859884	1.67818575	0.003535734	0.026267877
LOC112845994	31.0878857	1.677988691	9.81E-05	0.001720322
slc7a8	9785.252877	1.675998016	0.001049833	0.010582594
irak4	358.5938301	1.675981354	5.65E-11	1.40E-08
acp6	109.2139485	1.669647289	0.000794943	0.008616113
lmbr1	813.543159	1.663578197	6.44E-06	0.000213307
mrnip	278.3970523	1.653084329	5.08E-06	0.000177721
LOC100707516	171.1659813	1.649090015	0.002857404	0.022335331
tmem86a	41.6573535	1.648577343	0.002093466	0.017647777
LOC112843250	435.5207691	1.648392143	0.000953388	0.00986725
dusp22	1578.544818	1.64778224	0.000950064	0.009860297
rbx1	1190.578836	1.647539311	0.000728624	0.008109972
wdr45	826.6992246	1.646049951	4.16E-08	3.67E-06
LOC100691809	74.41770919	1.641448742	0.002559757	0.02054886
eif5	3894.482013	1.64058646	9.58E-05	0.001696515
LOC100699367	192.0934415	1.64052896	0.001583836	0.014382195
LOC102077990	373.7333922	1.639637389	0.000513339	0.006200094
odc1	67.23575046	1.639320287	0.000655925	0.007461643
LOC100690688	176.0317037	1.638384911	8.51E-07	4.23E-05
LOC100703712	344.7973894	1.636925076	4.23E-07	2.36E-05
ybx2	200.7700438	1.635736248	0.000232399	0.003374863
LOC100702179	54.56552815	1.635344267	0.00075755	0.008322303
ppt2	1335.651196	1.632417143	0.00052176	0.006257086
twnk	191.6772615	1.631495933	0.000175231	0.002707561
haus2	251.0304491	1.631484925	1.92E-05	0.000485614
LOC100534433	512.3961233	1.631202496	2.92E-09	3.85E-07
pgam1	251.9693041	1.627422601	4.12E-07	2.32E-05
slc2a4	518.7934636	1.627013049	0.008299827	0.049285045
snx11	323.2975867	1.623675122	6.02E-07	3.20E-05
cdip1	525.7368299	1.623216399	1.05E-05	0.00031212
LOC100702832	498.9500858	1.621851486	2.95E-09	3.86E-07
cnp	1022.346841	1.620548807	8.98E-05	0.001612147
LOC100697229	75.44261713	1.619073236	5.86E-06	0.000199275
LOC100712487	62.48051542	1.618992476	1.19E-05	0.000337792
urm1	45.53718696	1.61821872	4.26E-06	0.000155985
fam98b	77.79657639	1.61786159	0.00027912	0.003863201
LOC100707427	11296.93271	1.61778461	0.000409219	0.005160491
LOC100711812	55.5241947	1.617013128	0.003471554	0.0259675
LOC100689862	93.01184799	1.608190245	0.003738701	0.027392583
plaa	607.9530686	1.605858483	3.64E-05	0.000773855

LOC100707198	30.48324125	1.604612248	0.007091636	0.044042544
galnt13	9.966413006	1.603761707	0.003054527	0.023578717
LOC100707849	48.75692144	1.60213199	0.00013538	0.002223004
cnpy2	45.58746112	1.602044076	0.005161227	0.034573541
LOC100692202	82.70847941	1.600399783	0.007234838	0.044753839
LOC100693161	790.7989389	1.597796506	2.94E-06	0.000116047
xpnpep3	148.8620773	1.597154552	1.15E-05	0.000330676
tram1	94.41127828	1.589813009	1.73E-05	0.000452864
bag3	6411.065828	1.58805889	0.00110778	0.011051432
poc1b	18.57827123	1.587792484	0.003486843	0.026039953
ndor1	118.837074	1.586498519	2.81E-05	0.000641819
wdr6	80.28907435	1.585165985	0.000207301	0.003085119
mfsd9	29.71630079	1.584457305	2.87E-05	0.000651442
creld2	170.7196439	1.583512657	0.001974597	0.016937614
LOC100697795	192.4754473	1.582969835	7.92E-06	0.000252259
LOC112847901	20.49277724	1.582941511	0.004848045	0.032945916
LOC100710353	1660.430923	1.58238245	1.41E-07	9.51E-06
LOC100708654	424.4659553	1.581130258	0.001352447	0.012802175
LOC100707148	219.4674037	1.581034464	2.82E-05	0.000641819
spidr	976.0655267	1.5774726	0.000277336	0.00384711
LOC100696455	369.9853942	1.576355144	8.80E-05	0.001584623
cfap298	356.8756686	1.576307702	3.81E-05	0.000798631
eif4g2	3725.817991	1.575366172	0.001610641	0.014555368
LOC100705961	505.8581377	1.572633551	0.00011014	0.001886898
mroh1	430.261169	1.567278102	0.000375686	0.00486488
cenpo	28.24635184	1.564748535	0.000194962	0.002946304
LOC102079130	17.67064401	1.564607907	0.000877561	0.009310559
ndufaf3	307.9319596	1.564590778	0.000276109	0.003841548
lg8h16orf91	805.3571934	1.563996914	2.57E-05	0.00061134
LOC100691902	33.65765092	1.563899606	0.005268479	0.035138317
tmem147	300.7773572	1.562026509	1.26E-05	0.000355189
nup85	83.86476698	1.561154142	1.92E-05	0.000485614
gdap1l1	20.36803786	1.560994692	0.005445383	0.03612376
gskip	280.0902509	1.560441232	4.83E-06	0.000173588
plekhb2	1104.301314	1.559581242	9.04E-07	4.42E-05
rabggta	486.450669	1.55816715	4.46E-05	0.000908603
LOC100710349	669.1342698	1.558104073	1.92E-05	0.000485614
immp1l	56.11458278	1.558072547	0.001093156	0.010924508
lg7h16orf70	780.8365158	1.557777659	3.87E-05	0.000810291
LOC102083283	16.44148247	1.554932381	0.007876183	0.047548713
pigb	212.9559227	1.550865267	4.94E-06	0.00017535
LOC112846335	13.48906198	1.549539731	0.007863088	0.047538444
LOC100695940	1749.396204	1.548015688	0.002415665	0.019633747
LOC100691753	20.08990232	1.547508354	0.001953308	0.0168093
mfsd12	52.94683751	1.546769247	0.000174357	0.002698548
apaf1	50.52894258	1.542412776	0.000653795	0.007451103
LOC100698771	12905.92797	1.540111343	0.000973752	0.010005331
psmb1	2218.933807	1.539762081	8.91E-05	0.001601796

LOC100693400	31.76281234	1.537928153	0.001501619	0.013817865
LOC100709772	30.19468777	1.537513844	0.008205685	0.048924222
rab18	722.1211384	1.536672675	2.24E-08	2.16E-06
meis1	465.750121	1.532747228	7.33E-06	0.000237517
pdia6	1010.990973	1.531419477	5.90E-05	0.001152181
fkbp2	128.5450298	1.530193518	0.000253221	0.003598527
surf4	1134.682271	1.524641892	1.76E-06	7.64E-05
ube3d	75.05482741	1.523992822	0.000242753	0.003495196
LOC100708589	251.8370053	1.523719004	0.004176474	0.029664614
LOC100711851	85.42397291	1.521497704	0.003811013	0.027783079
psmc4	2972.316853	1.521140813	0.000290488	0.003981999
psmc1	2923.6039	1.520752687	1.35E-05	0.000375675
LOC100708080	114.5232465	1.520312019	0.004695133	0.032109322
LOC109199824	28.60313229	1.518805211	0.00736037	0.045272851
LOC100707236	195.6744997	1.517175542	8.93E-06	0.000278565
coq10b	4084.951505	1.51555446	0.000543264	0.00644428
bcl6	422.3322017	1.515217853	3.99E-10	7.72E-08
psmc5	2732.275792	1.512106583	0.000517212	0.006223082
ddi2	679.0124667	1.511852096	2.36E-06	9.68E-05
zcchc17	1401.62539	1.509715023	0.000227777	0.003325936
slc35b4	87.67559193	1.509291386	2.63E-06	0.000106699
taf10	572.0185002	1.505237127	3.46E-05	0.000745131
haus7	25.98414135	1.503979585	0.005561967	0.036739815
lg15h2orf72	135.1981016	1.499896549	3.16E-05	0.000697866
smim12	199.7183135	1.499262627	0.000176092	0.002714098
sgpl1	200.0078087	1.498985573	6.10E-07	3.22E-05
LOC100699279	19.56049817	1.498441587	0.004037584	0.028954708
LOC100690252	142.4336025	1.497644095	0.002442662	0.019796245
LOC100704695	1384.001545	1.494806852	1.06E-06	5.06E-05
iqcc	18.37430968	1.493537221	0.002894701	0.022551005
LOC100694567	1475.278859	1.493483048	5.16E-07	2.81E-05
timm17a	2851.903922	1.492781165	9.25E-12	2.73E-09
ankib1	1522.265785	1.492387152	0.002415128	0.019633747
nfatc2ip	147.4988975	1.492371225	0.00063309	0.007264119
spty2d1	207.8863606	1.492236909	2.31E-05	0.000560133
LOC100708067	926.1403534	1.490431919	0.000276605	0.003842703
LOC100707064	651.4719943	1.488548409	0.005464585	0.036212374
ptdss2	324.8539342	1.486453384	0.000150671	0.002416463
LOC100707901	138.3184112	1.484444613	2.93E-07	1.77E-05
mars2	32.31929557	1.48391199	0.001316805	0.012552187
cmpk1	99.22084736	1.483367824	0.00114093	0.011232842
LOC109205072	28.9198718	1.482382046	0.000333801	0.004441446
LOC100712580	15.48108146	1.481298083	0.006588215	0.041712386
LOC112846788	101.1156093	1.47696825	0.00174861	0.015505723
rfk	209.8565529	1.476576084	5.73E-06	0.000195583
LOC100702229	15.97812288	1.474900083	0.000852808	0.009099888
c2cd2l	108.8428319	1.472526348	0.004189381	0.029726396
adck1	44.89535095	1.469186653	3.38E-05	0.000730377

LOC100705818	149.5749961	1.466862154	9.72E-07	4.72E-05
npc2	321.5133927	1.466255473	1.16E-05	0.00033203
ppp1r3g	623.3554269	1.46292798	0.004168493	0.029641654
pigh	110.4142823	1.461796285	0.000165948	0.002594336
uba6	46.26994634	1.461749454	7.76E-06	0.000249239
chek1	36.41238856	1.460336696	0.006224497	0.040034928
spg21	1310.246843	1.460123814	0.000391113	0.005006896
atg2b	1301.790926	1.458611317	4.49E-06	0.00016297
LOC100700062	76.90645934	1.455565754	0.000185477	0.002821322
mtg2	116.7042876	1.453741559	2.78E-05	0.000641819
LOC112842559	50.88941508	1.453524556	0.002134123	0.017925478
abhd6	28.20167995	1.452873385	0.006459665	0.041078432
LOC112842179	47.72529986	1.452241671	0.000888622	0.009374412
sdf2l1	120.0331886	1.446122654	0.000171254	0.002661601
rapgef2	469.9060675	1.444038207	0.001181912	0.011520386
psmc3	2825.794425	1.44135475	0.000203309	0.00303543
clcn5	488.019256	1.439990512	1.26E-05	0.000355189
LOC109203480	43.2866258	1.438912762	0.003902272	0.028201956
fam13b	8898.187701	1.437617472	4.31E-06	0.000157564
LOC102080428	127.1008557	1.436286927	8.16E-05	0.00150274
rnf4	921.3431337	1.43442303	1.86E-06	8.01E-05
smarcad1	31.6591315	1.433836272	0.003876946	0.028084439
LOC100698645	39.8888447	1.431647147	0.005224657	0.034883591
idh1	97.97931081	1.431137204	1.12E-06	5.27E-05
psma4	2411.65141	1.428664158	0.001668893	0.01494286
nup210	98.53295325	1.428138542	7.84E-06	0.000251265
dnase2	64.89066964	1.42753211	0.000654863	0.007454125
pdia4	538.8999431	1.427164939	0.00360049	0.026610699
f5	46.51394059	1.426855306	0.007065318	0.043914855
LOC100703250	17.20507271	1.42647076	0.007466033	0.045837342
slc3a2	16177.33432	1.425550833	0.001754922	0.015526178
LOC100700216	150.220418	1.423769161	0.001944039	0.016768348
abcc10	63.09668838	1.420874789	0.00023711	0.003424551
pof1b	158.2468846	1.42085006	0.004024258	0.028914927
bbs10	19.65327688	1.420094639	0.007482756	0.045888973
LOC100704477	352.266785	1.419357551	3.12E-05	0.000692596
foxn2	665.3714597	1.418899027	0.000128464	0.002130138
LOC100700431	1962.961611	1.416281172	1.26E-10	2.90E-08
ccdc97	194.1212942	1.415971044	0.001696887	0.015127932
yipf6	456.6872337	1.414553951	0.000498688	0.006042778
dhodh	202.8962922	1.414201693	7.38E-09	8.36E-07
psmd6	2776.359221	1.41417312	0.000448931	0.005559448
mtfr1	129.8548513	1.411909828	4.28E-07	2.37E-05
tarbp1	51.3225982	1.408654973	0.001315066	0.012542046
nufip2	60.20717355	1.406661063	0.003865145	0.028020794
LOC102076226	279.6071341	1.398515375	0.004660249	0.031894222
matn2	477.122721	1.39791601	1.45E-05	0.000398453
LOC100697104	246.2478627	1.397750757	0.000395764	0.005035222

LOC109196527	18.60354507	1.397508179	0.004347957	0.030453657
larp4	854.3239852	1.396864625	1.42E-10	3.19E-08
zfand2a	496.3378909	1.395767339	8.95E-06	0.000278608
psma5	1750.378902	1.395628025	0.001023135	0.010358401
LOC100711005	204.803808	1.3942286	1.59E-05	0.000425526
psmd12	2515.223242	1.394202781	0.000191619	0.002905227
haus8	23.73618161	1.393543008	0.003631865	0.0267787
LOC100700222	607.0157094	1.393085734	7.13E-05	0.001344749
LOC100701982	398.2677344	1.392531147	0.004619841	0.031701714
psmb2	1179.389555	1.3925284	2.27E-05	0.000554743
coil	73.9006738	1.391501762	0.00060179	0.006990804
klhdc4	114.9479132	1.390907303	0.000168543	0.002626052
LOC100706111	7639.885016	1.390840823	0.002858845	0.022337205
pgm3	24.20881162	1.389469732	0.00272377	0.021526908
lmln	45.4764312	1.388863088	0.001304134	0.012476197
LOC109201566	82.530623	1.386452021	0.000604633	0.007011175
brf2	84.20158045	1.385760882	0.000293309	0.004011792
armc8	6346.118245	1.385407287	3.21E-05	0.000702915
LOC100699257	80.41578396	1.385205794	8.45E-05	0.001539904
idnk	74.41215185	1.384404169	0.000107219	0.001852217
LOC100709896	528.5106711	1.382025129	0.000218634	0.003225711
dnajc21	453.7773502	1.381909713	3.32E-05	0.000720069
syne2	862.0918506	1.379047314	0.007334193	0.045141718
pi4ka	118.0088775	1.377694988	2.42E-08	2.29E-06
pitrm1	817.0555065	1.376562603	0.000128676	0.002131763
ttl	160.9806727	1.374723457	5.54E-06	0.000190441
LOC100709275	45.38961761	1.374539609	0.001745786	0.015489584
LOC100695037	53.25124568	1.373729619	3.04E-05	0.000682645
matn4	213.4372359	1.372827441	0.001851137	0.016162016
LOC106098040	75.99491076	1.370292375	1.07E-05	0.000314023
mtrex	316.3634785	1.367186318	2.18E-06	9.14E-05
txnl4a	352.4392022	1.367182672	0.001016019	0.010308824
LOC100699792	315.3036371	1.366027118	0.000650183	0.007423742
LOC100693082	95.72447747	1.365227215	0.000233644	0.003386944
aldh1a2	122.7232608	1.365152484	0.000133652	0.002200566
LOC100693833	56.81722805	1.364197063	0.004137687	0.029478348
LOC100710224	14.26831455	1.360252019	0.007003979	0.043761334
zgpat	90.81820265	1.360205007	9.59E-05	0.001696515
scamp1	877.3291696	1.359376581	6.94E-05	0.001322232
nfil3	301.097334	1.357016024	0.000861226	0.009168656
LOC100700167	726.0448896	1.356783024	6.61E-07	3.45E-05
stoml2	482.1822674	1.35640229	0.000476101	0.005818391
rabggtb	1327.152507	1.356134059	0.0005178	0.006223082
tmem64	85.55005866	1.355898756	0.007533554	0.046063715
gclc	233.3075782	1.354508304	5.84E-06	0.000198821
LOC100708185	2599.800425	1.353632743	0.000425017	0.005334376
sbno1	344.3216075	1.353527504	4.56E-05	0.000925409
sel1l	776.3430214	1.351003432	9.12E-08	6.95E-06

ypel5	942.5738238	1.350052436	0.000392327	0.005012074
LOC100691065	57.75322686	1.349735454	0.001364427	0.012880635
rtca	597.4490543	1.348075582	1.37E-05	0.000377526
LOC100701033	2999.588875	1.346857713	2.62E-05	0.00061603
LOC100701225	221.9928922	1.346239342	9.39E-05	0.001666957
LOC100692767	864.2135652	1.346017439	0.000752534	0.008296622
lrwd1	51.88808328	1.343640956	0.00097021	0.009980222
npc1	460.914934	1.342719383	8.46E-05	0.001539904
upf3b	1126.222634	1.342274746	1.19E-07	8.35E-06
prpf3	884.2070238	1.341891492	0.006940301	0.043436467
LOC100705239	988.7828768	1.340806741	5.62E-05	0.001104399
nup93	104.7292883	1.340793212	0.000196329	0.002959742
LOC100710017	111.9903909	1.340669604	0.002743733	0.021665465
bend7	165.4081188	1.339792667	0.001438768	0.013412145
LOC100696072	698.5383895	1.338671946	1.46E-07	9.71E-06
LOC100704850	8678.045387	1.338303665	0.003196365	0.024369988
LOC102080582	2118.727168	1.337580921	0.001347927	0.012776784
LOC100709882	51.30555513	1.336573893	0.001633891	0.014689657
LOC100704070	597.1743889	1.336410177	0.000143971	0.00233585
LOC100698375	714.8444748	1.336223116	4.42E-08	3.86E-06
LOC100706085	354.9270871	1.334193286	0.001938607	0.016729257
LOC100695556	29.25348103	1.334158351	0.001494446	0.013779607
dhrs1	1949.309852	1.333561541	0.004320464	0.030373975
tars	307.4490637	1.333177544	5.13E-06	0.00017922
LOC106098454	47.93607074	1.33103178	3.47E-05	0.000745635
yif1b	199.5922444	1.328761354	8.29E-05	0.001521984
lg10hxorf57	169.7167643	1.328216763	0.002672802	0.021204455
LOC100706651	116.2838744	1.32392129	4.17E-05	0.000857551
ppm1d	245.37386	1.323786486	8.73E-07	4.31E-05
LOC100693277	1182.659504	1.323499686	0.00234369	0.019225919
LOC100706302	467.8695369	1.323417534	1.32E-08	1.37E-06
ppox	338.9770539	1.323386181	0.00142633	0.013309546
mrpl20	608.7124472	1.322663153	0.000108781	0.001868821
LOC112842376	435.626944	1.321564454	0.00018039	0.002764296
glut1	4498.234337	1.320435586	0.000890592	0.009389862
dis3l2	1054.303898	1.315878046	1.71E-05	0.00044883
alg5	201.3944612	1.314690603	9.38E-06	0.000287266
map1lc3b	2377.627055	1.313339955	0.000246428	0.003542614
LOC100690674	173.5568883	1.310952282	5.64E-06	0.000193035
LOC100695066	163.5547344	1.310612038	0.000275532	0.00383923
ect2l	28.05414439	1.309828316	0.001012539	0.010284739
leng9	331.4847161	1.309737723	0.000127991	0.002124196
psmd2	4760.643809	1.309590341	0.000114157	0.001943183
tnip1	1315.819231	1.308771846	1.30E-08	1.36E-06
dhx40	103.9368892	1.307643181	0.002821191	0.022098738
LOC100705047	35.12599321	1.307563276	0.000401412	0.00508966
dnajc11	456.9970612	1.307411797	0.000688018	0.007746138
LOC102079739	3084.80231	1.304466929	0.000922819	0.009663864

pigu	95.95166326	1.303424207	8.38E-05	0.001532802
LOC102077187	269.1300528	1.302202866	5.53E-05	0.001089481
atp6v1e1	204.4667532	1.301517284	4.63E-08	4.02E-06
LOC100697336	241.329129	1.30078956	1.33E-05	0.000371609
LOC100534438	694.1125157	1.299909474	7.98E-07	4.02E-05
srd5a1	88.56597355	1.298135294	0.00256103	0.02054886
ppp1r37	2190.579324	1.296602351	3.79E-05	0.000795857
anapc4	179.1717476	1.295000234	0.000515791	0.006216987
etfdh	5047.519478	1.294630686	0.000114469	0.00194671
ppp5c	293.1707188	1.294205587	0.001125613	0.01115293
snrnp200	1216.232355	1.293193316	0.002448131	0.019828261
LOC100693985	534.7379239	1.292021623	0.000165949	0.002594336
LOC100709921	601.8609129	1.291772799	0.000149274	0.002408603
chchd4	142.693941	1.290729582	0.002066783	0.017491095
LOC100698868	33.24363923	1.289967124	0.004005556	0.02881396
maf1	7859.260288	1.289365474	7.18E-06	0.000233231
bola3	118.5236261	1.287241046	0.004387615	0.030602995
sdha	9865.657559	1.286488427	3.40E-07	2.00E-05
npepl1	1825.406712	1.28616707	7.86E-06	0.00025149
LOC102081602	47.79929186	1.286046336	0.000471393	0.005779854
abcb10	239.6457575	1.285695724	0.001414291	0.013243746
dock5	254.2644134	1.285120427	0.000247287	0.003547952
klhl24	1765.119849	1.284799678	0.004419743	0.030746323
calcoco2	10306.95357	1.283921495	0.000226654	0.003317362
bckdk	10202.43186	1.283529584	5.00E-06	0.000175716
LOC100701093	2130.458732	1.28314385	0.004955797	0.033534163
psmg3	211.874671	1.28282738	0.002391993	0.019526175
yme1l1	538.267779	1.282265284	1.66E-07	1.08E-05
LOC100697981	627.5289437	1.281228328	4.12E-07	2.32E-05
LOC100690629	148.1884127	1.281001158	1.06E-07	7.66E-06
LOC100709409	215.8760344	1.279714105	1.07E-05	0.000314023
LOC100693443	1176.62397	1.277538265	3.63E-05	0.000772117
slc35c1	128.0456501	1.277518509	0.000559706	0.006576373
LOC100707699	263.9243652	1.276683382	0.007866809	0.047538444
optn	4284.850319	1.274143125	0.000650196	0.007423742
LOC112843372	57.82964338	1.272983371	0.001117641	0.011103535
foxo3	7530.770942	1.272485998	0.002523376	0.020313777
LOC100698325	4712.194572	1.271030048	7.00E-09	8.08E-07
dirc2	139.2467881	1.269522985	2.69E-06	0.000108295
nup62	426.6540715	1.26945602	3.06E-05	0.000684347
rgmb	32.07106525	1.26840452	0.005079124	0.034194407
acbd5	1289.889982	1.268317682	4.82E-11	1.21E-08
usp38	660.5295359	1.267911965	0.000154297	0.002455543
neu4	64.32327057	1.265726011	0.001639329	0.014720698
hif1a	2087.961273	1.265603645	4.61E-05	0.0009327
bnip3	2428.01527	1.265282432	0.001177611	0.011502593
glod4	571.2411457	1.264994181	0.000388895	0.004994507
arrdc3	5782.897033	1.263810775	2.27E-05	0.000554743

hook3	222.5071999	1.263752931	0.001163729	0.011408965
wbp1l	1441.053519	1.26322329	0.006660167	0.042094248
prpf31	503.3258822	1.26008439	0.003885265	0.028100119
LOC109194649	25.94005148	1.258229856	0.001020485	0.010337202
LOC100703082	55.99326148	1.257711716	9.62E-05	0.00169742
LOC100711018	3296.715381	1.257020903	4.03E-07	2.28E-05
psma3	2947.740615	1.256667284	0.001462519	0.013576954
acsf3	309.8619978	1.255801649	0.005752007	0.037793675
atg2a	1623.185441	1.255565652	0.000243663	0.003505573
ctsd	5777.211482	1.252432235	0.000173244	0.00268578
LOC100702809	21.03719907	1.251874968	0.002314513	0.019070106
LOC100711430	138.1744993	1.251388695	0.007286784	0.044939194
LOC100705739	525.7610847	1.250954468	8.02E-07	4.03E-05
pnpt1	146.3349801	1.249832268	0.001426008	0.013309546
slco5a1	64.15869311	1.248994416	0.000796538	0.008628236
arl14	3063.207271	1.248679317	0.0004725	0.005785795
LOC100694172	207.1518669	1.248131817	0.000197201	0.002968074
galnt15	146.6605718	1.24507361	0.006588528	0.041712386
LOC100711855	165.9064659	1.244435509	0.000623283	0.007180872
pdk3	7042.541009	1.244176048	1.78E-06	7.70E-05
cdc37	1192.993832	1.240810747	0.000259536	0.003671434
LOC100709870	432.7213164	1.240695365	7.34E-09	8.36E-07
sema7a	761.6727168	1.238383847	0.005951131	0.038786686
gpr157	981.0878711	1.23799644	0.001073821	0.010771824
prim2	41.94337352	1.236768109	2.69E-05	0.000626335
LOC100710102	199.390842	1.235758925	1.16E-07	8.22E-06
LOC109195496	320.5444459	1.234563283	0.000100967	0.001760579
LOC102082093	50247.42565	1.233576621	0.005901803	0.038532738
spata5	83.66062579	1.233249734	0.000365705	0.004773677
mfsd10	32.33487971	1.233113301	0.004993833	0.033742411
LOC100697374	100.5334793	1.233096212	0.000231623	0.003366228
LOC100692472	5416.739484	1.229902649	0.001737954	0.015442207
usp5	1270.357186	1.229711152	0.000535312	0.006370282
tbck	47.58357198	1.229703015	0.003291579	0.024922144
grina	723.4521436	1.229328548	0.001498914	0.013806646
LOC100694482	6210.536179	1.228504304	0.000124719	0.002079165
strap	480.5891608	1.227555636	1.49E-05	0.000407152
fastkd2	90.50031707	1.226593294	0.001201488	0.011650113
LOC100697449	358.2130781	1.22646104	8.62E-06	0.000271248
pithd1	403.9028312	1.225579628	0.002432394	0.019749378
prpf19	199.260417	1.225344612	0.001179367	0.011513699
LOC100690071	115.4230863	1.225135931	0.00035138	0.004615872
mfsd11	152.6128944	1.224378384	0.000257277	0.003647794
LOC100708833	193.1456925	1.224184526	0.000203202	0.00303543
ppig	202.0637534	1.22365427	0.000463065	0.005696529
LOC112842127	23.39555736	1.22251063	0.003439266	0.025788251
LOC100694921	460.4145562	1.219167556	0.001292684	0.012392166
LOC100699396	144.8040443	1.218445418	2.15E-05	0.000527007

srrt	561.1182753	1.217870218	2.42E-05	0.000581898
LOC112847895	92.53608396	1.217086968	0.001908709	0.016555798
hps3	584.8871646	1.21525274	0.006215075	0.039994046
mthfs	148.0839105	1.214810499	3.03E-05	0.000682645
vezt	1712.02463	1.213925088	0.003333186	0.025165421
arrdc1	219.2417406	1.213861752	0.007850185	0.047494543
nsmce1	23.71139178	1.207978076	0.001059976	0.010667478
tyw5	52.47232761	1.205588934	0.008267698	0.049160999
fkbp4	2595.718651	1.205539952	0.000115513	0.00195908
psmd13	2950.552853	1.205522109	0.002058955	0.017451823
LOC100706108	70.15056886	1.2038823	0.004875531	0.033087392
afg1l	68.21895675	1.202827543	0.003656422	0.02691706
LOC100707627	3839.285017	1.202477474	6.37E-06	0.000212629
LOC100692578	81.3138826	1.201921017	0.006932175	0.043400227
tbpl1	354.7042197	1.200588339	0.000567615	0.006652476
LOC109197106	124.7317635	1.200269984	0.000339424	0.004496946
LOC100693335	426.5196919	1.199724887	0.000527602	0.00630679
pcyt1b	4298.540722	1.196559031	1.73E-06	7.56E-05
LOC100696961	1204.327288	1.195850009	0.001140753	0.011232842
znrd1	99.1815622	1.194127093	0.001074992	0.010777755
LOC100693037	99.60074028	1.193888749	0.002497336	0.020139039
ccdc126	130.1736851	1.193170579	2.70E-05	0.000627047
mmadhc	1161.9409	1.193137839	2.60E-05	0.000612731
tmem165	364.2945913	1.193093328	1.60E-09	2.28E-07
slbp	118.662632	1.192486775	0.000532841	0.006353078
mtmr14	537.853264	1.190600701	2.80E-06	0.000111667
lmnb2	345.1398143	1.189423109	1.31E-05	0.000367124
LOC100703776	198.5655587	1.188557679	0.005321331	0.035389232
ruvbl1	139.077465	1.188285906	0.000118643	0.002006668
LOC102075613	90.56603537	1.187550133	0.004785823	0.032657443
LOC100694605	490.8466292	1.186908052	9.38E-06	0.000287266
med16	134.5733202	1.186156889	1.68E-05	0.000442456
trmt1	156.6768166	1.186083169	0.001029662	0.010413139
tmem33	317.7892056	1.185602232	6.99E-06	0.000228911
mrpl22	310.9496939	1.185574488	0.002599859	0.02077652
LOC100698501	2502.582399	1.184060825	0.001913766	0.016565503
LOC100704949	30.22857847	1.184025111	0.00213616	0.017934478
alkbh4	143.0996316	1.183402463	0.000784391	0.008561551
stard3	207.8630713	1.182023078	3.13E-05	0.000692596
arntl	554.603352	1.181481311	0.000266482	0.003744036
pign	30.62368479	1.180159273	0.005400819	0.035864119
atp13a2	39.45766856	1.17862053	0.007385151	0.045395236
lcmt2	127.5834952	1.177749437	9.23E-05	0.001646987
gramd4	505.8451141	1.17621469	0.00096136	0.009922135
psmd10	344.9094789	1.176088854	0.007231642	0.044753839
nudt16	868.2105946	1.175558531	0.001042423	0.010530736
sec24d	292.8587767	1.175534447	0.001879155	0.016368194
gemin5	249.0377955	1.174106187	0.008004665	0.048074546

LOC100693286	58.78781923	1.169720696	0.003795194	0.027751797
LOC100700175	1159.189374	1.169713702	0.001564109	0.014237838
evi5l	66.7214861	1.168351481	0.00407665	0.029178582
samd4a	1368.447359	1.166290086	0.000793033	0.008611197
arl5b	2387.324986	1.166174276	1.55E-06	6.89E-05
stam2	454.7042247	1.163735604	2.42E-07	1.49E-05
dnajc15	317.526098	1.1632917	0.004424527	0.03076809
prkab1	107.8628349	1.162596689	0.003706455	0.027220695
LOC100711139	484.3749948	1.162200624	0.003841485	0.027881894
selenoo	581.9566106	1.161909054	0.006562454	0.041616231
	27898.43944	1.161625028	0.000136994	0.00223962
dennd4b	1371.794446	1.161132862	4.38E-05	0.000896198
psmc2	3347.403767	1.161068651	0.001656057	0.014842233
LOC100691011	200.9125906	1.156579624	0.002893187	0.02254866
LOC102083256	47.85511416	1.156232062	0.000179556	0.00275606
dohh	726.7681959	1.155684209	6.86E-05	0.001309266
cdkn3	218.7684947	1.154197878	0.000253748	0.00360051
cpd	944.4751496	1.153999284	2.16E-07	1.34E-05
onecut2	40.59786692	1.153737165	0.000986492	0.010082653
LOC100700343	16.70029694	1.152256229	0.005687479	0.037422604
LOC100711553	286.6163941	1.151469385	0.001352672	0.012802175
timm23	700.7593936	1.150668843	2.91E-05	0.000657354
clns1a	574.3257756	1.150519317	0.002925878	0.022736711
LOC100693638	955.764276	1.150359944	9.76E-09	1.06E-06
mcm7	190.8009256	1.149633841	4.06E-05	0.000840969
nol6	100.4935577	1.149492055	0.00109524	0.010939449
tbc1d17	1422.799133	1.148794921	0.000264032	0.003723688
LOC102079177	46.43935904	1.147523762	0.003022816	0.023372758
LOC100706012	87.53477987	1.14674415	0.000593329	0.006916174
retreg2	1162.975641	1.144720481	0.000477686	0.005833925
prpf18	1007.531477	1.144306101	0.000625118	0.007186449
bbs2	127.9879766	1.143740873	7.47E-06	0.000241614
LOC100702624	48.60794932	1.14299819	0.0003662	0.004776785
LOC100702830	452.9556041	1.142675626	0.000282063	0.003886573
lman1	268.45672	1.142550111	0.000149124	0.002408271
tacr2	87.83694353	1.142543197	9.74E-06	0.000295528
LOC100691978	1426.530826	1.14127944	0.001364411	0.012880635
LOC100696442	245.7553764	1.140179059	0.008092872	0.048482948
atf6	212.0009941	1.139929601	0.001266997	0.012177324
LOC100709252	319.2637629	1.139885671	3.64E-06	0.000137395
guf1	129.0901591	1.139347798	0.001394899	0.013107942
cdipt	91.35715347	1.138539211	9.88E-05	0.001729576
canx	719.0699129	1.136643823	0.000373885	0.004853191
LOC100698618	5396.875614	1.135025448	0.003962081	0.028567557
LOC112847064	29.84030291	1.134967864	0.005541815	0.036645769
LOC100690853	307.3932465	1.133960393	0.002787399	0.021907898
esyt2	903.2983275	1.132786303	1.77E-08	1.76E-06
hapln3	98.64617443	1.131630138	0.001261379	0.012135874

tsta3	81.16314051	1.130300358	0.003839172	0.027881894
slc30a5	193.658064	1.129610647	2.02E-05	0.000501411
erp44	289.019152	1.129518701	0.00028891	0.003966223
LOC100706228	32.00112459	1.12927791	0.006259183	0.040174776
uso1	424.5395787	1.12752979	0.000250773	0.00357467
lrp12	773.8093044	1.127118279	1.89E-10	4.04E-08
paqr3	92.75863183	1.126658201	0.000151248	0.002423616
impad1	87.13678684	1.126031212	5.31E-05	0.001051416
narf	444.2945393	1.124842985	2.63E-05	0.000617839
kctd9	317.236706	1.123885743	9.17E-05	0.001641295
LOC112847121	171.3607448	1.123037948	1.54E-05	0.000417403
LOC112846785	289.1706672	1.120935486	0.004409054	0.030694943
cby1	78.73442528	1.12066446	0.005531725	0.036594783
slc25a40	360.8877985	1.120179518	0.000331613	0.004421828
slc25a34	462.7114868	1.119404702	3.10E-05	0.000689013
LOC100691964	69.60528528	1.118562916	0.001687102	0.015062365
sf3a3	535.2084935	1.118452873	0.008229698	0.049015346
dnajc3	593.3127497	1.118288262	1.94E-05	0.00048869
LOC100697817	682.8723035	1.117582656	3.61E-07	2.07E-05
pigv	33.78057953	1.115883216	0.002225089	0.018505576
LOC102077528	211.3050329	1.115564287	0.00152836	0.013987952
LOC100707250	822.1514439	1.115267961	0.000525571	0.006288381
LOC100701101	213.8420959	1.115192247	0.006318607	0.040490572
herpud1	379.6276957	1.115166039	1.34E-07	9.15E-06
LOC100693874	358.1063378	1.1147967	0.00413065	0.029462976
mrps23	435.3354364	1.113834611	0.007198265	0.044615321
LOC102077663	195.5684974	1.113459522	0.004942219	0.033491056
LOC100696261	2939.519404	1.111868511	0.002373265	0.019399402
tmc6	527.7763633	1.111393494	3.03E-05	0.000682645
pop1	90.4253634	1.110929007	0.001544123	0.014097329
LOC100706152	6168.303121	1.108833464	0.000153883	0.002453161
cc2d1b	296.8138805	1.106619205	0.002837566	0.022208287
LOC100691517	1018.4564	1.106376493	0.002846758	0.022261482
LOC100708200	689.1453172	1.10440896	5.89E-06	0.000199829
dnaja2	2603.571034	1.103266738	1.08E-05	0.000315568
LOC100690111	56.77616555	1.102776515	0.001006857	0.010251932
kpna6	2857.406231	1.102442501	6.29E-07	3.30E-05
babam1	117.0390547	1.102033629	0.003156015	0.02412171
bet1	118.571085	1.100491582	1.45E-06	6.55E-05
nfe2l1	3727.974239	1.099550096	0.000976375	0.010021456
LOC112843316	90.63224646	1.099524899	0.000140004	0.002284804
tmem255a	1786.371927	1.099339562	0.002598883	0.02077652
LOC112848170	231.4400861	1.098350124	0.001131633	0.011196217
LOC100706058	432.9601907	1.0981892	0.001064973	0.010706178
LOC100696541	470.6307964	1.097906537	0.000908306	0.009538748
npl	62.91605188	1.097485524	0.00038587	0.004974025
LOC100697207	2554.937946	1.096603428	0.000183321	0.002797689
rab24	733.8298869	1.096027918	1.09E-05	0.000316626

LOC102078181	277.8201683	1.095943332	7.28E-10	1.24E-07
LOC100697085	191.1095245	1.095334857	0.000370375	0.004821095
heatr5b	281.8760296	1.094382857	1.01E-05	0.000303224
rragc	464.3413472	1.091468008	0.000207004	0.003083171
eif4ebp2	1040.122827	1.091430433	2.65E-05	0.000618592
plekhm2	862.0695732	1.091047466	0.002334172	0.01918956
cacybp	283.6073024	1.090735935	0.000560621	0.006582953
oser1	1837.674087	1.089940522	0.000135343	0.002223004
LOC109202475	83.29730654	1.089113019	0.004645426	0.031827931
hspa9	6240.648421	1.088998656	1.97E-07	1.23E-05
LOC100709579	245.312512	1.088584974	0.006984964	0.043686576
lig1	186.9075069	1.087259309	0.00681193	0.042951205
LOC100690829	31.19986426	1.086826985	0.004341892	0.030440381
lrpprc	1322.735383	1.085238023	0.003342466	0.025225239
LOC100696864	119.3011992	1.08504112	0.001470048	0.013622851
slc37a3	352.8601238	1.08407951	1.07E-07	7.69E-06
wdr92	37.92128463	1.083724933	0.002465837	0.019936999
LOC100694047	49.79716972	1.083084837	0.00831537	0.049350604
rpn1	904.0381451	1.081618585	0.000974266	0.010005331
LOC102080651	63.67395624	1.080792565	0.008414854	0.049805389
ppp4r2	375.7384812	1.080128808	2.95E-08	2.69E-06
emc9	171.3882388	1.078839164	0.007190746	0.044583586
eif2ak1	322.5769642	1.078161851	3.41E-07	2.00E-05
znf821	83.96331782	1.078084397	0.001895488	0.016464177
LOC109194335	334.4820124	1.077580188	4.64E-08	4.02E-06
keap1	3780.796741	1.076939334	0.000484992	0.00590375
snrpd1	279.3957837	1.076731582	0.006463341	0.041087753
LOC100708797	562.8348428	1.076359736	0.003151911	0.024103496
nmt1	3336.162223	1.076351674	0.006930857	0.043400227
rab6a	2085.211234	1.075588663	3.29E-05	0.000714572
rnf157	408.7852114	1.075570604	0.001494496	0.013779607
LOC100689881	628.5866688	1.074329157	0.003465094	0.025929615
LOC100709095	1007.039329	1.07430474	0.000310462	0.004203106
LOC102081620	92.57082192	1.072520592	0.001112547	0.011082546
map1lc3a	7898.991484	1.072299103	8.39E-05	0.001532802
katna1	89.64550529	1.071805738	0.001976623	0.016947168
LOC100534467	52.63985122	1.071311497	0.0082815	0.049212444
LOC100705570	284.9309018	1.071295169	0.000218657	0.003225711
ache	898.0756291	1.070926337	3.25E-05	0.000706853
psmd3	4894.586369	1.069481877	0.004450076	0.030887978
LOC100700294	69.89465672	1.069391386	0.005178233	0.034673269
LOC100692481	298.7089547	1.067644334	0.006450956	0.041037086
rap1gds1	122.6048491	1.067599782	0.003285965	0.024910079
LOC100705613	772.4241295	1.066769043	1.06E-05	0.000313544
LOC100709156	261.814534	1.066069212	7.09E-06	0.000230655
tpbg	240.2865059	1.065570254	0.001790028	0.015755138
ecd	181.2389006	1.064800508	0.001681525	0.015034245
eif2ak3	205.866799	1.064745115	0.004972683	0.033623947

LOC100703352	170.4046073	1.064114347	6.05E-05	0.001178135
nt5m	259.6915097	1.063846777	0.006651224	0.042052027
LOC100711371	171.6210875	1.063791019	3.24E-05	0.00070576
LOC100704999	56.16196003	1.063684362	0.004227413	0.029897577
ttll11	137.563071	1.063144953	0.001001999	0.010211156
LOC100697124	20.54911038	1.062149098	0.007490762	0.045922916
LOC102083250	193.5723551	1.061756353	0.007564339	0.046221545
exosc6	200.354927	1.061435439	0.00409244	0.029235307
lonp1	843.3758655	1.061316697	1.59E-06	7.03E-05
prpf6	1139.727121	1.060559967	0.007466409	0.045837342
LOC102080688	69.44133288	1.060441847	0.000393545	0.005022467
pikfyve	190.6573776	1.060439921	0.001108235	0.011051432
galnt5	83.53384137	1.058325544	0.000112147	0.001915978
oxnad1	425.6981183	1.05754378	0.000459012	0.00565067
LOC100691916	684.3337781	1.057303987	0.000479501	0.005852238
trmt61b	292.0190976	1.056594387	0.002264255	0.018755783
tmem246	186.8684929	1.055248861	0.006710759	0.04238518
LOC100700820	487.9773622	1.054574345	1.28E-06	5.85E-05
spryd4	4562.423081	1.053241917	0.007063743	0.043914855
LOC100690823	695.0063195	1.052165303	0.000128846	0.002132677
rp2	100.2456527	1.05110022	0.000742488	0.008215102
LOC100706026	307.8885473	1.050233817	8.72E-06	0.00027341
LOC100706200	1429.236414	1.050038764	0.005526347	0.036582529
LOC100710852	938.2839304	1.049304154	0.000606629	0.007029933
LOC100694127	301.0453493	1.049283118	4.33E-06	0.000157735
LOC100694211	93.80615756	1.048771139	1.99E-05	0.000498419
LOC100703199	428.063459	1.048181876	0.005085308	0.034223642
slc25a32	267.4288698	1.047062848	0.000884492	0.009346755
als2	452.5954483	1.046478076	0.004376923	0.030574312
LOC100707757	102.4951456	1.044838435	0.001185199	0.011529881
znf704	237.1181653	1.043774136	0.001154697	0.011338352
LOC100702973	239.2920984	1.043522675	0.003741601	0.027403023
trip6	999.1416093	1.043271794	0.001216	0.011760151
LOC102076856	31.87636873	1.042192818	0.003164929	0.024179903
ube4b	1460.656342	1.041783817	0.000420228	0.0052814
atp13a1	179.5819179	1.041773971	0.000520247	0.00624296
mrm3	52.47526439	1.041362974	0.008157846	0.04874375
LOC100712210	350.5909072	1.0409918	0.001286019	0.012341002
LOC100696920	665.5410522	1.040742438	0.004591261	0.0315732
LOC106098620	34.05179141	1.040639872	0.00773007	0.046941045
catip	33.43532728	1.039316518	0.0068998	0.043335662
rer1	260.3408615	1.037057234	0.000220258	0.003244184
uhrf1bp1l	1037.108363	1.036687891	0.001135825	0.011216155
ccdc167	73.60796752	1.036462514	0.007969644	0.047957183
LOC100700311	682.3099871	1.036014309	0.000841959	0.009020362
slc33a1	92.13458273	1.034722086	0.00066413	0.007541143
LOC100702139	448.9035844	1.033756356	9.60E-05	0.001696515
msl1	612.8112431	1.031114437	5.53E-05	0.001089481

faxdc2	116.0199272	1.030791004	0.007476897	0.045868174
chmp2b	2784.013474	1.030596687	0.000510621	0.006171276
LOC100692842	1493.41134	1.030336613	0.002786071	0.021906724
pgam5	235.064095	1.029677614	0.001284131	0.01232925
LOC100702835	1521.258382	1.028636007	1.87E-06	8.04E-05
dcun1d2	208.3046298	1.028165577	0.002572075	0.020616531
derl1	726.7584971	1.028139805	0.001252711	0.012064967
ace	136.9949298	1.02796124	0.001910594	0.016556698
LOC100700023	1545.21352	1.027575073	2.69E-06	0.000108295
LOC100707936	1513.192756	1.0269833	0.002392975	0.019526175
rab5a	1157.741987	1.025639193	6.90E-05	0.001315422
LOC100705360	432.5271342	1.025199664	0.005488981	0.036348125
cog7	320.3353087	1.024971837	0.003886669	0.028100119
gpd2	164.9673737	1.024292797	0.000215799	0.00319623
rundc3b	149.7152657	1.024132782	0.005031663	0.033948658
LOC100698909	444.4409851	1.022052943	0.003917356	0.028277987
ssb	721.1873145	1.020516488	0.007776021	0.047158462
psmg4	55.26339203	1.020502921	0.006369035	0.040708078
LOC100710341	800.7644991	1.020404433	2.82E-05	0.000641819
LOC100696835	2260.278618	1.019592727	0.005983428	0.038942563
LOC100698454	27.10916106	1.017444668	0.004456435	0.030897506
abcf2	825.3827693	1.016912275	0.000177246	0.002727364
LOC102083268	174.8138107	1.015837144	0.008327006	0.049403891
usp40	174.1649319	1.015568229	0.000622049	0.007177313
selenbp1	1438.628809	1.015353386	0.001472745	0.01363316
LOC109202005	188.7296791	1.014751377	0.005591116	0.036893033
slc35e1	437.5011633	1.014656333	0.001336191	0.012691426
LOC100699319	187.0866017	1.01416596	0.004051719	0.029033678
pgd	129.7814755	1.013764314	0.000133777	0.002200566
LOC100704614	185.9694927	1.012659469	9.67E-05	0.001704494
snap47	608.635436	1.012395713	0.000525723	0.006288381
LOC100706901	3614.156327	1.011553034	9.23E-05	0.001646987
LOC100692923	1573.625221	1.009718881	0.000209775	0.003111967
LOC102075638	706.1830802	1.009441077	0.002181061	0.018253743
LOC100704967	818.5784651	1.009108863	0.000680385	0.007693333
LOC100702592	64.51102714	1.008061516	0.00582956	0.038195225
LOC100710659	426.9920879	1.007789185	2.01E-05	0.000501402
LOC100703463	775.1876416	1.006096713	0.003099971	0.023810853
LOC109202003	200.6634826	1.006094266	0.007746449	0.047009795
LOC112843442	230.0296223	1.004849228	0.002311303	0.019052376
vldlr	160.0137002	1.004183659	0.000710024	0.007945776
LOC100702443	4516.63475	1.003473487	0.000402811	0.005096977
ephx1	468.9440597	1.001788497	0.003424176	0.025685462
pdcd4	279.4650843	1.000912339	0.001964624	0.016875431
kiaa0513	74.35045642	1.000510737	0.001557201	0.014188845
eef1g	37047.95104	-1.000000744	5.22E-05	0.001040485
dapl1	267.5750028	-1.000482689	4.35E-05	0.000892455
dcaf17	232.1454034	-1.000561355	8.09E-05	0.001493727

LOC100703462	26362.57065	-1.000628575	0.000835095	0.008972683
ogn	492.890552	-1.001587622	0.006121638	0.039551269
LOC100706902	2370.300746	-1.001967364	0.000497715	0.006034918
LOC100692695	282.3894176	-1.002139116	0.007265896	0.044869927
LOC102080542	51.5139574	-1.002947325	0.001946896	0.01677902
LOC100708129	180.7022178	-1.003444252	9.06E-06	0.000281118
mapk6	7088.393823	-1.004178615	0.000433699	0.005410763
LOC100698697	716.6006552	-1.004920101	0.000794925	0.008616113
pm20d2	179.3787151	-1.005238597	0.001126693	0.011157686
LOC102082912	32.97032925	-1.006587863	0.00625583	0.040174776
rpl30	6543.955713	-1.007299807	2.02E-05	0.000501411
LOC106098137	107.2658781	-1.007391162	0.002705743	0.021410966
rpl17	15205.16139	-1.00846443	6.96E-05	0.001323277
ddx21	19272.7195	-1.009182224	9.11E-07	4.45E-05
trim13	290.483587	-1.009791024	0.000727738	0.00810497
LOC100707324	599.3203918	-1.010433818	5.15E-05	0.001029946
phlpp1	424.4646494	-1.011268158	0.000402303	0.005094005
LOC100694619	72.01162764	-1.011442102	0.000981711	0.010059566
LOC100697283	993.4452709	-1.011648483	8.69E-05	0.001570507
phospho1	67.59512742	-1.011718861	0.002366543	0.019370015
LOC100704302	4500.116196	-1.012184041	0.001500159	0.013811272
LOC100703532	316.970398	-1.012254141	1.04E-05	0.000311475
fgf11	22414.80365	-1.012404034	1.66E-09	2.33E-07
LOC100705098	834.3086438	-1.013301067	0.003303605	0.024992842
LOC100691757	110.5366768	-1.017938493	0.00073353	0.008154813
LOC100701567	1983.670501	-1.018869677	0.002977876	0.023092518
smpx	13423.34036	-1.019009602	0.000683042	0.00770412
LOC100708400	1673.358741	-1.019641175	1.10E-05	0.00031781
rps27a	16857.5234	-1.019703783	9.23E-05	0.001646987
fbxo9	1978.702715	-1.01973288	4.96E-06	0.00017535
LOC100693200	379.6009527	-1.020420698	0.00071244	0.007963218
LOC100709204	1472.159027	-1.020531231	1.33E-07	9.14E-06
arhgef16	13988.5252	-1.022609468	1.31E-07	9.05E-06
LOC100698832	335.8770028	-1.02413653	0.000250372	0.003571696
nat1	54.04828089	-1.024999296	0.000391541	0.005008925
pdcl3	5693.136052	-1.02614669	2.45E-07	1.50E-05
LOC102082270	63.67219585	-1.026270259	0.003316293	0.025068424
ncoa6	795.5626198	-1.02628649	3.04E-05	0.000682645
LOC100692997	137.1654942	-1.027204102	0.001088357	0.010888258
rpl7	28598.4893	-1.027653382	1.49E-06	6.64E-05
LOC100702758	16736.00952	-1.029143528	1.78E-05	0.000462568
LOC102077761	119.4572759	-1.029204073	9.84E-05	0.001723434
rps7	15686.62665	-1.029240475	5.57E-05	0.001095354
rps23	10743.99781	-1.029525936	2.64E-05	0.000617839
fzd2	31.09951539	-1.0314925	0.001615966	0.014567207
LOC112846147	150.8603103	-1.032430263	0.00204958	0.017404112
rpl11	16139.97805	-1.03295729	1.97E-05	0.00049448
LOC100710903	836.5132963	-1.033207172	6.06E-08	4.98E-06

gpx8	246.5359748	-1.033219406	1.86E-05	0.000474535
rps11	13945.69835	-1.035063572	1.52E-07	9.99E-06
wasf1	128.0301293	-1.036232063	0.002330409	0.019171243
LOC102077132	138.8132698	-1.036489797	0.001932215	0.016689594
eya1	438.4534594	-1.039048997	0.000792489	0.008611197
LOC100695542	2155.985911	-1.039084072	0.00045642	0.005629687
LOC100705338	36.24386557	-1.040130273	0.005993361	0.038979911
LOC100691538	242.9181162	-1.040277279	7.00E-05	0.001327788
rplp0	29963.48781	-1.040469634	0.000155104	0.002464164
LOC112846510	29.4748127	-1.040999953	0.000680689	0.007693333
LOC100709875	238.5528919	-1.04155986	0.000855273	0.009120951
ybx1	164794.6905	-1.04191433	1.05E-05	0.000312254
LOC102077389	121.3191417	-1.043277229	6.01E-06	0.000202767
ddb2	93.47044501	-1.044478777	0.005090512	0.034246267
gse1	245.3703009	-1.045435522	0.000347144	0.004564613
tppp	74.44119809	-1.046541811	0.004641139	0.031816624
LOC100710334	182.5434429	-1.047691026	0.000497146	0.006031949
LOC100696855	472.4790416	-1.048141103	0.000630615	0.007240187
ndufaf8	385.4027187	-1.048302865	0.00091209	0.009567681
mep1b	72.71197358	-1.048598384	0.001141842	0.011235867
LOC112843274	51.45839339	-1.048839441	0.00796135	0.047922788
LOC100690685	52.37660382	-1.049516314	0.001480545	0.013678118
ppm1l	4310.65842	-1.050293865	2.10E-08	2.04E-06
pou6f1	48.52218472	-1.050544399	0.000906682	0.009527076
LOC100701444	79.92824813	-1.051192136	0.000141147	0.002299428
LOC100691566	168.444529	-1.052263083	0.000699956	0.007856752
dzank1	57.49526903	-1.053389411	0.002625001	0.020932441
rpl39	8843.829451	-1.05387029	8.74E-05	0.001578114
plagl2	105.9910188	-1.054622761	7.83E-05	0.001454756
macrod1	2166.598414	-1.055377734	3.02E-06	0.000118186
aldh4a1	2808.440638	-1.056726304	0.005154047	0.03454866
hoxd10	338.3996899	-1.058513628	0.001483166	0.013695522
psme1	1000.275936	-1.059949702	0.006448587	0.041036062
rp9	190.8212952	-1.059980608	7.55E-06	0.000243632
jag1	265.0824119	-1.060566215	0.001514699	0.013910689
aamdc	5365.330147	-1.06198135	0.000433723	0.005410763
phf2	242.9275877	-1.063187944	2.29E-05	0.00055713
LOC112847068	63.65731735	-1.063512293	0.006382354	0.040754102
LOC100689869	4638.85451	-1.064280398	7.00E-08	5.66E-06
LOC100702212	1068.847947	-1.064380587	2.33E-07	1.44E-05
rpl4	38113.11295	-1.064410266	2.29E-06	9.42E-05
sowahd	8501.943452	-1.066159322	7.11E-05	0.001342925
fkbp3	1605.367665	-1.066253996	3.18E-06	0.000123642
d2hgdh	654.5722897	-1.067107316	1.84E-06	7.93E-05
slc16a4	218.1031993	-1.069669703	0.000331171	0.004419105
megf10	126.8555483	-1.06984227	0.001181359	0.011520386
LOC100700180	138.0038725	-1.069985528	0.002028592	0.017273231
LOC100690136	46.92977407	-1.070014895	0.002816853	0.02208337

LOC112847414	68.12391858	-1.070979259	6.46E-05	0.001240411
LOC100695418	1029.64858	-1.071035479	1.21E-09	1.82E-07
pebp1	1147.12638	-1.071582593	5.92E-05	0.001155267
LOC102083312	8899.508406	-1.072420691	2.94E-05	0.000664185
LOC100701683	1287.208548	-1.072960941	0.004556894	0.031429884
dna2	83.92327038	-1.073425482	0.000539951	0.006417272
LOC100708525	13488.55476	-1.074710981	1.02E-07	7.39E-06
LOC100702127	1658.29712	-1.07749284	0.002010303	0.017148926
znf414	339.6746292	-1.077614605	3.60E-07	2.07E-05
LOC102080083	81.71794751	-1.077934179	0.001212278	0.011736362
LOC106096996	133354.0924	-1.078123842	6.97E-11	1.71E-08
LOC100709988	269.4665872	-1.081982951	0.00052251	0.006262039
LOC100707253	3277.852195	-1.082463122	9.09E-05	0.001628717
LOC100707633	68.50738447	-1.082525351	0.005623737	0.037068803
znf652	168.0997601	-1.082578471	0.000118991	0.002010737
LOC100692503	267.6865843	-1.083168062	0.000175671	0.002711666
LOC102076618	310.2033265	-1.083382576	0.00039753	0.005047332
sstr3	651.8431746	-1.084328082	2.06E-06	8.69E-05
dhrs3	53.53079952	-1.085498184	0.005400352	0.035864119
LOC100699764	9869.06579	-1.085912797	1.13E-06	5.29E-05
LOC102078473	121.3459662	-1.085965538	0.000380775	0.004920519
ick	324.36596	-1.087637237	5.07E-07	2.78E-05
col16a1	803.3879562	-1.087883456	0.000330983	0.004419105
LOC100697737	143.055845	-1.087935304	0.000542184	0.006435574
LOC100692969	517.9374936	-1.088238422	1.81E-05	0.000467967
olfml3	27.47127495	-1.089345701	0.005098578	0.034288119
jph1	25080.96884	-1.09222497	0.000202221	0.00302774
dcn	2960.516603	-1.094059013	0.002413998	0.019633747
LOC100700772	79.23946367	-1.094107154	0.00020163	0.003022503
LOC109201199	480.9058432	-1.095751516	0.000626867	0.007201608
itgb1bp1	542.8541605	-1.095825132	0.000221974	0.003264288
LOC100711350	35.07981118	-1.096884975	0.001204822	0.011676347
LOC100709414	136.6213966	-1.097682356	0.000336479	0.004470677
LOC100712287	34322.84448	-1.098149636	5.60E-07	3.02E-05
LOC100704944	59.47192903	-1.098414134	0.001913011	0.016565503
rfng	764.1656559	-1.098986354	8.37E-10	1.38E-07
rpl7a	35842.13116	-1.09976836	2.48E-06	0.000101156
sod3	164.9557804	-1.102441984	0.003658084	0.026918632
LOC100696434	733.6851264	-1.10345444	2.55E-11	6.68E-09
LOC100705212	68.26114027	-1.103576463	0.005838824	0.038228973
map3k2	994.0107631	-1.104277685	1.74E-07	1.12E-05
LOC100692468	405.6005323	-1.104638946	0.00011371	0.00193735
fam181b	16.77051912	-1.105298468	0.007719124	0.046905221
LOC102083291	235.1077656	-1.108649864	0.002081487	0.017588876
LOC100699040	416.1794137	-1.110428605	0.00261117	0.020857939
LOC100690483	3290.658713	-1.110894971	5.73E-05	0.001121644
sugct	136.0847164	-1.111264769	0.001816765	0.015944297
actn3	268490.8377	-1.111283122	6.86E-09	7.97E-07

ypel1	479.5449959	-1.111789945	0.002466166	0.019936999
uba52	8492.369315	-1.113497697	1.68E-07	1.09E-05
sertad4	66.41858902	-1.113786913	0.002350126	0.019264233
LOC100698351	104.8959366	-1.113884527	1.05E-05	0.000312254
LOC102079384	98.53398407	-1.113893855	0.000494948	0.006013133
LOC100693470	173.4067553	-1.114043425	8.39E-05	0.001532802
LOC112847887	34.47719226	-1.115442735	0.004047317	0.02901332
ahcy	739.5746015	-1.115926814	0.000340075	0.00449916
LOC100695257	214717.2527	-1.117022961	5.86E-05	0.001146152
lgalsl	38.10764749	-1.117951632	0.003078808	0.023704775
vav3	197.0940658	-1.118709357	0.004498907	0.031110747
LOC100711611	465.9189011	-1.118808141	0.000263358	0.003717012
LOC100710258	37.35804006	-1.118993107	0.004332717	0.030414101
plcb4	428.9524543	-1.120612606	3.21E-05	0.000702915
prkcq	706.4951638	-1.120720344	2.35E-08	2.23E-06
LOC100698155	726.0017371	-1.122276771	0.002065152	0.017488401
runx3	670.2787436	-1.122553021	2.98E-06	0.000117193
LOC100707311	15862.09958	-1.122847656	6.78E-07	3.51E-05
LOC102075970	18.50794477	-1.123363926	0.008086875	0.048479686
LOC100703285	874.2154787	-1.124623472	2.54E-05	0.000604844
LOC102075971	47.70313582	-1.125217796	0.001548414	0.014115707
LOC102075798	1302.042919	-1.126057117	2.38E-06	9.76E-05
LOC102081773	336.7693556	-1.126566669	2.45E-07	1.50E-05
dgki	330.5488082	-1.127311669	0.001398261	0.013120081
LOC102078726	55.38173569	-1.128245398	0.000556097	0.006550532
cmc4	468.2988522	-1.129607139	2.50E-12	9.33E-10
togaram1	287.3376745	-1.129749408	0.000815023	0.008792602
fam167b	85.28464596	-1.13068626	1.97E-05	0.000494049
cask	235.2816964	-1.131760926	1.52E-05	0.000412752
aldh2	16546.52346	-1.132854598	2.04E-05	0.000504833
LOC112848042	37.6978591	-1.133920174	0.001989981	0.017022439
fuom	118.4902893	-1.133984848	0.000114621	0.001947514
LOC100705018	24.75659912	-1.134067729	0.00206192	0.017468992
myoz1	126245.3697	-1.134939638	0.000934271	0.009745362
LOC100691655	337.0577122	-1.137187835	3.49E-05	0.000748804
LOC100693649	3616.812195	-1.139743961	2.84E-06	0.000112856
fermt3	362.9735009	-1.142515604	1.06E-06	5.06E-05
LOC100689912	165.8394805	-1.144157491	3.10E-05	0.000690193
arl4c	156.3235048	-1.145275913	0.006916587	0.043365304
LOC102083018	163.643619	-1.148472759	2.32E-08	2.22E-06
LOC100701152	62.90037631	-1.152105877	0.001509084	0.013872824
rnf207	923.8168366	-1.154786315	5.10E-05	0.001020617
LOC100706150	2037.162406	-1.15492442	0.000561327	0.006587091
tgfbi	715.9546002	-1.156909107	0.004642064	0.031816624
LOC100693678	6078.968005	-1.157391365	0.000125504	0.002090391
rpl5	23619.51507	-1.157642309	1.56E-05	0.000419675
alkbh8	451.5128212	-1.159968431	1.05E-06	5.04E-05
LOC100707421	562221.7516	-1.161758287	0.004806869	0.032764973

sema3b	311.1097175	-1.164849929	4.20E-07	2.34E-05
rpl29	6994.604818	-1.165828265	6.84E-06	0.000224631
LOC112846967	44.64216179	-1.168718753	0.001740248	0.015455199
naga	522.8090152	-1.169017792	0.000703933	0.007891861
map2k4	2302.912795	-1.169377666	2.31E-11	6.41E-09
ryr3	41778.1098	-1.170523105	0.000101457	0.001765811
LOC100705861	5739.65402	-1.172311129	1.56E-05	0.000419675
LOC100702396	270654.6637	-1.172368445	2.65E-05	0.000618592
tmem206	15.91373834	-1.1732369	0.004502921	0.031126925
LOC100690054	618.7235463	-1.173839615	0.000130678	0.002157231
LOC100696125	12904.28149	-1.174158915	0.004385562	0.030602995
LOC100691739	2149.304628	-1.174660869	6.27E-05	0.001208393
irx5	137.005669	-1.175208901	0.00567246	0.037337
LOC100703185	166.7055539	-1.176627214	8.20E-07	4.09E-05
LOC102081391	30.19332008	-1.177021056	0.004342511	0.030440381
rps12	9802.594228	-1.181780468	6.37E-05	0.001226154
LOC100708253	3485.014839	-1.182519381	1.51E-05	0.000411818
LOC100692412	73.22887134	-1.183032239	0.004586572	0.031552625
LOC100708980	1724.104815	-1.183590653	1.98E-05	0.000497241
LOC100699563	50.37715	-1.183904901	0.008419526	0.049809724
ica1	23.55292109	-1.184113926	0.001617479	0.01457378
lg10h11orf54	238.1293034	-1.184778942	1.73E-06	7.56E-05
LOC100692856	190.4646608	-1.186227917	3.56E-11	9.20E-09
ctdspl	1722.048857	-1.186423327	3.08E-07	1.85E-05
znf512b	595.1581285	-1.186702234	1.44E-07	9.59E-06
bhlhe40	2856.471858	-1.186843429	0.000177018	0.002726106
LOC100698093	157.3766535	-1.189240179	2.18E-10	4.46E-08
LOC100702938	1011658.009	-1.189289887	0.000171456	0.002662511
LOC100704458	595.4119363	-1.189642324	2.32E-05	0.000560782
amacr	280.2081393	-1.189865119	2.81E-05	0.000641819
LOC100702998	104.3860675	-1.189990344	4.76E-07	2.63E-05
LOC100690808	3517.924876	-1.19298312	3.97E-05	0.000824895
plekhh3	487.4500991	-1.193697323	1.83E-05	0.000470308
LOC100694078	6735.531285	-1.194158078	2.98E-09	3.88E-07
synpo2	14207.68981	-1.196732853	2.25E-06	9.33E-05
lnpk	1903.785851	-1.197091764	1.05E-09	1.64E-07
LOC100695408	2412.241171	-1.197622409	0.000951276	0.009861857
cacng6	1452.801059	-1.19802572	0.0011648	0.011413445
col6a1	4208.067535	-1.200088905	0.00064702	0.007401114
LOC100709052	155.1451845	-1.200329762	9.41E-05	0.001669687
LOC100708152	219.5066342	-1.201636157	0.000189034	0.002870721
LOC100691361	1753.593767	-1.202966721	6.11E-05	0.001184032
samd12	85.23893174	-1.205220807	0.003762422	0.027544662
LOC102082625	156.2821167	-1.205527917	3.32E-05	0.000720069
LOC100711355	88.89847032	-1.20640976	0.000180844	0.002766683
pkdcc	226.9270886	-1.207050415	0.007811703	0.04732853
slx4ip	89.6385652	-1.207553511	0.000144011	0.00233585
LOC100707753	2064.19793	-1.207593044	2.35E-08	2.23E-06

pgk1	67446.33238	-1.207813178	0.001390081	0.013089579
LOC100704899	36.86163014	-1.208114193	0.001776034	0.015668212
rpl15	11038.0567	-1.214868959	3.86E-06	0.000143771
nat14	87.48601874	-1.215035772	0.000135889	0.002227429
LOC102081844	35.77753293	-1.21606415	0.001751776	0.015505723
LOC100693076	873.1580797	-1.217489245	0.001910005	0.016556698
znf703	90.95292822	-1.217847757	0.000142873	0.002321433
nlrc5	18.56484823	-1.218872542	0.001869729	0.016293732
LOC100712142	118.2968566	-1.219339839	0.000552184	0.006525108
cab39	722.2448303	-1.220010002	4.81E-08	4.12E-06
LOC102077826	57.39278975	-1.220203277	0.001335958	0.012691426
LOC100704176	42.15880772	-1.220971812	0.00208364	0.017588876
trmt1l	169.9935386	-1.223630475	1.00E-05	0.000303224
LOC100708522	2388.350499	-1.22534442	6.21E-06	0.000207882
LOC100696374	217.512624	-1.226532066	0.000164863	0.00258169
amt	80.71022564	-1.229341874	0.000574854	0.006720364
LOC100705697	3652.163381	-1.230621758	0.005214731	0.034842348
LOC106098463	9772.434964	-1.230775141	5.14E-07	2.80E-05
park7	6297.11546	-1.231217023	4.27E-07	2.37E-05
marcks	146.943071	-1.232523122	0.002667542	0.021181284
LOC100702204	13987.51922	-1.23377734	1.56E-05	0.000419675
chac1	17638.5836	-1.233849772	0.000996861	0.010164373
LOC100703791	2225.404458	-1.236552976	1.61E-08	1.63E-06
LOC100699889	651.4900593	-1.236640673	0.000106458	0.00184079
rtn1	5500.912147	-1.237013282	3.40E-05	0.00073286
asb15	1697.54991	-1.237161887	0.000519442	0.006237328
agl	16786.67096	-1.238454003	0.000360356	0.004713789
LOC100701540	281.7285185	-1.239153822	8.90E-13	3.85E-10
map3k4	680.0013417	-1.240534372	3.61E-07	2.07E-05
txndc16	112.7494656	-1.24098478	4.86E-06	0.000174076
LOC100690919	1033.07516	-1.242609439	0.000101131	0.001761784
hsf2	206.356349	-1.244160232	2.74E-05	0.000635935
fsd2	3337.684382	-1.244375577	1.31E-07	9.05E-06
LOC100703371	585.5498461	-1.244390774	0.000660972	0.007509876
LOC100691426	33.45661153	-1.247436657	0.001298652	0.012430149
LOC102082462	30112.56322	-1.247611921	0.000123499	0.002062532
LOC106096953	16262.67913	-1.247836724	0.000136876	0.00223962
LOC100707723	664.1196209	-1.24931986	0.000444323	0.005524462
LOC102078050	67.27294425	-1.250389505	0.000229417	0.003342531
heca	157.1027463	-1.2505689	6.06E-05	0.001178813
tead3	1176.879245	-1.253246115	1.64E-07	1.07E-05
gsn	3766.291304	-1.253546347	7.29E-10	1.24E-07
aunip	470.078298	-1.253591378	9.07E-08	6.94E-06
lmf2	205.8048	-1.256143428	1.17E-06	5.43E-05
jmjd7	168.0343287	-1.256609197	2.01E-06	8.52E-05
LOC100700290	1324.747815	-1.25703868	0.000919177	0.009636583
LOC102079281	10155.34266	-1.257985229	2.79E-05	0.000641819
pcgf2	301.5380766	-1.258559919	7.04E-06	0.000230101

mettl18	136.4881438	-1.259002656	0.000687011	0.007739494
tril	28.92479093	-1.262237361	0.002758223	0.021759591
LOC100709577	143.5018346	-1.268128052	0.003411098	0.025628737
LOC100711089	366.0731549	-1.269268505	0.001193187	0.011587298
cttnbp2	2270.838845	-1.269632209	0.002206035	0.018379997
znf423	234.5361782	-1.270357741	7.35E-05	0.001379436
LOC100689838	64.12228363	-1.271593282	0.001887048	0.016428412
LOC106096580	36.12229758	-1.273207051	4.39E-05	0.000897001
ak1	157626.7225	-1.280559983	3.19E-05	0.000701901
mms19	264.7544902	-1.281262015	1.43E-07	9.59E-06
nt5c3a	6615.079264	-1.281441686	8.85E-08	6.83E-06
LOC100709415	46.37292887	-1.28394483	0.001073788	0.010771824
plekha5	414.4619491	-1.288084638	5.41E-07	2.92E-05
LOC100709899	38.45044794	-1.288331754	0.003071564	0.023690549
LOC100700997	568.6124404	-1.291521122	0.000387909	0.004989934
LOC100704840	68211.23426	-1.291873157	0.001606502	0.014538298
LOC100692377	971.7977325	-1.292234301	7.39E-07	3.77E-05
LOC100699761	273.0686718	-1.292668219	0.000947207	0.009841635
capza2	15708.00051	-1.293725506	1.05E-05	0.00031212
LOC100696383	147.0393179	-1.293839439	0.000179229	0.002753319
LOC100699180	4880.304641	-1.294065208	6.77E-10	1.18E-07
mmp2	782.40736	-1.294509429	0.001072471	0.010771824
chrdl2	175.7460598	-1.295741876	1.87E-05	0.000477111
hacd1	9491.74454	-1.295988433	9.70E-05	0.001708827
LOC112842619	122.2820447	-1.296510968	1.88E-05	0.000477724
LOC100691083	422.0200836	-1.296649051	0.000252626	0.003592819
LOC100690032	328.3062822	-1.299579545	0.001720592	0.015312602
kif13a	824.0569452	-1.300485435	3.23E-06	0.000124734
tkfc	54.75264991	-1.301411283	0.00127339	0.012232446
LOC100706210	1591.495122	-1.302229993	0.00470786	0.032184518
LOC100693645	53.12840014	-1.303262614	0.001914283	0.016565503
LOC102079249	97.0541048	-1.305029663	9.43E-05	0.001671094
swap70	46.52118125	-1.306772715	0.001612356	0.01455584
LOC109195198	403.9149396	-1.308025368	7.12E-11	1.72E-08
LOC100690323	69.80341912	-1.30873015	0.007238318	0.044759102
LOC100710803	1417.813099	-1.309158505	0.000921659	0.009657157
LOC100695895	109.956119	-1.310465532	2.79E-06	0.000111665
zcchc14	250.7656402	-1.310654859	9.81E-07	4.75E-05
tmem98	2646.232829	-1.313932774	1.77E-06	7.69E-05
LOC102076524	21.65101642	-1.315333979	0.001794755	0.015780938
hsd11b2	3647.110078	-1.316090899	0.002913277	0.022657737
LOC109197793	16.16587409	-1.316807732	0.005869576	0.038376248
LOC100699552	90.00540181	-1.318205722	1.91E-05	0.000484523
dhdh	591.6413684	-1.318770627	1.11E-06	5.23E-05
LOC100712379	457.5225745	-1.320702527	3.88E-06	0.000143787
LOC102082741	289.5514312	-1.321856297	0.007296967	0.044972153
mrtfa	3231.412851	-1.323707873	7.95E-10	1.32E-07
LOC100705578	33.09155241	-1.323742684	0.000774936	0.008483216

eef2k	18187.24823	-1.325708117	2.51E-05	0.000598165
LOC100707304	29.36231309	-1.325908419	0.001957546	0.016837974
rab30	393.8120355	-1.327452252	0.000104163	0.001806146
LOC100705511	131.609102	-1.328171759	0.001463785	0.013577266
prss12	50.4988046	-1.329625782	2.10E-05	0.000517154
LOC100700514	3123.406674	-1.330618396	0.000288823	0.003966223
LOC100712125	45.27416322	-1.330683101	1.78E-05	0.000462568
LOC112847360	88.1321789	-1.331261569	0.000232956	0.00338031
myod1	4061.400759	-1.33139726	4.69E-08	4.04E-06
cap2	4971.826983	-1.331667769	4.44E-12	1.50E-09
LOC100693321	63.73052173	-1.331758008	0.00042533	0.005334711
kdm6a	628.2731081	-1.333099817	7.37E-08	5.86E-06
ppid	399.324473	-1.334109445	0.006355878	0.040667232
crtc1	296.4144836	-1.33463019	8.64E-13	3.83E-10
LOC100700547	334.4639294	-1.337848013	2.11E-06	8.85E-05
LOC100703165	89.25296018	-1.338307516	0.000347233	0.004564613
LOC100693232	7729.049711	-1.339129369	6.26E-06	0.000209299
LOC100698578	970.0564542	-1.340511445	2.51E-11	6.67E-09
mast3	81.60921644	-1.341465968	0.002521461	0.020313777
LOC100697747	15604.11365	-1.343525282	8.08E-16	7.90E-13
ccdc112	24.43026857	-1.345415272	0.004602105	0.031614653
LOC112842578	458.9713032	-1.34657284	0.005288069	0.035218429
LOC100690328	20.30314738	-1.347330833	0.002280659	0.018866441
tmem38a	21770.77595	-1.347647395	1.49E-05	0.000407152
LOC100698614	34.57323212	-1.347789316	1.75E-05	0.000456604
tnk2	355.5315311	-1.349259425	1.07E-05	0.000314023
sema6d	149.8182231	-1.350669173	0.000236621	0.003420155
nccrp1	271.469553	-1.351241695	1.85E-05	0.000474535
LOC100692963	21.14230161	-1.356088916	0.003832619	0.027861055
LOC106098155	23.31755633	-1.358984729	0.002808062	0.022051654
p4ha3	76.51027638	-1.360235661	8.44E-09	9.40E-07
LOC100711726	37.6839189	-1.362547872	0.00075606	0.008315767
sparc	8396.379644	-1.363224312	0.000828459	0.008911685
LOC100709148	82332.94152	-1.364673219	9.75E-06	0.000295528
pabpc4	105959.3888	-1.364900465	1.50E-10	3.33E-08
sobp	309.5096086	-1.366596806	0.003447084	0.025834731
LOC100695823	969.8384046	-1.36797637	0.000189298	0.002872377
LOC100700182	75.46547089	-1.368700131	0.002630676	0.020968697
vwde	36.82280453	-1.36977254	0.000169865	0.002642214
frem3	1171.486758	-1.369791531	0.004895567	0.033211244
LOC100711526	295.9997827	-1.370056668	2.60E-05	0.000612731
LOC100694084	648.0370484	-1.37113983	1.86E-05	0.000474535
LOC100707848	9116.490554	-1.372712742	3.60E-06	0.000136944
LOC100712394	54.36420022	-1.372759287	0.000557699	0.006561078
LOC100708774	99.00740061	-1.373447406	0.000355962	0.004669459
LOC100692786	6506.85469	-1.377926161	5.10E-08	4.29E-06
LOC100707233	10408.22891	-1.378322664	0.00013731	0.002242815
LOC102078469	58.7094444	-1.378401944	0.000987803	0.010088617

LOC106098444	109.0494694	-1.378712206	3.19E-06	0.000123927
nkpd1	354.6668517	-1.381019203	1.01E-08	1.09E-06
LOC100706175	11.6114532	-1.382790491	0.004602391	0.031614653
LOC100703531	655.5345625	-1.383414106	0.00058735	0.006857826
LOC100707522	60439.94057	-1.384246394	2.23E-06	9.31E-05
LOC106096657	41.53994341	-1.384742418	0.003078022	0.023704775
LOC100703707	25547.36391	-1.385036226	5.63E-06	0.000193035
LOC102082068	1102.474655	-1.385913335	0.000285138	0.003923133
LOC100697478	934.9862236	-1.386347491	4.34E-10	8.23E-08
LOC102078207	440.1869338	-1.387256977	6.43E-06	0.000213307
LOC109195775	442.0326492	-1.387427959	1.95E-06	8.32E-05
LOC100711663	136.1537307	-1.387958698	0.000276936	0.003844423
LOC100707163	7207.558727	-1.389774689	2.89E-06	0.000114423
LOC112841976	505.9609643	-1.390097199	2.36E-05	0.000569091
LOC100694920	2004.849454	-1.391104338	0.000654461	0.007454119
LOC100712370	100.9075942	-1.391795443	0.007083291	0.044005418
phex	1056.038938	-1.392994162	9.22E-05	0.001646987
LOC100695463	17061.84231	-1.395663339	1.16E-09	1.75E-07
LOC100692260	5814.518055	-1.398980541	0.004620182	0.031701714
LOC100708189	2950.634901	-1.400419867	0.000247556	0.003547952
LOC100700643	29.14462267	-1.401910775	0.003129625	0.023969292
LOC100709626	3355.222123	-1.402083396	1.69E-05	0.000445493
LOC102078966	63.57462843	-1.402289111	9.50E-06	0.000289951
srpx	89.6085828	-1.404787705	0.000633841	0.007268257
LOC100711198	196374.0194	-1.409941661	0.000822823	0.008866454
LOC100698682	255.5423887	-1.410108104	0.001156007	0.011345223
LOC100702644	120.4008005	-1.412023438	3.61E-06	0.000136984
LOC100691333	657.0131974	-1.413113515	3.03E-08	2.75E-06
LOC102080766	156.7024467	-1.414164864	3.08E-06	0.000120127
LOC100708022	180.1037879	-1.417272901	7.61E-06	0.000245045
LOC100697868	951.0707888	-1.417886609	1.05E-13	5.43E-11
LOC112846754	58.44699116	-1.41833637	4.08E-05	0.000844281
LOC100709393	56.87752872	-1.419466946	1.23E-05	0.000350922
klhdc8b	222.8304069	-1.419941503	0.000377389	0.004881634
LOC100692929	74.62229235	-1.421729734	4.69E-06	0.000169257
LOC100709002	1297.735147	-1.421743685	0.000119288	0.002013228
ftcdnl1	91.85336257	-1.425098827	1.93E-05	0.000487642
LOC100711840	32.2611921	-1.425135329	0.00048369	0.00589563
LOC100699708	216.2364035	-1.427383126	0.000269616	0.003779505
LOC100695146	1155.164884	-1.428222445	2.81E-05	0.000641819
ald	535664.5159	-1.432479808	0.000434942	0.005418705
LOC100693268	10.2416868	-1.434463373	0.007935964	0.047816436
LOC100711942	21.97458281	-1.437335567	0.000338122	0.004485784
LOC100693149	1055.651652	-1.437935665	1.44E-07	9.59E-06
LOC100698220	330.6001579	-1.443217767	7.66E-08	6.03E-06
gsta	441.2744303	-1.445205061	2.64E-05	0.000617839
LOC100709319	578.285229	-1.448027956	8.56E-07	4.24E-05
fgf14	265.2687842	-1.449416392	1.92E-05	0.000485614

esd	1202.823503	-1.449698446	3.17E-07	1.88E-05
lztfl1	18.11202884	-1.450538504	0.001081789	0.010834212
LOC100698303	83473.4077	-1.451083767	0.00024088	0.003473611
tet3	337.0196985	-1.452445073	4.97E-08	4.20E-06
LOC102081988	23.07999981	-1.457003093	0.001528013	0.013987952
LOC100689834	409.4978438	-1.458287392	0.001018993	0.010333359
LOC100692384	490.7949143	-1.458435326	5.20E-05	0.001037017
LOC102082350	563.6866379	-1.459075429	7.60E-08	6.01E-06
dcxr	304.0817784	-1.459176832	3.52E-09	4.42E-07
LOC100691117	11518.17064	-1.460073219	0.000924044	0.009665798
LOC100705548	110.9339653	-1.462419192	4.00E-07	2.27E-05
LOC112841795	359.7439804	-1.465364976	8.74E-05	0.001578114
LOC100696148	277.8774396	-1.465494114	0.002963858	0.023003001
LOC112844142	26.64306075	-1.466325379	0.004586469	0.031552625
LOC106096429	80.00764253	-1.467956424	0.000744279	0.008230014
LOC100702616	46.19535969	-1.475101501	0.003475253	0.025971045
LOC100694851	38566.60162	-1.475816951	5.66E-10	1.01E-07
ansn	208.0865303	-1.477301313	8.71E-06	0.00027341
LOC102080648	109.7071579	-1.479177462	5.29E-06	0.000183813
lg16h2orf40	207.1362623	-1.482353585	8.21E-07	4.09E-05
frzb	566.5836752	-1.482507912	1.12E-07	8.03E-06
LOC100693920	2915.893693	-1.485753717	0.002052069	0.017417288
LOC100703178	604.7006736	-1.486694525	2.77E-08	2.55E-06
LOC100701242	44.76737957	-1.488431041	0.003002085	0.023244741
sema3e	21.91190736	-1.489509102	0.001800369	0.01581534
LOC100696522	37.21748537	-1.49067327	0.005138238	0.034504902
LOC109201885	13.04682352	-1.49115323	0.007611538	0.04643363
LOC102080300	118.8824509	-1.491466416	0.004400259	0.030656677
dusp10	2365.023292	-1.495437637	9.17E-06	0.000283185
cnksr2	238.6585309	-1.495764638	0.003578897	0.026493244
LOC100706964	2851.05428	-1.49596546	2.75E-16	3.20E-13
LOC100712433	10614.4604	-1.497544607	2.86E-06	0.000113486
LOC100698358	41.42939739	-1.497867717	0.003554998	0.026358317
tmod4	40379.82857	-1.497903686	1.50E-05	0.000407722
LOC100699677	290903.2211	-1.499214155	0.001931196	0.016688551
dock11	417.564964	-1.499438864	1.56E-06	6.93E-05
LOC100691499	126.9145829	-1.501975211	8.24E-05	0.001514191
LOC100691614	134.3416581	-1.504188695	1.11E-05	0.000321269
LOC102076448	2104.99985	-1.504310107	0.000613866	0.007091696
LOC100711891	75167.63242	-1.508073799	0.00018545	0.002821322
phc1	129.4589268	-1.508191628	1.87E-07	1.19E-05
LOC100693266	4362.244523	-1.508571135	6.98E-07	3.60E-05
LOC100710725	246.2767386	-1.509349141	3.31E-06	0.000127227
tpi	73336.98785	-1.511092862	0.00281588	0.02208337
LOC100704310	1471.877136	-1.512380126	0.000636467	0.007293864
hsd17b12	20.05903089	-1.513109088	0.001383714	0.013036222
sox11	20.39031784	-1.51327358	0.003673135	0.027008
LOC100702784	128.5256406	-1.517308908	0.000532519	0.006353078

LOC100701307	48.96555486	-1.517638467	6.88E-06	0.000225661
tmem131l	399.0872757	-1.51876265	1.13E-09	1.72E-07
LOC100703845	21823.32667	-1.520862708	0.000826181	0.008892328
LOC102080881	564.0722006	-1.521442528	3.31E-05	0.000718859
slc5a11	15.41845761	-1.527898116	0.004463156	0.030921158
LOC100712281	232.4899648	-1.528172191	4.78E-10	8.98E-08
colq	412.3244632	-1.533497368	2.61E-05	0.000613166
LOC100703211	970.4196119	-1.536961759	0.000685148	0.007723182
LOC100711322	21707.90488	-1.538021422	1.14E-06	5.31E-05
LOC100699370	134.1042156	-1.543221809	5.14E-06	0.000179333
mylpf	1158559.299	-1.545543463	9.86E-08	7.22E-06
creb5	109.5102959	-1.546188874	0.000227611	0.003325936
LOC100697965	7718.780792	-1.546923092	4.95E-05	0.000994979
LOC100698617	192.0499011	-1.551889186	0.000155667	0.002471001
hhat	36.6466342	-1.551922944	0.003592044	0.026569406
LOC100709203	2253.898651	-1.553090171	0.001342309	0.012730018
fgf13	177.7004823	-1.553526897	0.000980965	0.010057464
LOC100694050	11.08280567	-1.559142993	0.002669222	0.021185097
ripor2	234.1582264	-1.559866266	3.78E-08	3.40E-06
LOC112847383	18.20326222	-1.55991369	0.001138291	0.011218742
ttll1	20.82523488	-1.561697872	0.006128969	0.039557387
tpm1	642096.2487	-1.565332336	0.000837853	0.008991925
LOC100694217	1340.209484	-1.566962574	8.93E-07	4.38E-05
LOC100697818	995.3721561	-1.568095159	7.38E-10	1.25E-07
LOC112846274	156.4736461	-1.57001059	2.76E-05	0.000637296
LOC100704296	19089.91679	-1.570305042	0.000436172	0.005430389
dnajb5	53.42562626	-1.571615229	0.004088057	0.029215225
tnmd	179.9366506	-1.572658824	0.00015488	0.002462704
LOC100710932	15312.64175	-1.578688209	0.000119355	0.002013228
LOC100706194	1165.472007	-1.580507574	3.22E-06	0.000124664
LOC100697400	89.8528621	-1.580511864	0.000117817	0.001994523
asb2	115.0065637	-1.581404883	1.06E-07	7.66E-06
serpinh1	1229.01909	-1.584333381	0.000533752	0.006359855
LOC100698429	591848.992	-1.584936861	0.000209581	0.003111576
pcgf3	676.2539198	-1.585565462	5.44E-10	9.89E-08
tmem236	50.54554774	-1.587100589	2.26E-06	9.37E-05
LOC100709042	464.3447468	-1.588341267	1.25E-05	0.000355189
igsf10	88.76816931	-1.589914658	0.000295079	0.004028118
LOC100702666	10.5654077	-1.591685229	0.00153026	0.013998261
osr2	753.7260973	-1.592702193	0.003913698	0.028262559
LOC109204233	46.09965413	-1.593163064	0.000845041	0.00903663
st8sia1	100.8307572	-1.594337067	0.00387357	0.02807092
LOC100699182	56.602259	-1.595279687	0.000785042	0.008563597
LOC102080335	15081.66925	-1.596082909	4.01E-06	0.000147443
myc	401.4815154	-1.596166676	0.007038341	0.043815267
LOC100703685	5281.045175	-1.596587974	0.000158094	0.002498846
LOC100697399	109.6496676	-1.597668893	0.000158855	0.002504486
LOC102077971	151.8454421	-1.599957981	0.006909782	0.043351842

LOC100697755	146.6437171	-1.601148001	0.00041095	0.005175294
ucma	140.2019139	-1.602402616	3.35E-05	0.000724874
LOC100690586	1586.619846	-1.603025343	5.99E-06	0.00020252
LOC100700968	105.1889339	-1.605488262	0.000397337	0.005047332
slc25a10	2453.171086	-1.606476059	3.89E-09	4.85E-07
kctd15	150.5327492	-1.609168497	1.55E-08	1.60E-06
LOC100693044	141.6778574	-1.612783735	7.00E-07	3.61E-05
LOC100699058	225.107664	-1.615486801	9.11E-06	0.000281828
LOC100711636	76.87147659	-1.616696182	0.000150648	0.002416463
rpa2	358.3278861	-1.618431078	7.00E-08	5.66E-06
LOC100706575	1070.569791	-1.6196831	2.23E-05	0.000546311
vasn	29.66429065	-1.619987937	0.003631744	0.0267787
fam180b	72.13863547	-1.623910957	0.000380925	0.004920519
myom1	37621.23872	-1.625360776	0.000123313	0.00206128
ncapg	52.70185003	-1.627372251	0.000745675	0.008235649
LOC100702310	403.8804212	-1.631893398	1.10E-09	1.70E-07
LOC100702995	13.73441527	-1.634869485	0.006393684	0.040812428
LOC100703060	31.94173207	-1.635683352	0.004835851	0.032902195
LOC100691930	149.6691214	-1.63585489	0.002685896	0.021290163
LOC100699402	10.69363055	-1.636398906	0.007149025	0.044369311
gucy1b1	45.58303844	-1.637307891	0.002543397	0.020443531
LOC109199520	195.7193711	-1.639660996	0.003417854	0.025667378
myf5	91.9637081	-1.641037378	6.52E-08	5.32E-06
adgrb3	10.14716391	-1.645369076	0.00263829	0.021011371
enpp5	250.6189527	-1.646279001	8.35E-08	6.52E-06
ano8	1112.298303	-1.652085971	1.40E-07	9.49E-06
LOC100709730	746.4634856	-1.654926433	8.14E-05	0.001500854
LOC100696082	337.8504172	-1.656290804	8.77E-10	1.42E-07
neb	195185.958	-1.660832729	8.87E-10	1.42E-07
LOC100697881	476.4708027	-1.669830402	7.52E-10	1.26E-07
LOC102080773	673.0577624	-1.673602587	1.71E-07	1.11E-05
abi3bp	784.2666099	-1.680173129	3.15E-07	1.88E-05
LOC102082088	107.5023539	-1.681250323	0.002198957	0.018362178
LOC102078284	45.09712165	-1.682918577	4.05E-05	0.000839546
LOC100703741	96576.50961	-1.683674264	1.24E-06	5.69E-05
LOC102079001	34.37340344	-1.683886301	0.000144797	0.002346542
LOC100692768	16.74787772	-1.684753994	0.004023944	0.028914927
LOC102076799	58.99399139	-1.691538703	0.007040612	0.043815267
celsr1	304.6541309	-1.692843382	3.15E-06	0.0001229
LOC100705949	1107.191377	-1.693414619	2.39E-11	6.45E-09
evpl	124.4205189	-1.696489817	3.17E-14	2.03E-11
tmem97	15.22976511	-1.697955472	0.000737964	0.00818942
LOC102076318	14.43792174	-1.697981374	0.00800273	0.048074546
LOC100703220	48.55627559	-1.69868726	0.000754758	0.008311282
LOC100693445	832.2714889	-1.70302149	9.76E-05	0.001717689
LOC102077756	21.11268857	-1.704842488	0.00015316	0.002443723
LOC109198664	223.4802495	-1.70553061	6.23E-07	3.28E-05
mthfr	935.9469816	-1.707440904	4.08E-08	3.63E-06

smpdl3b	930.232914	-1.711541398	7.32E-05	0.001375008
spon2	220.0129508	-1.71587091	7.03E-07	3.61E-05
lrrc30	112.517691	-1.718767174	0.000222757	0.003273203
LOC100694213	17.87899613	-1.723583045	0.001995953	0.017065671
prickle2	24.82075476	-1.726606797	0.000298361	0.004068916
LOC100697345	425.858229	-1.727150047	0.000101588	0.001766435
lcat	140.0862272	-1.728619207	0.002042643	0.017365009
LOC100707533	634.7912787	-1.729431721	0.000225794	0.003307926
LOC106098477	25.97981667	-1.730779028	4.52E-05	0.000918852
LOC100706438	341.8371598	-1.73304158	1.07E-05	0.000314023
LOC102081732	17.46554256	-1.735539442	0.00033197	0.004423406
LOC100703212	118.2540127	-1.73684333	1.30E-08	1.36E-06
LOC109200664	81.12760287	-1.738258975	0.000679664	0.007693333
LOC100696396	40.96946357	-1.740716688	0.00057262	0.006702686
LOC100703573	221575.7498	-1.741647321	0.003793696	0.027751763
ky	7158.46386	-1.74182216	3.08E-05	0.000688836
LOC100703581	20.93215024	-1.741939248	0.000161319	0.002541182
cpxm2	126.2702991	-1.743129872	0.000900989	0.009472613
LOC112843227	37.22653368	-1.745815771	3.89E-05	0.000813117
LOC109201497	221.5523101	-1.747186338	0.000943034	0.00981473
LOC100705884	2883.410325	-1.747650223	2.59E-05	0.00061235
LOC100694933	152.2875506	-1.74827127	0.000450687	0.005573764
LOC109194449	11.77125329	-1.74917097	0.003229076	0.02456313
LOC112848226	93.11785676	-1.753591639	0.002124403	0.017876145
hemk1	412.4640469	-1.75415039	1.69E-10	3.69E-08
LOC112841638	82.73790418	-1.75783195	2.02E-06	8.57E-05
LOC100694932	36.47126146	-1.759129701	0.00185292	0.016169986
LOC109199175	143.6248205	-1.762959718	0.001174934	0.011488514
LOC100691876	125.0975694	-1.76300686	2.80E-19	6.50E-16
ercc5	953.9001926	-1.763991297	7.87E-05	0.001459017
LOC106098508	18.1069932	-1.76536896	0.004218366	0.029882233
LOC100709482	29.2154801	-1.769516735	0.001153291	0.011330533
LOC100712188	20.1921696	-1.77199139	0.000127026	0.002110063
LOC100709897	34.59741278	-1.773169351	0.003496667	0.026081881
fkbp1b	1202.687139	-1.778591591	1.24E-05	0.000351372
LOC102075830	313.4488129	-1.780552979	1.19E-15	1.11E-12
LOC102082492	112.4764741	-1.782624424	0.000984457	0.010075909
LOC100707898	735.6695983	-1.790232891	3.75E-05	0.000789142
LOC100710008	186.2084004	-1.792263295	0.00083881	0.008992019
LOC100701993	22.7479304	-1.796499424	0.003985017	0.028699537
LOC100697931	86.73208867	-1.797258424	1.47E-09	2.16E-07
setbp1	838.2377971	-1.797475947	6.14E-09	7.23E-07
LOC109195671	208.4163502	-1.798440258	3.68E-05	0.000777581
LOC100708475	26.1932461	-1.799700058	0.000100537	0.001754732
igfbp2	10.63181772	-1.80187948	0.007396111	0.045447577
LOC100711768	94.78867594	-1.803132358	2.72E-08	2.52E-06
LOC100707661	6.418147096	-1.803192753	0.006414084	0.040921266
mlf1	527.1327533	-1.80402684	0.003344068	0.025227088

LOC102077853	13.89216408	-1.806253428	0.003621783	0.026744122
gsdf	1230.292166	-1.806546136	8.55E-08	6.65E-06
e2f2	18.41496822	-1.810230515	0.005483663	0.036325847
pdlim5	8158.587397	-1.815850947	5.20E-09	6.28E-07
LOC100696185	412.682038	-1.816901788	0.001540795	0.014073854
LOC100709119	71.43151106	-1.818239607	5.06E-05	0.001014978
LOC100700844	60.31354585	-1.828423346	0.000126973	0.002110063
LOC100690019	1241.538843	-1.829840301	2.01E-05	0.000501402
LOC112846412	41.16893342	-1.830104926	0.006596136	0.041746334
LOC100704523	4227.492202	-1.831373955	1.91E-07	1.21E-05
egln3	3612.748632	-1.837396087	0.001641861	0.014729202
LOC100689761	125.8178433	-1.841346391	2.07E-10	4.33E-08
kcnk5	43.08412904	-1.841505364	0.00201166	0.01715263
cdc42ep3	132.5313972	-1.843087693	7.06E-06	0.000230301
LOC100698230	225.5207189	-1.843781829	5.42E-12	1.77E-09
LOC100703154	47.51453674	-1.844263821	2.48E-08	2.33E-06
LOC100711959	20.69140084	-1.845540774	0.000764819	0.008387289
cbx4	14.78612469	-1.850912846	0.000964287	0.009941298
rpl3l	28588.5327	-1.855476471	1.52E-09	2.20E-07
LOC100698560	453.3065432	-1.856408343	9.99E-07	4.82E-05
LOC100701404	13.63784851	-1.857580661	0.00723506	0.044753839
capn9	9.191335551	-1.861621155	0.002409019	0.019631236
LOC100710109	16.77148781	-1.862656717	0.006424769	0.040953634
LOC100701052	17.86403785	-1.866966361	2.33E-05	0.000563304
LOC112843315	13.48692936	-1.86882784	0.00607706	0.039360357
LOC102078622	75.48529389	-1.871897867	1.66E-05	0.000439547
LOC102082756	171.4598688	-1.872616208	1.16E-14	9.40E-12
LOC100700441	40.54548762	-1.872669735	6.08E-07	3.22E-05
LOC102078747	604.3490653	-1.873798093	4.28E-08	3.75E-06
npc1l1	10.47926842	-1.875649406	0.002977737	0.023092518
ube2ql1	82.03156218	-1.878433854	0.001947081	0.01677902
tec	6.737627474	-1.879602516	0.00727789	0.044899245
LOC112846879	49.57469904	-1.884383994	9.37E-08	7.07E-06
LOC109194623	15.2313853	-1.890394957	0.001225702	0.011841656
LOC109201916	5.026652614	-1.892625603	0.007255219	0.044833778
LOC100703810	46.30900316	-1.900109433	1.47E-07	9.76E-06
LOC100692912	1624.146651	-1.900793365	2.02E-05	0.000501411
gamt	9517.806137	-1.902285237	2.99E-06	0.00011725
LOC102081188	105.6194316	-1.903784456	0.002561422	0.02054886
fgf16	31.07794017	-1.905221693	1.19E-06	5.50E-05
LOC102078280	72.42406434	-1.90638017	1.10E-08	1.18E-06
LOC102080488	117.5857568	-1.911455948	1.32E-07	9.05E-06
LOC102079312	336.0783296	-1.916451388	1.37E-06	6.21E-05
chst11	20.26303267	-1.918997316	0.003386366	0.025473805
LOC106098048	26.03096851	-1.919341307	4.10E-05	0.00084645
LOC100711246	952.5953859	-1.920062661	1.01E-05	0.000303224
LOC100702001	15.8805119	-1.921896674	0.000623571	0.007180872
LOC100710615	43.51433828	-1.922271902	1.06E-05	0.000313685

tnfaip8l3	10.19280173	-1.923766627	0.003128882	0.023969292
tmem158	17.68284283	-1.925860906	0.008160671	0.04874375
pik3r1	11742.41753	-1.936609929	3.36E-06	0.000128747
LOC102081722	270.9824476	-1.939139551	1.14E-06	5.33E-05
LOC100694912	1046.335663	-1.944499936	1.59E-09	2.27E-07
LOC100694909	1470.089857	-1.94576089	2.67E-06	0.000108043
kcnq3	71.36702917	-1.950589283	0.000604032	0.007008579
eda	29.49751297	-1.952147637	1.70E-05	0.00044781
LOC112844847	11.21400623	-1.952466034	0.007021255	0.043795667
LOC100699682	183.3595008	-1.955738942	1.91E-07	1.21E-05
cited2	402.8176358	-1.96049828	0.000149719	0.002411867
rab34	485.4308236	-1.961952402	0.000167346	0.00261398
LOC102081727	262.4521637	-1.962085261	0.000559265	0.00657534
LOC100696479	85.76228044	-1.962304583	0.000571308	0.006691543
LOC102075836	50.80273165	-1.964187113	0.000150243	0.002415853
LOC100692021	9.05399388	-1.969135019	0.007890475	0.047588625
LOC100696318	12748.16597	-1.974922463	1.49E-09	2.19E-07
LOC100709438	10.87789076	-1.976062371	0.000524798	0.006285407
LOC100709683	1518.616327	-1.97937224	8.35E-14	4.70E-11
top2a	25.03608464	-1.982871518	0.000950995	0.009861857
LOC100712464	78.85192636	-1.983193574	0.000126143	0.002099141
LOC100697765	95.34014564	-1.983416429	5.78E-07	3.11E-05
LOC102077285	1256.13114	-1.988359845	0.000790709	0.008610255
LOC100701281	53.89675517	-1.99299434	0.000337516	0.004481249
nags	1951.010866	-1.99510852	2.42E-05	0.000581898
LOC102082989	30.65467373	-2.002383258	0.007608119	0.046428007
LOC102079381	6.127843016	-2.007253339	0.004338291	0.030440381
LOC100700843	3603.971441	-2.007278011	0.000333407	0.004439374
apex1	612.3967088	-2.007922193	1.60E-05	0.000427786
LOC100691279	131.0587011	-2.009254471	2.10E-06	8.84E-05
LOC100695765	52.92316751	-2.010101175	6.20E-05	0.001199189
LOC102078118	18.46771278	-2.010326413	0.000641945	0.007352112
LOC100693836	78.3872053	-2.012123841	0.000352081	0.004621803
LOC109200288	70.94803101	-2.015784884	5.69E-08	4.74E-06
LOC100696000	12.04982073	-2.017130333	0.000341347	0.004509561
LOC100693618	12.54451286	-2.017788348	0.000553905	0.006532981
LOC100703692	2135.351823	-2.018693893	7.05E-12	2.15E-09
LOC100699848	1081.553144	-2.022339221	7.16E-12	2.15E-09
pard3	40.66489249	-2.029363169	5.09E-07	2.78E-05
LOC102081909	130.76307	-2.031871888	1.52E-12	6.28E-10
LOC100711303	14.70625805	-2.034780625	0.000590318	0.006888152
LOC102078487	6.622861006	-2.043184597	0.002397395	0.019553653
LOC109197601	29.46445585	-2.043872777	5.89E-07	3.16E-05
LOC100700376	268.2801051	-2.045760672	0.00013614	0.002229576
prss35	74.02606381	-2.049837296	2.29E-05	0.000557238
LOC102082391	130.0614411	-2.054329325	2.59E-12	9.44E-10
LOC100709366	11950.57469	-2.055724903	2.73E-09	3.63E-07
LOC100534441	13.90740875	-2.06041891	0.000691701	0.00777348

LOC109199523	459.1272168	-2.063444544	0.000417176	0.005246597
LOC100704397	200.4768656	-2.06930573	3.24E-09	4.18E-07
LOC109200289	8.022444705	-2.06931188	0.002571288	0.020616531
LOC100697759	692.1934844	-2.071894906	0.000146856	0.002377834
LOC100711095	20.72616817	-2.075772301	1.16E-05	0.00033136
LOC100694532	24992.37199	-2.076083347	1.05E-05	0.00031212
LOC106097561	9.175976033	-2.084477793	0.00370903	0.027228848
LOC100710560	19.72187005	-2.085921538	0.00131457	0.012542046
dusp8	5134.234655	-2.090009163	1.14E-05	0.000328122
LOC100534413	2017651.332	-2.090593239	8.63E-05	0.001565037
neil3	278.926057	-2.097819247	0.000598404	0.006960661
LOC100704461	373.2890234	-2.097930549	0.000387724	0.004989934
LOC100693975	150.8314313	-2.103085566	0.001843936	0.016108278
aspm	7.288875655	-2.103182774	0.00580925	0.038089009
LOC100701596	266.226376	-2.110732238	7.17E-08	5.75E-06
LOC102081139	336.2643478	-2.111764449	7.26E-08	5.79E-06
LOC100709572	15.35767232	-2.113867093	0.000580906	0.006786857
LOC102076303	24.78597354	-2.116672885	4.89E-06	0.000174919
LOC109204625	7.859157252	-2.119695997	0.002131031	0.017907599
col1a2	22645.29816	-2.12244722	1.61E-05	0.000429544
LOC100704781	11008.91565	-2.12660661	0.000156696	0.002480374
zmat4	35.47737829	-2.126694267	0.000140901	0.002297429
gabra2	24.44171893	-2.128114665	0.001309542	0.012508612
LOC100693663	12.68545947	-2.128521848	0.002259574	0.018725355
tmeff2	181.3130652	-2.136352955	5.86E-10	1.04E-07
LOC100696012	2987.134661	-2.138044237	1.83E-07	1.17E-05
LOC100702909	7.843458445	-2.139749	0.003406696	0.025606012
LOC100701668	337.1617649	-2.146989935	2.54E-08	2.38E-06
LOC102076080	98.10910098	-2.147867834	6.10E-05	0.001183461
LOC100699358	13.24542168	-2.153129818	0.000184548	0.002811783
tmem179	238.2778528	-2.155249282	1.36E-08	1.41E-06
LOC100706950	1168.052091	-2.15908678	2.21E-10	4.48E-08
LOC112846035	22.18732645	-2.164843571	0.003590344	0.026567404
LOC100693924	28.1214912	-2.167176274	4.93E-06	0.00017535
LOC100710596	37.8206941	-2.17166553	1.37E-05	0.000377817
LOC109197059	104.9342833	-2.173432733	3.04E-05	0.000682645
LOC100696748	619.9512104	-2.176686095	6.49E-05	0.001244693
LOC102079261	42.45097976	-2.182913973	2.82E-05	0.000641819
LOC102078373	39.77539061	-2.19294086	0.000362201	0.004731261
LOC100705788	23.42133999	-2.192983597	1.67E-05	0.000441697
LOC106098801	79.53676672	-2.205534689	1.15E-06	5.35E-05
eno1	8824.08781	-2.207090187	1.81E-05	0.000466963
LOC109201227	17.75755066	-2.209387986	0.000843833	0.00903003
LOC102082692	20.92162825	-2.212828708	0.000312885	0.004229744
LOC109201441	53.52178689	-2.213688643	0.003124527	0.02395986
LOC100707162	240.8906871	-2.217221288	1.74E-08	1.74E-06
LOC102082790	27.08390673	-2.220828334	5.74E-05	0.001122748
rpgr	15.6720374	-2.239495744	3.15E-05	0.000697005

LOC109205049	17.88552024	-2.245803185	0.000300147	0.004084279
LOC106098723	53.84238631	-2.256342259	7.13E-07	3.65E-05
LOC100710865	2241.231056	-2.263357876	2.64E-09	3.56E-07
LOC102080315	81.9885824	-2.276175862	6.74E-07	3.50E-05
tp73	19.60504289	-2.277934283	5.01E-05	0.001006852
LOC100690648	13.08196837	-2.281729333	0.002139077	0.017949053
LOC100695604	29.03568428	-2.292203153	0.000267334	0.003753171
thsd7a	82.57535417	-2.309153382	4.40E-05	0.000898035
LOC100703878	8.630930443	-2.311359472	0.001118913	0.011104302
LOC100710771	27.62059817	-2.317772618	0.001198732	0.011629451
LOC100691819	7.29558163	-2.319495634	0.004327392	0.0303882
cish	260.6024693	-2.322730281	6.13E-06	0.000206408
LOC100690665	115.3679488	-2.326343493	3.54E-05	0.000757158
LOC109202033	9.833676966	-2.330075068	0.00605937	0.039312937
LOC100702483	58.09528046	-2.335172258	1.10E-12	4.66E-10
clvs2	38.74122714	-2.337653764	4.21E-06	0.000154547
kif23	6.040285207	-2.341313612	0.003089173	0.023757362
dusp13	90.52260709	-2.346898644	4.68E-11	1.19E-08
rimbp2	783.8031069	-2.349167398	9.63E-08	7.10E-06
agmat	500.0854738	-2.349245497	9.19E-08	6.98E-06
LOC109201938	8.901716341	-2.353942804	0.00149729	0.013798526
LOC102076968	10.32711551	-2.359749019	0.001114501	0.011084185
LOC102081495	9.309095619	-2.372764081	0.001151374	0.011317689
LOC100707422	234.4980195	-2.374092568	4.92E-13	2.29E-10
LOC100703256	493.1972375	-2.378895818	3.73E-07	2.14E-05
was	158.9077379	-2.408978084	8.80E-07	4.34E-05
tnfsf12	158.3044831	-2.410177747	8.47E-05	0.001539904
fbxl22	1784.266656	-2.410228341	0.004535626	0.031306429
LOC100708969	173.1134676	-2.41524297	4.91E-16	5.07E-13
LOC102076978	1294.397365	-2.418752538	3.30E-06	0.000127125
LOC100694730	10.44795225	-2.425957149	0.001369185	0.01291244
LOC100702269	786.1686272	-2.433299583	1.25E-11	3.56E-09
stxbp5	18.20326399	-2.433547537	0.001966777	0.016878326
LOC102075683	20.85981068	-2.435362939	4.99E-06	0.000175716
LOC100704464	5.691450204	-2.444138941	0.004873649	0.033086699
LOC100702599	77.41252655	-2.444745825	4.94E-08	4.19E-06
LOC100699981	399130.8225	-2.456635175	4.12E-18	7.65E-15
tecr	1251.879119	-2.456939887	3.97E-31	3.69E-27
tmem25	17.28767843	-2.467080347	0.00069311	0.007784608
LOC100709397	146.3753134	-2.467085607	4.22E-12	1.45E-09
LOC102078147	7.054668351	-2.467657652	0.000259161	0.003668918
LOC100692521	137.852079	-2.468711987	2.44E-05	0.000584581
LOC100692920	85.68679311	-2.472470112	9.79E-14	5.35E-11
LOC100701569	27.14406529	-2.473352959	7.93E-05	0.00146626
LOC100709620	265.6617365	-2.482304238	8.21E-06	0.000259459
pdgfc	134.5450211	-2.4901404	3.23E-05	0.00070508
LOC109194277	30.12241377	-2.495127569	2.07E-07	1.30E-05
LOC100710107	5.000095172	-2.496065106	0.001951785	0.016803971

LOC106097029	5.710460721	-2.504911858	0.00536811	0.035674803
LOC100709321	62.83758108	-2.505655472	4.25E-06	0.000155974
LOC100703124	59.43149771	-2.508234106	2.89E-06	0.000114423
nrxn1	23.42895234	-2.512661615	1.94E-08	1.91E-06
LOC102078820	171.9916615	-2.531879206	0.003242514	0.024640983
crispld1	42.97665659	-2.532480905	1.10E-09	1.70E-07
LOC102082117	11.47380054	-2.534350865	0.001185367	0.011529881
LOC109204297	9.35969713	-2.53565282	0.000599756	0.006972027
LOC102077293	337.6748546	-2.537229404	0.002874939	0.022444083
LOC112842792	28.93724095	-2.537520852	0.001396262	0.013107942
LOC100708507	5.355088378	-2.53912328	0.007027843	0.043807359
LOC106098841	204.7398347	-2.572190399	1.57E-11	4.42E-09
col1a1	27733.83925	-2.575288817	1.37E-06	6.21E-05
LOC100711833	18.01099426	-2.577702308	0.000224433	0.003295229
LOC109194832	7.352852623	-2.588634555	0.000280191	0.003872259
LOC100696264	156057.6216	-2.601584813	1.86E-12	7.37E-10
LOC112847075	12.5912592	-2.604175597	4.81E-06	0.00017342
LOC100701083	9.866243896	-2.610432662	0.003502908	0.026098816
rbfox3	9.779002589	-2.611968279	0.000541052	0.006426248
LOC100697625	5.479830491	-2.614211991	0.003972876	0.028623186
LOC102081083	7.580373294	-2.615394985	0.00482922	0.032869112
LOC100710931	8.858314823	-2.62066142	4.81E-05	0.000969715
LOC102075547	24.20000927	-2.621379761	0.000308477	0.004182323
LOC109195677	11.71958288	-2.633397933	0.004402897	0.030663564
LOC100710987	13008.82726	-2.634590878	0.00589457	0.03851257
slc1a4	41.51499215	-2.642795729	1.83E-09	2.53E-07
ppp1r36	9.409212059	-2.654460555	0.00150573	0.01384884
lrrc66	13.63790356	-2.65973744	9.26E-06	0.000284917
LOC100694784	267.6300293	-2.663761469	1.86E-17	2.48E-14
LOC100710219	144.5163904	-2.66384399	1.86E-05	0.000474836
aipl1	37.39252044	-2.665375289	1.05E-13	5.43E-11
asb12	528.4334994	-2.716292477	6.42E-14	3.98E-11
LOC100690947	91.12247442	-2.721955251	7.16E-12	2.15E-09
LOC109195962	8.997756876	-2.725401514	0.002648326	0.02107324
LOC112841688	52.77562728	-2.73147944	2.00E-09	2.73E-07
fbxo39	99.42038575	-2.751256989	7.15E-12	2.15E-09
LOC100706127	15.50934004	-2.779446115	8.68E-05	0.001570507
LOC100708991	100.9405294	-2.794283205	4.57E-06	0.000165423
LOC100691042	7167.003706	-2.802791832	0.003094574	0.023789055
LOC100707599	1686540.455	-2.808928345	1.04E-18	2.15E-15
LOC102075610	16.92072855	-2.812404441	3.96E-06	0.000145957
actc1	97456.21097	-2.84143482	0.00336617	0.02536294
LOC100708367	16.66844587	-2.841703535	3.65E-05	0.000775088
adamts16	82.76973107	-2.845080559	9.54E-08	7.07E-06
LOC100694402	5672.229618	-2.847491193	6.75E-09	7.89E-07
LOC112847185	7.242427877	-2.849946753	0.003420252	0.025667378
LOC102082846	7.189637132	-2.852184605	1.60E-05	0.000429204
LOC109202611	7.014249506	-2.861226042	0.00135067	0.012796255

LOC112846048	372.3533636	-2.861555297	0.000396662	0.005043202
LOC100703170	4249.960322	-2.936914283	8.97E-10	1.42E-07
zdhhc2	343.8846314	-2.954839586	0.000108246	0.001866497
dnm1	31.55914353	-2.957816668	5.44E-09	6.53E-07
LOC102079625	6.945094428	-2.967094989	0.004203133	0.02980841
LOC102078027	275.0651327	-2.970327062	4.82E-21	1.79E-17
sln	95084.56977	-2.975334881	3.42E-05	0.000737594
mcm5	13.29607959	-2.998583999	0.000845942	0.009036991
mapk15	10.27864108	-3.00367776	0.007616047	0.046445893
pdlim3	1169.418584	-3.048643564	8.90E-05	0.001600624
kbtbd3	24999.9351	-3.049520376	0.000837507	0.008991925
LOC109199784	5.220832716	-3.055030785	0.005103936	0.034307509
LOC102076225	22.17648002	-3.056962793	6.72E-07	3.50E-05
LOC100708028	95.76662785	-3.062644956	0.004580914	0.031548733
LOC100710568	118.4645557	-3.075337299	2.25E-26	1.40E-22
akap6	133.9019135	-3.093351351	8.23E-09	9.22E-07
LOC100698500	9.596228668	-3.124120047	9.80E-05	0.001720322
malrd1	14.12862051	-3.126075423	7.88E-06	0.000251739
LOC100705462	133.4459082	-3.134798599	7.31E-14	4.39E-11
stc2	275.5019787	-3.145789999	0.000361319	0.004723066
LOC100693531	5.609667115	-3.148647396	0.002593519	0.020743689
LOC106099005	32.44036122	-3.175496143	0.007851879	0.047494543
LOC112844053	15.46819158	-3.17881398	7.69E-06	0.000247386
LOC102079235	34.41741975	-3.197958941	7.45E-05	0.001395358
LOC100699717	1228.477191	-3.223582881	1.64E-12	6.62E-10
LOC106096824	167059.3959	-3.247097431	1.19E-17	2.02E-14
LOC100706218	44.37811542	-3.262035117	1.17E-05	0.000334594
cpb1	79.06622582	-3.304573004	5.06E-12	1.68E-09
msrb2	17.45301957	-3.308632447	6.14E-06	0.000206408
LOC106097519	40.47624901	-3.309874262	2.28E-06	9.40E-05
grb14	74.89617121	-3.319782499	9.45E-08	7.07E-06
LOC102077820	19.89135701	-3.340681394	3.18E-05	0.000700082
LOC112845094	79.19750901	-3.350436502	6.72E-06	0.000221079
LOC112843301	269.879327	-3.374823199	1.52E-05	0.000412047
LOC102080852	105.6930363	-3.393728216	2.36E-11	6.45E-09
LOC100710368	21.59507232	-3.398744544	2.89E-05	0.000653506
cdca7	13.38532098	-3.451946887	0.000681597	0.007693333
LOC100694538	7.61921552	-3.520201366	0.000459033	0.00565067
LOC106098123	4.976033586	-3.53018653	0.00053645	0.006379735
robo3	239.1891929	-3.573100272	2.12E-09	2.88E-07
LOC109199529	5.853753467	-3.615574293	0.000251153	0.003577316
LOC100698217	41.29242674	-3.624596991	6.31E-07	3.30E-05
mfap2	76.78303931	-3.631763282	6.33E-05	0.001219882
LOC100707179	9.049870952	-3.669845311	1.08E-05	0.000315568
LOC112846028	25.25000909	-3.692931493	1.93E-06	8.27E-05
gucy1a1	27.24475446	-3.740878837	6.61E-12	2.12E-09
fam131a	5.638426722	-3.788019669	0.002083327	0.017588876
LOC100705258	12.23415402	-3.8288644	4.97E-06	0.00017535

LOC109203197	63.30116454	-3.843034971	5.44E-10	9.89E-08
LOC102082099	14.63668699	-3.869940018	6.44E-06	0.000213307
LOC109199512	61.8128889	-3.886621259	4.43E-05	0.000904338
LOC100703626	7967.082145	-3.904553653	2.91E-07	1.76E-05
LOC112847497	175.6341051	-3.969096236	6.73E-24	3.13E-20
LOC109194892	22.17750993	-4.005739852	2.14E-10	4.42E-08
LOC112847058	7.04332437	-4.007906402	4.97E-06	0.00017535
slc6a17	20.6968666	-4.09042892	3.30E-09	4.23E-07
LOC100689923	3145.200571	-4.091747479	1.79E-06	7.76E-05
LOC102075530	123.0997217	-4.11778408	2.14E-14	1.53E-11
LOC100703503	24.30943063	-4.161085543	1.75E-06	7.62E-05
fhl2	574.8066823	-4.192649378	0.000180227	0.002764083
LOC106098646	16.76455541	-4.260981868	9.31E-06	0.000286069
LOC112847937	7.246554403	-4.357099286	5.01E-07	2.75E-05
susd2	6.047784397	-4.368114313	0.000101932	0.00177075
LOC109199736	46.3435968	-4.430951397	4.88E-08	4.16E-06
LOC100691886	102.0498047	-4.517436545	0.000295153	0.004028118
LOC100694717	84.1267026	-4.773986965	2.50E-05	0.000596419
LOC100698459	11.55962365	-4.781780891	1.92E-08	1.90E-06
LOC112842171	7.598159621	-4.810479279	0.000356749	0.004673442
pdzrn4	8.304608163	-4.868510775	0.000162051	0.002546241
LOC100695053	21.76335325	-4.92711772	8.45E-10	1.38E-07
LOC100534411	8171.9129	-4.94723996	0.000253488	0.003599568
LOC109195441	290.5693242	-5.097325049	3.97E-16	4.34E-13
LOC109195352	173.3861224	-5.196969583	2.56E-19	6.50E-16
LOC109199871	8.586445869	-5.421598244	1.03E-05	0.000310366
LOC100706423	10.941579	-6.237468061	3.40E-10	6.66E-08
LOC100712462	5614.818637	-6.281066636	1.38E-07	9.36E-06
LOC102080134	12567.63628	-6.610493336	1.22E-14	9.47E-12
LOC100701201	11.29558614	-6.775018107	7.54E-11	1.80E-08

Table S5. Selected mRNA primer sets used for qRT-PCR validation.

Primer name (forward-F, reverse-R)	Primer sequence (5′–3′)	Annealing temperature (°C)
pdzrn4 F	GCCTGAGTGTGTATCGCCCA	
pdzrn4 R	TTGTAGTGTTCCTCCCGTTTCT	60
gucy1a1 F	GCGAAAAATCTGAGCCCCA	
gucy1a1 R	CTGAGCCCGTGTCCTACTTG	60
pdlim3 F	CCAAACCAACTACGCCCAC	
pdlim3 R	TCACCACTGTCCCAACAATCC	58
stc2 F	TGTCGGAGACGCAATGTTTG	
stc2 R	CTTTCTGGCTCGCAGGTTTC	61
fgf14 F	CCAAGCCATTAGAAGTTGCCAT	
fgf14 R	CCTCCGTTCATCACTGCCG	60
myod1 F	GGTGGGTGAAGCAGGCACT	
myod1 R	TTGGTAAATCAGGTTGGGGTCC	58
igfbp2 F	CTTCCCCTGGAGCAACTGG	
igfbp2 R	CGGCGTCTTTATTGGGCTTT	60
fhl2 F	GACGACTTTGCCTACTGCCT	
fhl2 R	TGCCACCAAGACCGCTAATG	58
chrna1 F	ACCCGCCATCTTCAAAAGC	
chrna1 R	TTCATCACCCATTCTCCGCT	60
spire2 F	CTGAAGCGGGGGAAGGTT	
spire2 R	GGCTCTTTGGAGTGCTGTGAA	60
map7 F	TCCTGTTCTCTCATTGCCCC	
map7 R	GGTTTCTGTGGAGGTCTGCC	58
hspbp1 F	TTGGTGGGCTGGAGTTGTG	
hspbp1 R	TGGGGTTTGGGTCTGAATCTG	58
uchl1 F	TTAGCAACAATGGAGTGGACC	
uchl1 R	AGGGGAAGAGCAACATCAAGG	58
dnaaf2 F	ACCCCCTTGCCTTTCCATAC	
dnaaf2 R	GGCTTGGTTGGCTCTCTGAT	61
eftud2 F	GTGGGGCGTCTCTGGATTTC	
eftud2 R	TGTGATTGTGGCGGTCTTGA	58

Table S6. Selected differentially expressed miRNAs for qRT-PCR validation.

miRNA	Base mean	Log_2 fold change	p_{adj}
oni-miR-202	23.6763	22.0994	8.85E-13
oni-miR-10819	292.782	1.59861	0.00103
oni-miR-34	386.53	1.49018	0.02506
oni-miR-130b-5p	71098.7	1.18945	0.00146
oni-miR-21	459421	-1.1305	4.10E-16
oni-miR-99b	25607.1	-1.1656	0.00044
oni-miR-217	419.265	-1.7075	0.01152
oni-miR-153c	16.2735	-7.7856	0.00042

Supplementary Figures

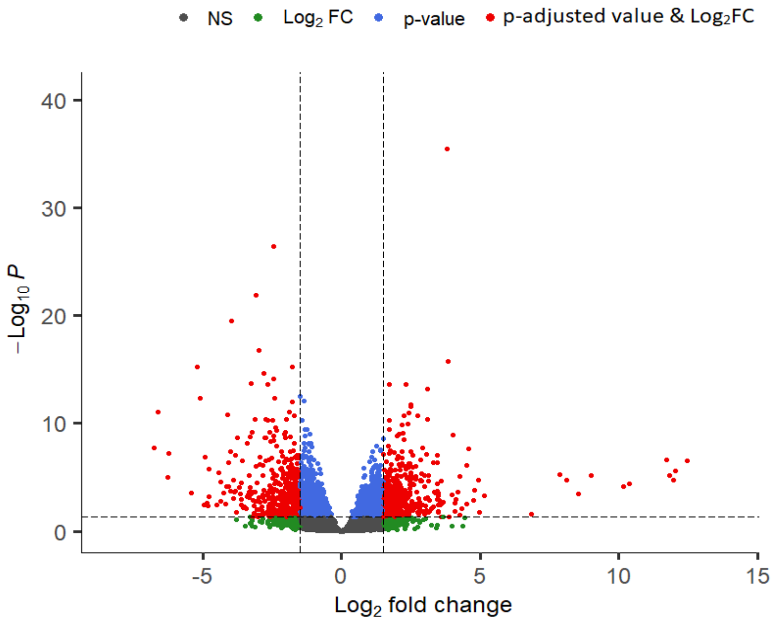

Fig S1. Volcano plot of differentially expressed mRNAs between BM (Fast-growing male) and SM (Slow-growing male) groups

Red dots represent up- and down-expressed circRNAs with a *p-adjusted value* below 0.05 and |Log_2fold change| ⩾1. Non-significant genes are marked with black and green dots, while blue dots represent genes having *p-adjusted value* below 0.5 but |Log_2fold change| <|1.

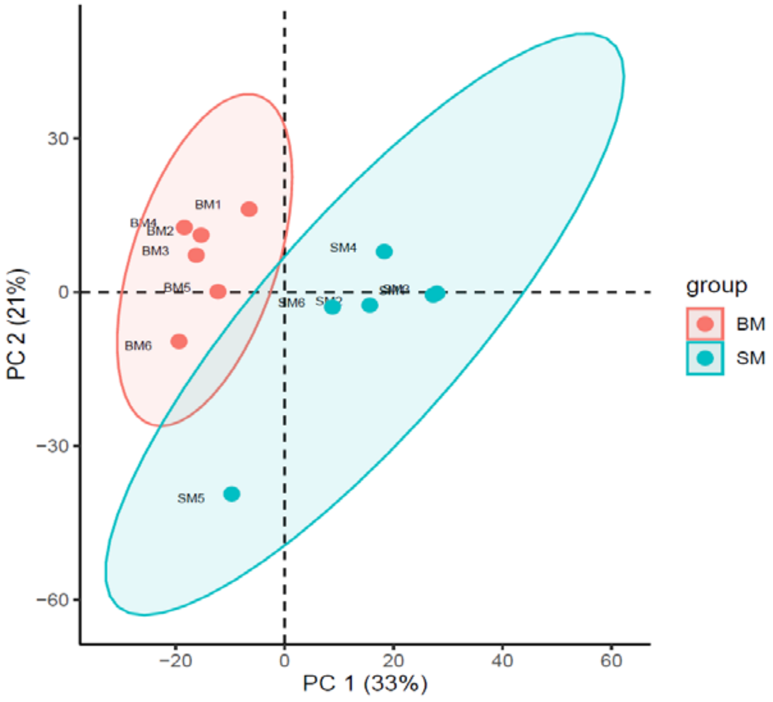

Fig S2. Principal component analysis (PCA) of mRNA-seq data

The PCA plot shows separate clustering of fast-growing (BM) and slow-growing (SM) male datasets based on gene count (FPKM). Light blue and light red circles represent SM and BM, respectively.

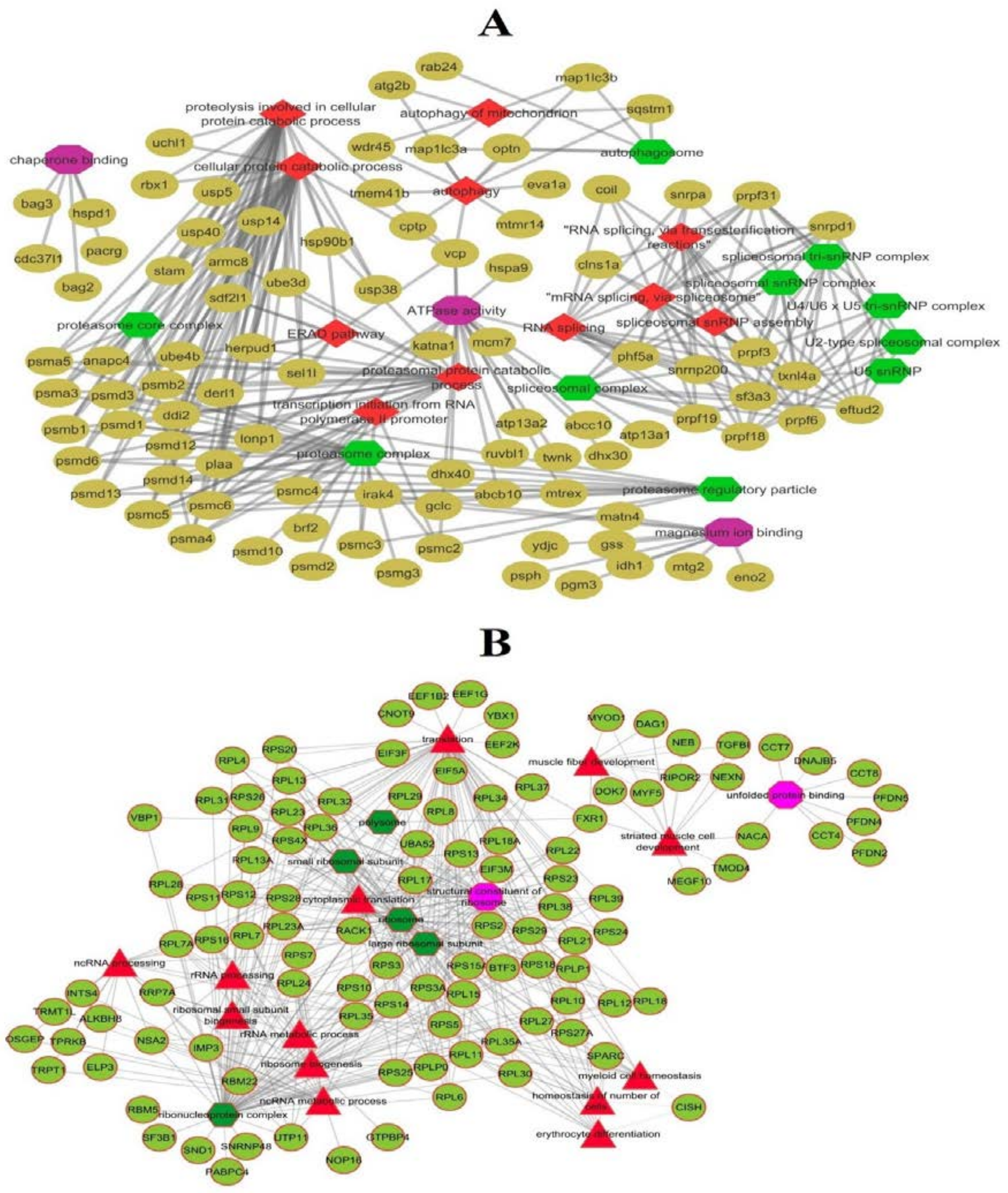

Fig S3. Network of the gene involved in different GO terms

A) Different GO terms and up-regulated genes in slow-growing (SM) compared to fast-growing males (BM). The circles represent genes, while diamonds, hexagons, and octagons represent biological process, molecular function, and cellular component, respectively. **B)** GO terms and down-regulated genes in slow-growing males compared to their fast-growing counterparts. The circles represent genes, while triangles, hexagons, and octagons represent biological process, molecular function, and cellular component, respectively.

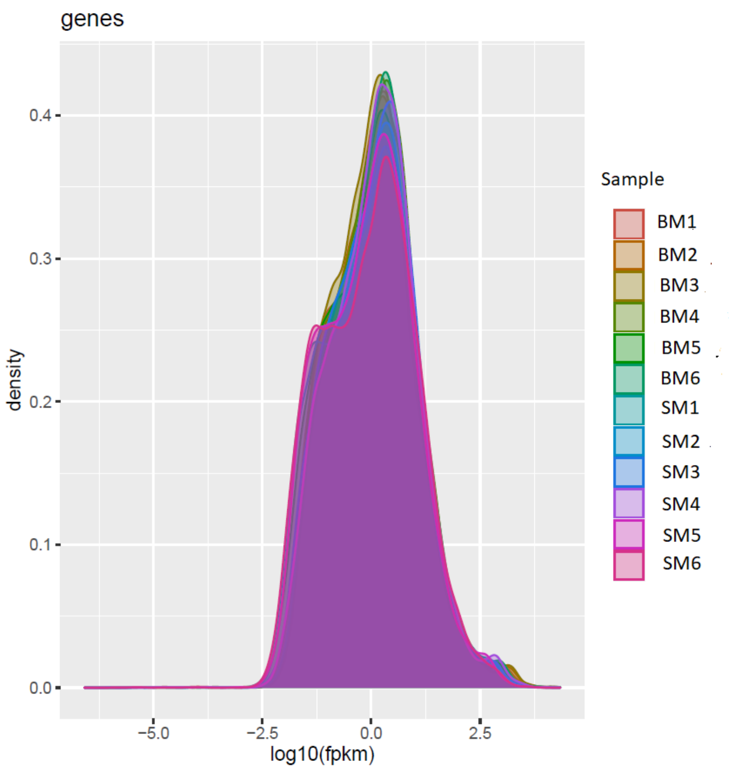

Fig S4. Distribution of mRNA expression levels

Expression distribution for all mRNA isoforms in two experimental groups BM (fast growing male) and SM (slow-growing male) in FPKM (fragments per kilobase of transcript per million fragments mapped).

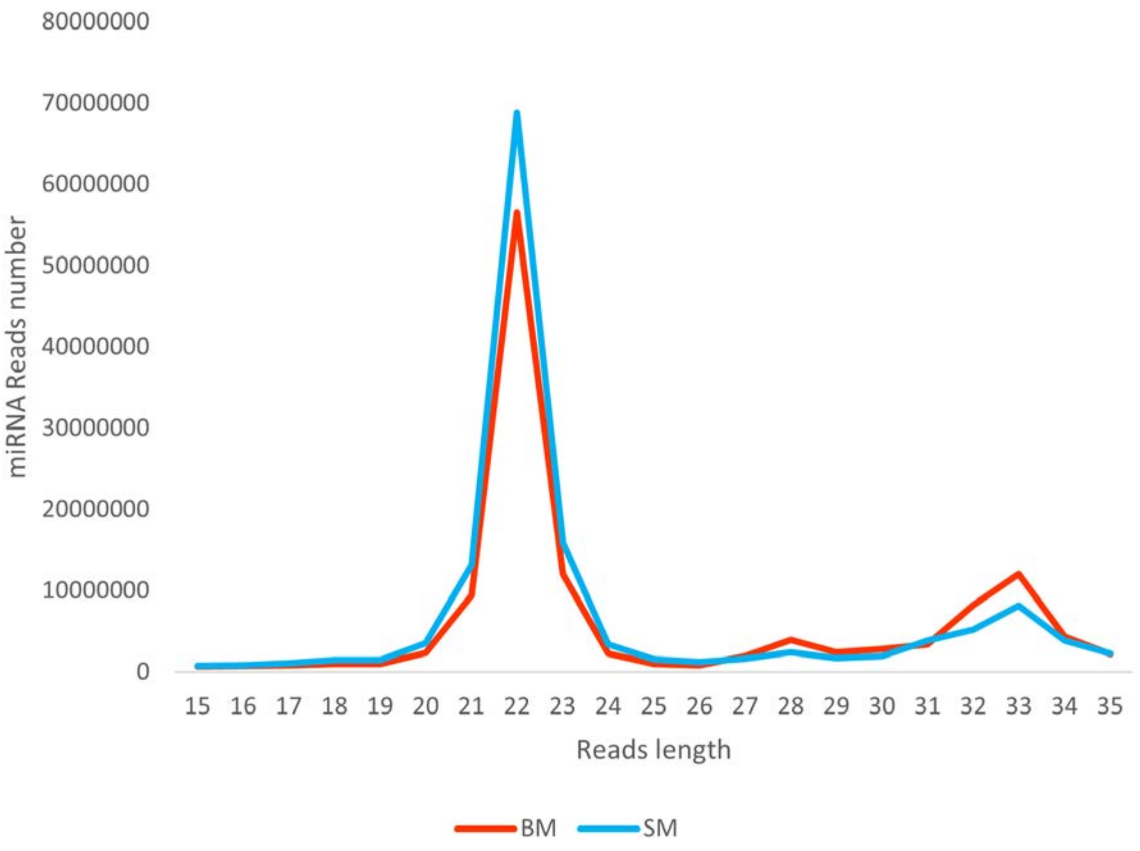

Fig S5. miRNA library profile

Length distribution of miRNAs. Light red and light blue lines represent the BM (fast-growing males) and SM (slow-growing males) groups, respectively. The X- axis represents the miRNA sequencing read length in base pairs (bp) after adapter trimming, while the number of reads is shown on the Y-axis.

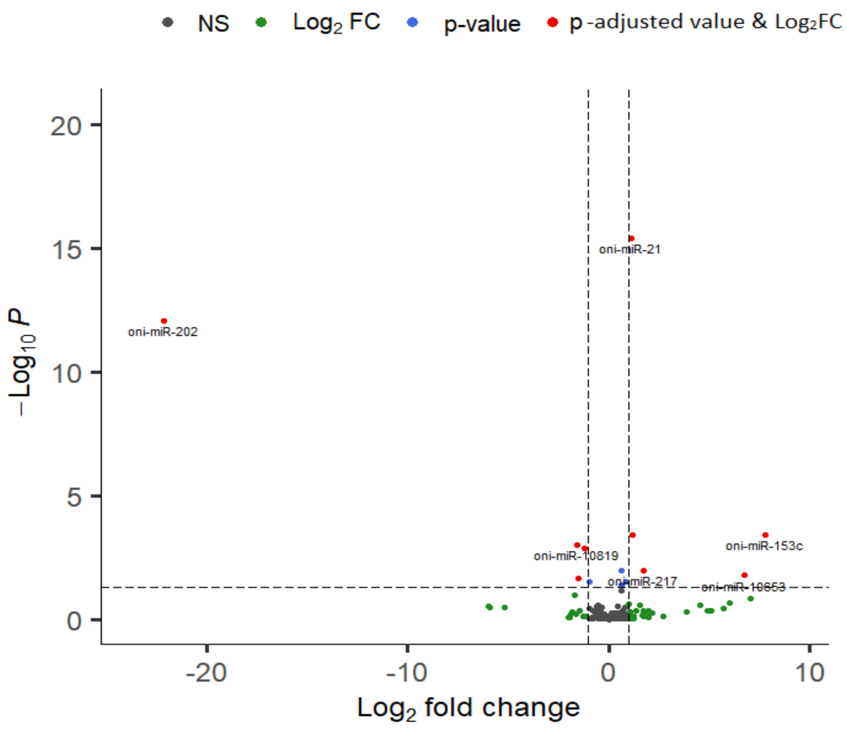

Fig S6. Volcano plot of miRNA expression

Volcano plot of differentially expressed miRNAs between BM (fast-growing males) and SM (slow-growing males) groups. X- and Y-axis represent Log_2fold change and p-adjusted value, respectively. Red dots represent up- and down-expressed circRNAs with a p-adjusted value below 0.05 and $|Log_2\text{fold change}| \geqslant |1|$. Non-significant genes are marked with black or green dots, while blue dots represent genes having *p-adjusted value* below 0.5 but $|Log_2\text{fold change}| < |1$.

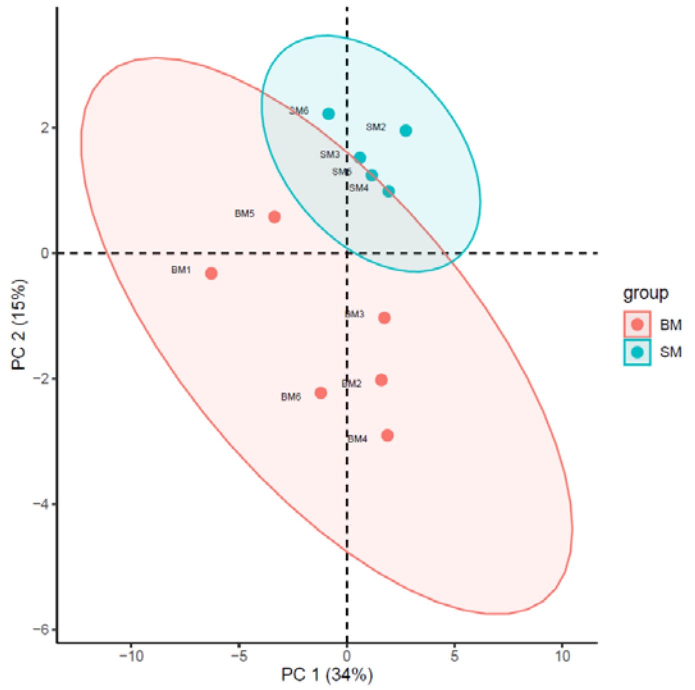

Fig S7. Principal component analysis of miRNA data

Principal component analysis (PCA) shows the clear clustering of individual replicates from each group, based on miRNA read count. X-axis is denoted as principal component 1 (PC1), and Y axis as principal component 2 (PC2); light blue and light red circles represent the SM (slow-growing male) and BM (fast-growing male) groups, respectively.

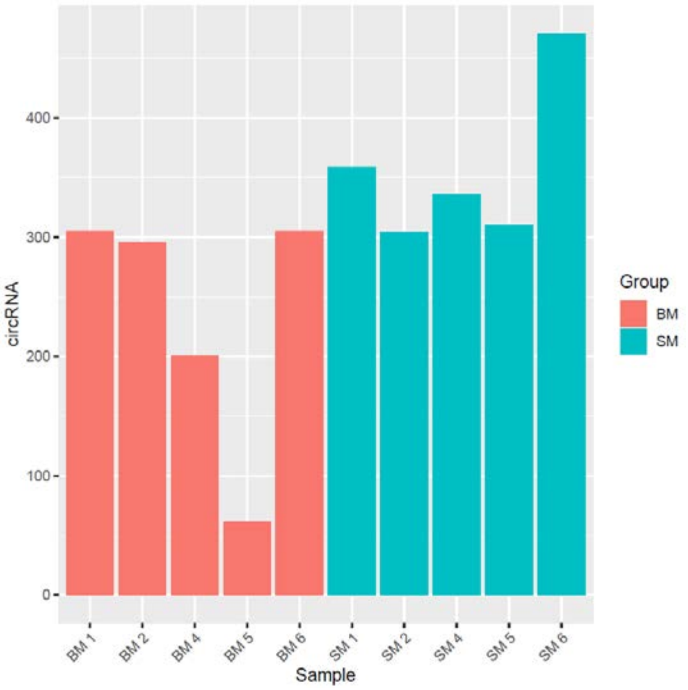

Fig S8. Distribution of circRNAs

The light red and light blue bars represent the fast-growing (BM) and slow-growing (SM) male groups, respectively. X-axis and Y-axis show the sample ID, and the number of circRNAs identified, respectively.

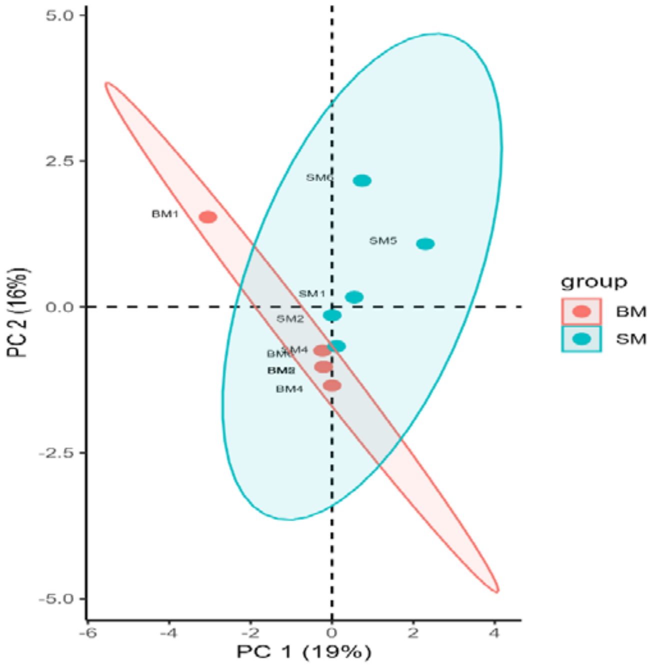

Fig S9. Principal component analysis of circRNA data

Principal component analysis (PCA) shows the grouping of fast-growing (BM) and slow-growing (SM) male groups based on junction reads count. The X-axis is denoted as principal component 1 (PC1) and Y-axis as principal component 2 (PC2); light blue and light red circles represent SM and BM groups, respectively.

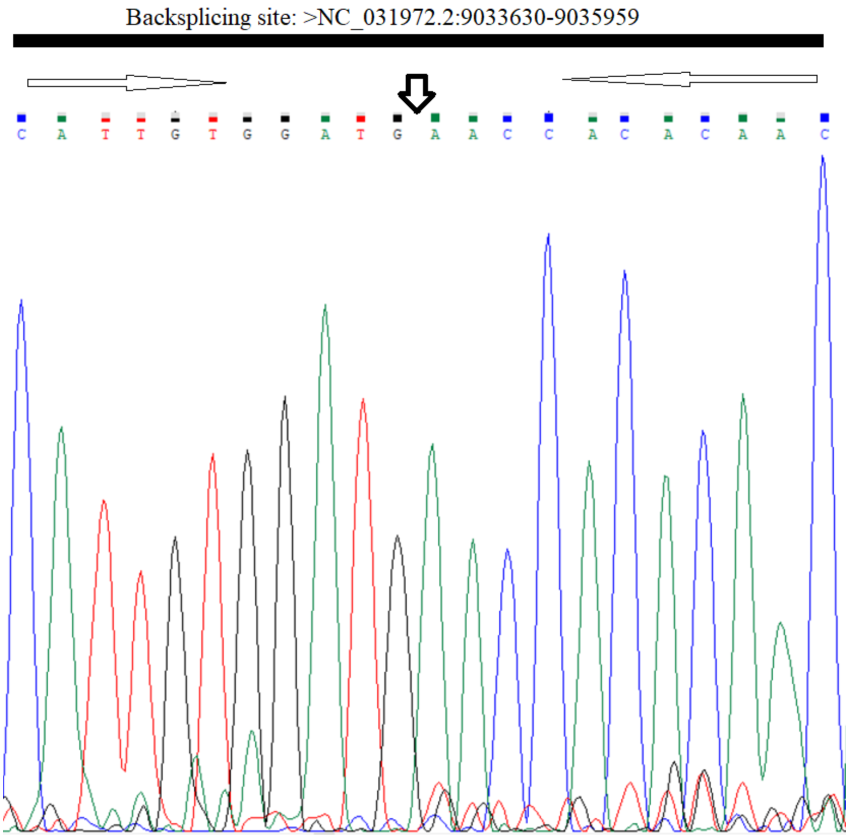

Fig S10. Validation of back-splicing junction in circMef2c by Sanger sequencing

Image of a partial Sanger sequencing electropherogram of a circMef2c amplicon. The arrow indicates the back-splicing /junction point.

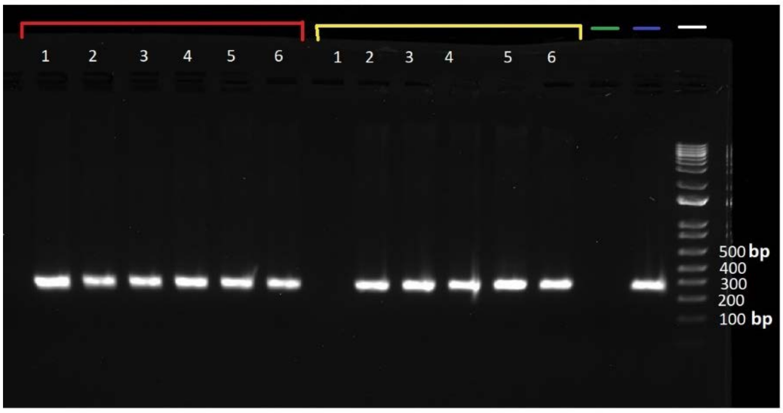

Fig S11. Agarose gel-electrophoresis of circMef2c PCR products

The white bar represents 1 Kb Plus DNA Ladder (bp = base pair), while red and yellow bars represent slow-growing male and fast-growing male groups, respectively. The green bar represents the negative control, and the positive control is marked in blue.

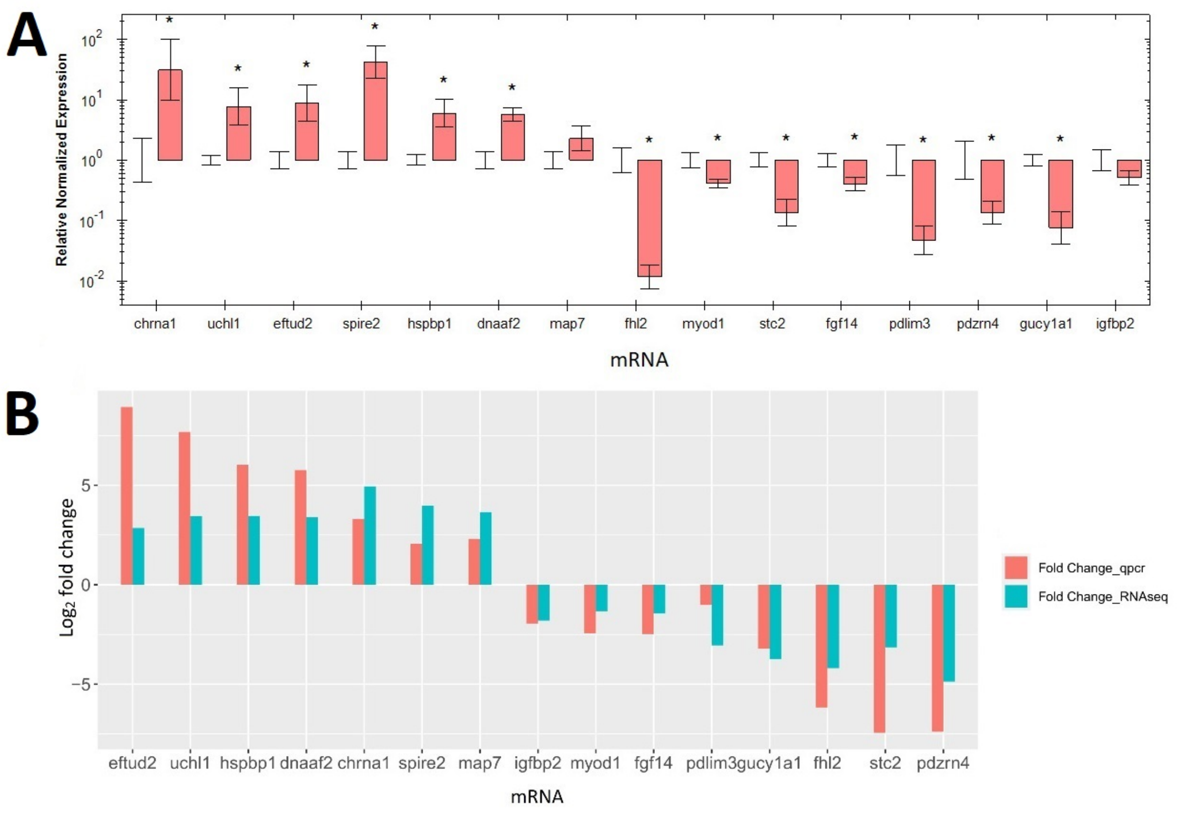

Fig S12. Validation of mRNA expression by qPCR

A) mRNA expression was quantified relative to *β-actin and elongation factor 1-alpha* expression level using the comparative cycle threshold method. Bars represent means ± SEM (*, $p < 0.05$). B) Real-time quantitative PCR (RT-qPCR) validation of RNA Sequencing (RNA-Seq) data. Comparison of Log_2 fold change between RNA-seq and RT-qPCR shows a similar expression trend.

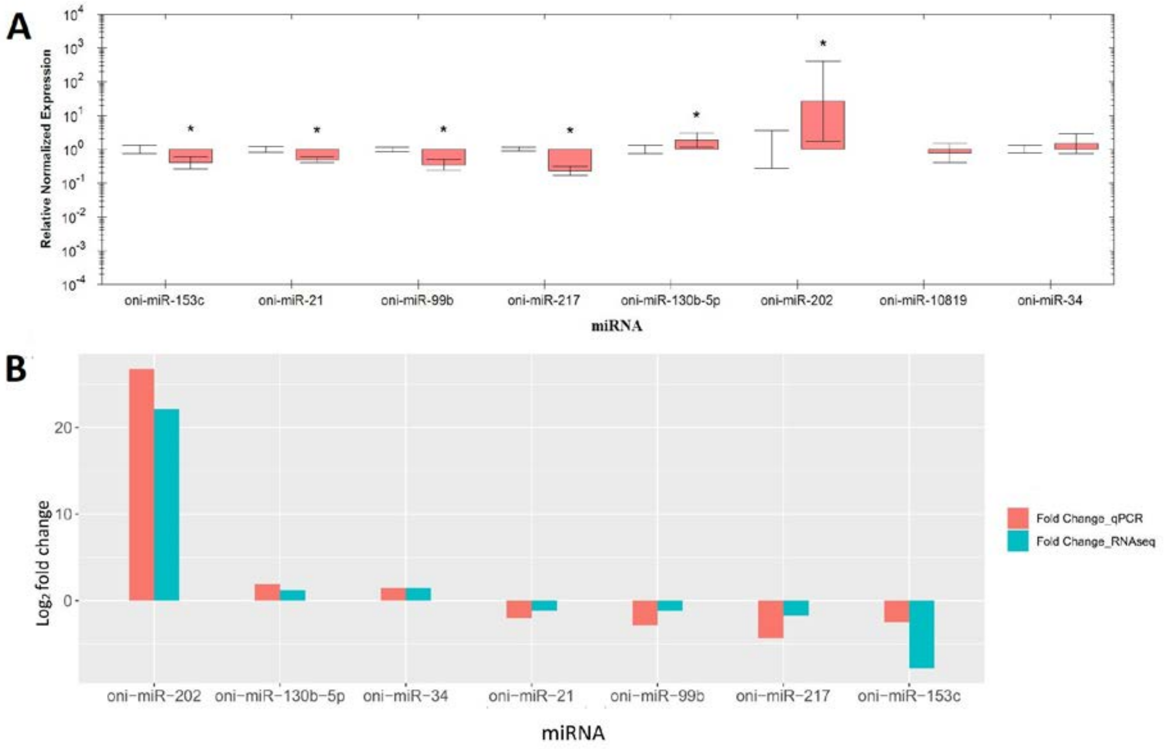

Fig S13. Validation of miRNA expression by qPCR

A) miRNA expression was quantified relative to oni-miR-10c and oni-miR-26b expression levels using the comparative cycle threshold method. Oni-miR-10819 was only found in the slow-growing male group. Bar represent means ± SEM (*, $p < 0.05$). B) Real-time quantitative PCR (RT-qPCR) validation of miRNA Sequencing (small RNA-seq) data. Comparison of Log_2 fold change between miRNA-seq and RT-qPCR shows a similar expression trend.
